# Aphid effectors suppress plant immunity via recruiting defense proteins to processing bodies

**DOI:** 10.1126/sciadv.adv1447

**Published:** 2025-07-16

**Authors:** Qun Liu, Anna C. M. Neefjes, Archana Singh, Roksolana Kobylinska, Sam T. Mugford, Mar Marzo, James Canham, Mariana Schuster, Renier A. L. van der Hoorn, Yazhou Chen, Saskia A. Hogenhout

**Affiliations:** ^1^ Department of Crop Genetics, John Innes Centre, Norwich Research Park, Norwich NR4 7UH, UK.; ^2^ Department of Plant Sciences, University of Cambridge, Cambridge CB2 3EA, UK.; ^3^ Plant Chemetics Laboratory, Department of Biology, University of Oxford, Oxford OX1 3RB, UK.; ^4^ College of Plant Science and Technology, Huazhong Agricultural University, Wuhan 430070, China.

## Abstract

Aphids are small insects that have developed specialized mouthparts and effector proteins to establish long-term relationships with plants. The peach-potato aphid *Myzus persicae* is a generalist, feeding on many plant species and capable of transmitting numerous pathogens. This study reveals how host-responsive cathepsin B (CathB) proteins in the oral secretions of *M. persicae* facilitate aphid survival by modulating plant immune responses. Host-responsive CathB proteins localize to plant processing bodies (p-bodies), cytoplasmic ribonucleoprotein granules involved in messenger RNA storage or decay. Upon localization, these CathB proteins recruit key immune regulators EDS1, PAD4, and ADR1 to these bodies, suppressing plant defenses. A plant protein, Acd28.9 (Hsp20 family), counteracts this CathB activity and contributes to plant resistance to aphids. These findings highlight an unexpected role for p-bodies in plant immunity and uncover a plant resistance mechanism to aphid infestation.

## Teaser

Aphids hijack plant immunity via p-bodies, but plants fight back with a heat shock protein in an unexpected twist on defense.

## INTRODUCTION

Aphids are among the most sophisticated feeders in the insect herbivore world. Through more than 150 million years of coevolution with plants (*1*), they have developed specialized mouthparts, known as stylets, which carefully navigate between plant cells to access the phloem (*2*). During this process, aphids deliver oral secretions (OSs), including a variety of effectors, directly into plant cells (*3*). The effectors help aphids modulate numerous plant processes and establish long-term feeding in the plant vascular tissues (*4*, *5*), particularly the nutrient-rich phloem that transports resources from source to sink organs.

The success of aphids is evident, as nearly all vascular plants host at least one aphid species, capable of rapidly reproducing, reaching large populations that can cover entire plant tissues. In addition, aphids are efficient vectors for numerous plant viruses and other pathogens, many of which are heavily reliant on aphids for transmission (*6*). Given this success, aphid effectors likely target key plant regulatory processes that not only enable aphids to thrive on plants but also provide a crucial advantage to the pathogens they transmit (*7*). Yet, how these insects manipulate plant processes remains largely unknown.

Among aphids, *Myzus persicae* is especially intriguing. While most aphid species and insect herbivores have specialized in colonizing one or a few plant species, *M. persicae* is an exceptional generalist, with one of the broadest host ranges among insects (*8*)—a feat even more remarkable given its predominantly clonal reproduction (*9*). Previous studies have shown that cathepsin B (CathB) family genes are differentially expressed in response to host change (*10*, *11*). Moreover, a CathB protein was found to be introduced into the plant host during aphid feeding and to modulate plant defense responses of *Nicotiana tabacum* (*12*), suggesting that CathB proteins are effectors that contribute to the *M. persicae* ability to colonize diverse plants.

Here, we demonstrate that CathB proteins suppress key immune regulators—enhanced disease susceptibility 1 (EDS1), phytoalexin deficient 4 (PAD4), and activated disease resistance 1 (ADR1)—by recruiting them to processing bodies (p-bodies), which are cytoplasmic ribonucleoprotein granules that are involved in mRNA storage or decay. Notably, a plant heat shock protein 20 (Hsp20) family protein counteracts this CathB activity and contributes to plant resistance to aphids. These findings reveal unexpected roles for p-bodies in immunity and open fresh avenues on how plants defend against insect attack.

## RESULTS

### Orally secreted, host-responsive CathB proteins promote aphid fecundity

**Fig. 1.**
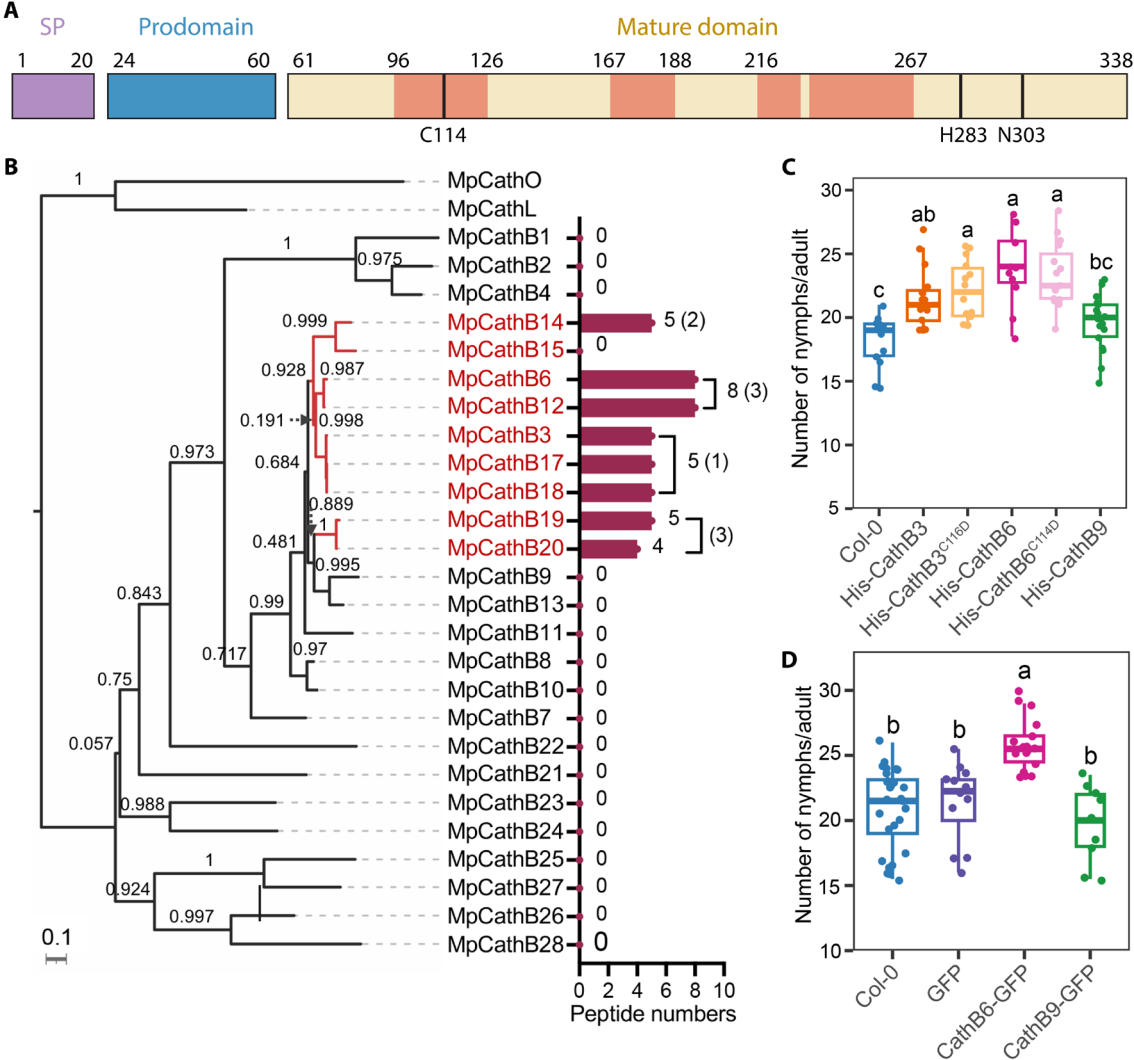
CathB proteins in *M. persicae* OSs promote reproduction on *A. thaliana*. (**A**) Structure of *M. persicae* CathB, with MS-detected peptides in *M. persicae* OSs highlighted in light red (fig. S1A). SP, signal peptide. Catalytic triad residues are marked. The first residue of the catalytic triad is lacking in CathB9 (fig. S3). (**B**) CathB peptides detected in *M. persicae* OS match host-responsive proteins showing CathB6 and CathB12 as highly abundant; bars indicate total (unique) peptides. The phylogenetic tree was generated on the basis of the alignment of 26 CathB proteins annotated as full-length (table S1) with one CathL and one CathO as outgroups. Scale bar, 0.1 substitutions per site; red branches indicate host-responsive CathB proteins. (**C** and **D**) Increased fecundity in aphids feeding on plants expressing CathB3, CathB6, or its GFP-tagged version (transgene expression levels, fig. S4, A and B). Box plots show fecundity per adult female (*n* = 9 to 28 replicates per line); letters indicate differences assessed by ANOVA (Tukey’s method, *P* < 0.05) (additional fecundity assays, fig. S5).

Proteomic analysis of *M. persicae* OS identified a total of eight CathB proteins, including CathB3, CathB6, CathB12, CathB14, CathB17, CathB18, CathB19, and CathB20 (*13*). Among these, CathB6 and CathB12 were the most abundant, followed by CathB3, CathB14, CathB17, CathB18, CathB19, and CathB20, based on numbers of unique peptides identified (Fig. 1, A and B, fig. S1A, tables S1 and S2). We previously identified these proteins as members of an expanded phylogenetic clade within the CathB gene family of *M. persicae* (*11*). Genes in this clade exhibit coordinated differential expression depending on the plant host species and are markedly up-regulated in aphids feeding on *Arabidopsis thaliana* (Fig. 1B and fig. S1B) (*10*, *11*). These host-responsive CathB proteins exhibit high sequence similarity, with only a few substitutions involving residues of similar biochemical properties (fig. S2). Aphid CathB proteins consist of three domains: a signal peptide, a prodomain, and a mature domain (Fig. 1A). The active protease form of CathB is comprised within the mature domain, which is released following the autocatalytic cleavage of the prodomain at acidic pH (*14*, *15*). The mature domain harbors the essential catalytic triad—cysteine, histidine, and asparagine—located on the substrate binding cleft, which is crucial for substrate catalysis (*14*, *15*). Only peptides corresponding to the mature domain were detected in aphid OS (Fig. 1A and fig. S1A).

Because it is unfeasible to functionally analyze all CathB proteins simultaneously, we started our investigations with two CathB proteins that corresponded to peptides abundantly detected in *M. persicae* that are highly expressed in the aphid and that belong to different clades in the CathB phylogeny (Fig. 1B and fig. S1B). These were CathB6 and CathB3. We also included CathB9 given that this one was not detected in the OS (Fig. 1B). Moreover, CathB9 lacks a key catalytic triad residue (fig. S3). Transgenic plants producing the mature domains of CathB6, or the closely related CathB3, showed increased aphid progeny, unlike CathB9 transgenic plants (Fig. 1, C and D, and figs. S4 and S5). CathB6 and CathB3 are both detected in *M. persicae* OS, unlike CathB9 (Fig. 1B). However, catalytically inactive CathB3^C116D^ and CathB6^C114D^ lines also supported higher aphid fecundity (Fig. 1C and fig. S5A), indicating that CathB peptidase activity that involves the catalytic triad in the mature domain is not required for the increased aphid fecundity phenotype.

### *M. persicae* CathB proteins localize in p-bodies in plants

**Fig. 2.**
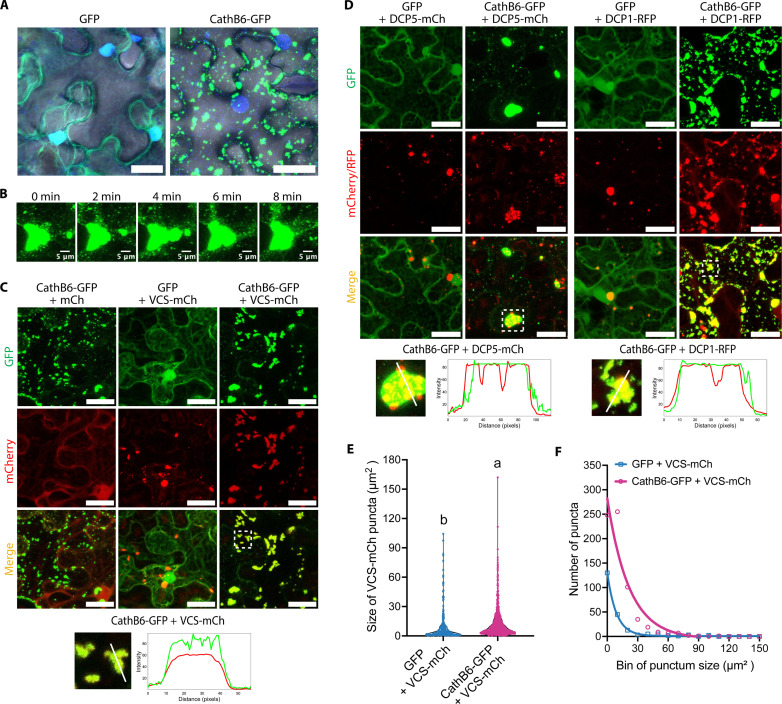
CathB6 forms mobile puncta and colocalizes with p-body markers VCS, DCP5, and DCP1 in *N. benthamiana* leaf cells. (**A**) Confocal images of CathB6-GFP–induced puncta (individual channels, fig. S6A). Nuclei are stained with DAPI (blue), and images were overlaid with a bright-field image showing the outline of the cell. (**B**) Time-lapse confocal images of the fusion of two CathB6-GFP puncta fusion (movie S1). (**C** and **D**) Confocal images of CathB6-GFP colocalization with VCS-mCherry (C), DCP5-mCherry (D), and DCP1-RFP (D) and intensity profiles below the images indicating overlap (more images and punctum size distributions, fig. S10, A to C). Scale bars, 30 μm. (**E** and **F**) Quantitative analysis of VCS-mCherry punctum size distributions across multiple cells from five independent leaf sections (derived from two plants), comparing the presence of CathB6-GFP versus GFP alone. Significance assessed by two-tailed Student’s *t* test. The bin size of puncta in (F) is 30 μm^2^.

We examined the subcellular localization of CathB6-GFP in *Nicotiana benthamiana* leaves. CathB6-GFP promotes aphid fecundity (Fig. 1D), indicating that the green fluorescent protein (GFP) fusion does not affect the CathB6 function. CathB6-GFP localized to punctate structures of varying sizes, large (>120 μm^2^), medium (5 to 120 μm^2^), and small (<5 μm^2^), in the plant cell cytoplasm (Fig. 2A and figs. S6, A to C, S7A, and S8A). Puncta were also observed adjacent to, but not within, plant cell nuclei (Fig. 2A and fig. S6, A and B). Puncta were mobile, and smaller puncta often fused with larger ones (Fig. 2B and movie S1), as also evidenced by GFP slowly re-emerging in the larger puncta upon photobleaching (fig. S6, D and E). Similar puncta were observed for CathB6-RFP, CathB6-GFP in the presence of red fluorescent protein (RFP), and CathB3-GFP (fig. S9, A and B). In contrast, CathB9-GFP exhibited a distribution more similar to that of free GFP, with small puncta only occasionally observed (fig. S9, A and B). CathB6-GFP puncta were also observed in *A. thaliana* protoplasts (fig. S9C).

We co-infiltrated CathB6 constructs with a series of cellular markers that have been reported to form puncta in the plant cell cytoplasm (table S3). CathB6-GFP colocalized with the p-body scaffolding protein varicose (VCS) (Fig. 2C and figs. S7B and S10A), decapping protein 5 (DCP5) (Fig. 2D and figs. S7B and S10B) and DCP1 (Fig. 2D and fig. S10C) (*16*–*18*). Whereas VCS-mCherry was depleted in areas occupied by YFP-DCP1 (fig. S10D), CathB6-GFP covered the areas of both VCS-mCherry (Fig. 2C and fig. S10A) and DCP1-RFP (fig. S10C). Similar results were obtained with different fluorescent tags (fig. S11A), and CathB6 also located to p-bodies in *A. thaliana* protoplasts (fig. S11B). CathB6-GFP puncta colocalized at lower frequency with the stress granule marker RNA-binding protein 47b (RBP47b) (fig. S12A) (*19*) and did not obviously colocalize with the autophagosome marker autophagy-related gene 8 (ATG8) (*20*, *21*) and immune regulator nonexpresser of pathogenesis-related gene 1 (NPR1) (*22*) (fig. S12, B and C). Therefore, CathB6 targets p-bodies and partially stress granules in plant cells. P-bodies are cytoplasmic ribonucleoprotein granules that are involved in mRNA storage or decay and can convert into stress granules in line with their role in balancing the storage, degradation, and translation of mRNAs in cells (*23*, *24*). In plants, p-bodies are enriched for mRNAs encoding proteins needed for photomorphogenesis (*25*).

Quantification of punctum size and number shows that CathB6 increases both the number and size of p-bodies (Fig. 2, E and F). In addition, small puncta in these cells colocalize with DCP5-mCherry up to 100% (fig. S10B) and at lower frequencies with VCS and DCP1 (fig. S10, A and C). In contrast, cells with medium- and large-sized puncta colocalized mostly with VCS-mCherry and DCP1-RFP (fig. S10, A and C). These data indicate that CathB6 promotes p-body formation, which is shown to occur when the translation initiation of mRNAs is blocked (*23*, *25*). In addition, given that DCP5 is the initial component that arrests mRNA and subsequently recruits other proteins, such as DCP1 and VCS, to assemble messenger ribonucleoproteins (mRNPs) to p-bodies (*25*, *26*), CathB6 may localize to p-bodies at a very early stage, potentially playing a role in initiating p-body assembly.

### CathB6 sequesters EDS1 and its partners to p-bodies

**Fig. 3.**
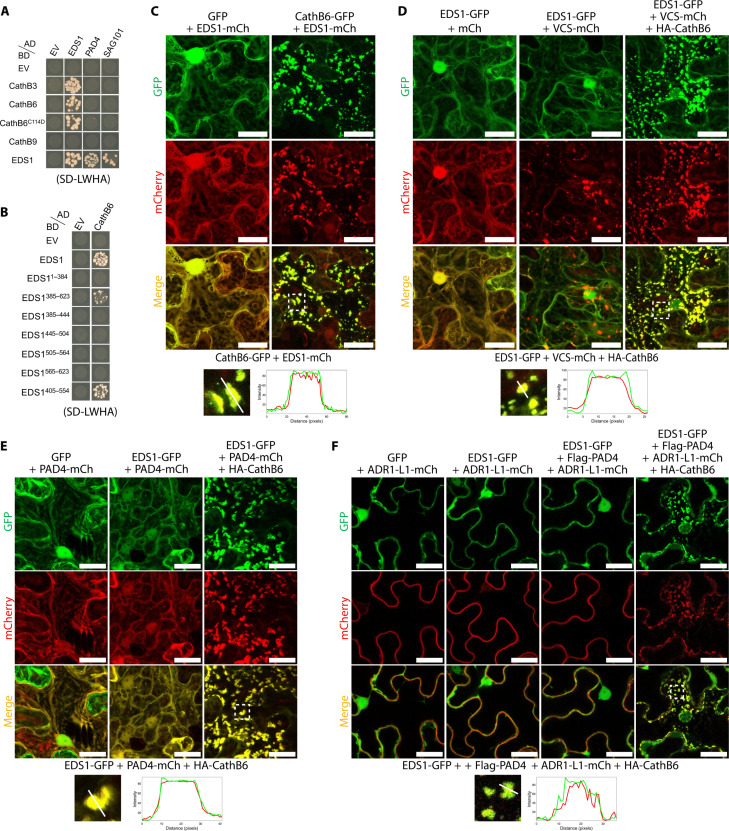
CathB6 interacts with EDS1 and relocates EDS1, PAD4, and ADR1 to p-bodies. (**A** and **B**) Y2H assays on selective SD-LWHA medium (yeast growth on SD-LW medium, fig. S14, A and B). (**C** to **F**) Confocal images illustrating protein localization and colocalization: (C) EDS1-mCherry localized in puncta with CathB6-GFP, (D) colocalization of EDS1-GFP and VCS-mCherry with HA-CathB6, (E) colocalization of EDS1-GFP and PAD4-mCherry in puncta with HA-CathB6, and (F) colocalization of EDS1-GFP and ADR1-L1-mCherry in puncta with Flag-PAD4 and HA-CathB6. Graphs below the confocal images are intensity profiles along the marked lines of the puncta indicated with white boxes in the confocal images above. Scale bars, 30 μm.

To identify potential plant targets of CathB6, we performed proximity labeling (PL)-mass spectrometry (MS) experiments on stable transgenic *A. thaliana* plants producing CathB6-TurboID fusion (*27*). This identified EDS1, with 12 unique peptides and an 8.015-fold enrichment (*P* value of 0.0072) compared to GFP-TurboID controls (fig. S13). EDS1 is a positive regulator of plant basal immunity, effector triggered immunity (ETI), and systemic acquired resistance (SAR), including accumulation of the defense hormone salicylic acid (SA) and *N*-hydroxypipecolic acid (NHP) and associated defense responses (*28*–*30*). We found that CathB6 directly interacts with EDS1 in yeast two-hybrid (Y2H) assays (Fig. 3A and fig. S14A), Cath6 pulls down EDS1 from *N. benthamiana* leaves (fig. S15A), and EDS1 pulls down CathB6 from insect cell extracts (fig. S15B). CathB6 interacts with a fragment (EDS1^405-554^) of the EDS1 EP domain (Fig. 3B). CathB3 and CathB6^C114D^ also interact with EDS1 (Fig. 3A and fig. S14A). However, CathB9 did not interact with EDS1 and none of the CathB proteins interacted with PAD4 and senescence-associated gene 101 (SAG101), whereas EDS1 did (Fig. 3A and fig. S14A), as expected (*31*). This result is consistent with the finding that CathB3, CathB6, and the CathB6^C114D^ mutant improved aphid fecundity, while CathB9 did not (Fig. 1, C and D, and figs. S4 and S5).

Confocal microscopy revealed that in the presence of GFP, EDS1-mCherry was distributed throughout the cytoplasm and in nuclei (Fig. 3C), consistent with the known nucleocytoplasmic distribution of EDS1 (*32*). However, in the presence of CathB6-GFP, EDS1-mCherry forms puncta that colocalize with CathB6-GFP (Fig. 3C, and figs. S7C and S8, B and C). Similar EDS1-GFP puncta were observed in the presence of CathB6-RFP (fig. S16A) and in *A. thaliana* protoplasts (fig. S16B). Moreover, EDS1-GFP was found to locate in puncta only in the presence HA-CathB6 (figs. S7D and S17A) and these puncta colocalize with VCS-mCherry (Fig. 3D and fig. S7E). Three partite colocalization with CathB6-BFP, EDS1-GFP, and VCS-mCherry as well as VCS-BFP, CathB6-GFP, and EDS1-mCherry showed that CathB6 colocalizes with EDS1 and VCS in p-bodies (fig. S18). CathB3 and CathB6^C114D^ puncta also colocalize with EDS1-mCherry (fig. S19). These data indicate that CathB6, CathB3, and CathB6^C114D^ recruit EDS1 to p-bodies.

In plants, EDS1 forms heterodimers with either PAD4 or SAG101, leading to pathogen restriction or a hypersensitive response (HR)/cell death (*28*). Both EDS1-GFP and PAD4-mCherry localized to the cytoplasm and nucleus (fig. S8D) but relocated to puncta only in the presence of HA-CathB6 (Fig. 3E and fig. S7F). In contrast, EDS1-GFP and SAG101-mCherry were localized to the nucleus in the absence of HA-CathB6, and in the presence of HA-CathB6, these proteins remained in the nucleus and did not form puncta in the cytoplasm (figs. S7F and S17B). SAG101 may largely deplete EDS1 from the cytoplasm, making it unavailable for CathB6 to be recruited to p-bodies.

EDS1 and PAD4 depend on ADR1 for downstream signaling (*33*), and ADR1 also associates with the EDS1 and PAD4 complex (*28*, *34*). We found that *A. thaliana* ADR1-L1 locates to puncta colabeled with EDS1-GFP in the presence of Flag-PAD4 and HA-CathB6 (Fig. 3F and fig. S7G). Therefore, CathB6 not only recruits EDS1 to p-bodies but also its partners PAD4 and ADR1. Given that CathB6 interacts directly with EDS1, but not with PAD4 or ADR1, it is likely that PAD4 and ADR1 are recruited to p-bodies through their association with EDS1.

### CathB6 modulates *A. thaliana* immunity through the EDS1-PAD4 pathway

**Fig. 4.**
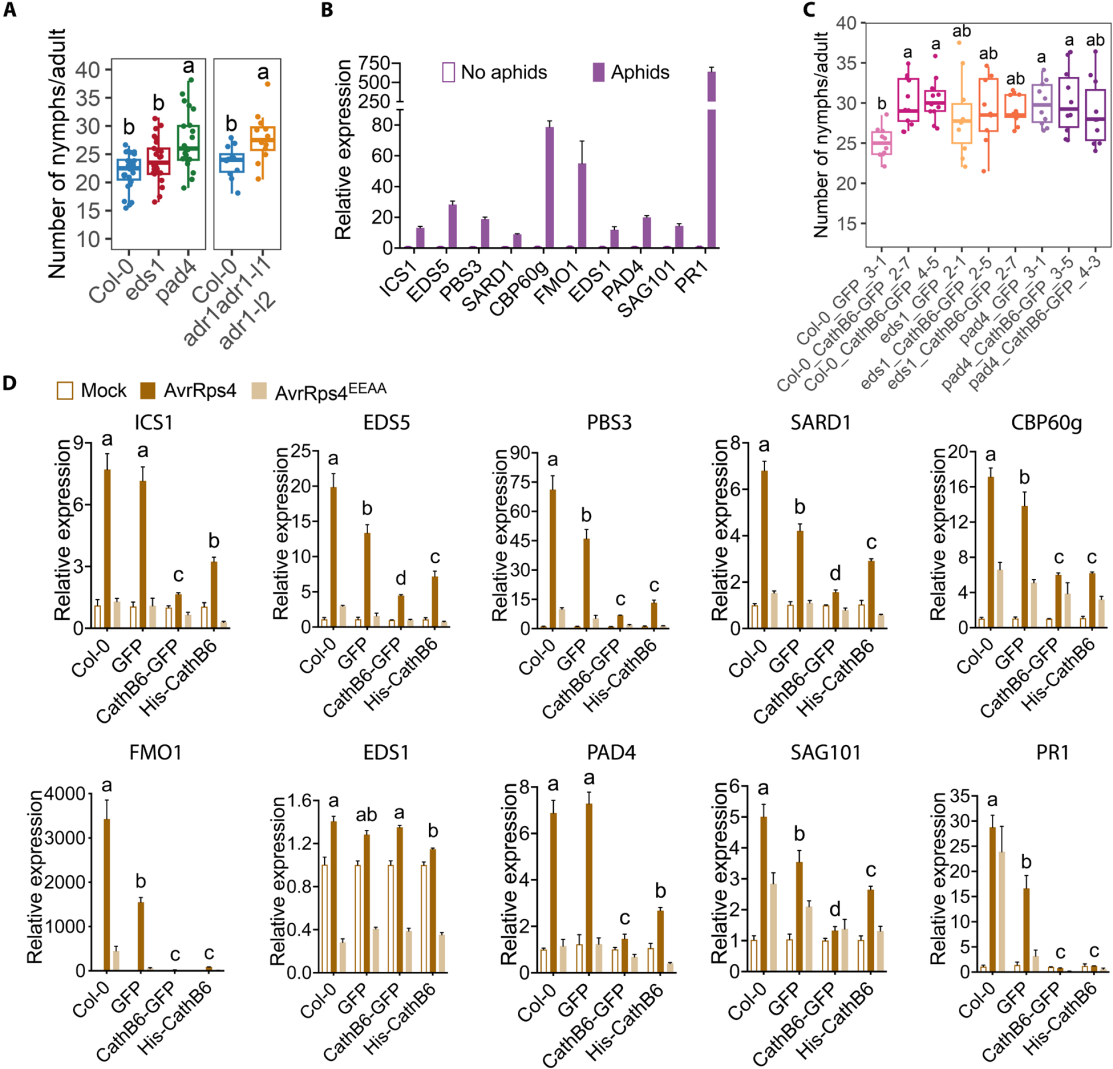
CathB6 inhibits the expression of EDS1-regulated genes involved in the SAR pathway. (**A**) *M. persicae* fecundity is significantly improved on *eds1*, *pad4*, and *adr1 adr1-l1 adrl-l2* mutant *A. thaliana* plants. (**B**) Aphids feeding on Col-0 plants induce the up-regulation of EDS1- and SAR-related transcripts, including SA biosynthesis (ICS1, EDS5, and PBS3) and SAR regulators (PAD4, SAG101, SARD1, CBP60g, FMO1, and PR1). (**C**) *M. persicae* fecundity assays on *A. thaliana* lines (transgene expression levels, fig. S4C). Box plots show fecundity per female aphid (*n* = 9 to12 replicates per line); letters indicate differences determined by ANOVA (Tukey’s method, *P* < 0.05). (**D**) Expression of *A. thaliana* EDS1- and SAR-related genes in response to mock, Pf0-1 AvrRps4, or Pf0-1 AvrRps4^EEAA^ treatments. Data represent the means ± SEM of data of two biological replications; significant differences were analyzed by ANOVA (more independent replicates, fig. S21).

To examine whether EDS1-PAD4-ADR1–mediated immunity signaling contributes to aphid resistance, we conducted aphid fecundity assay with *eds1-2*, *pad4-1*, and *adr1 adr1*-*l1 adr1*-*l2* mutant *Arabidopsis* plants. Aphid reproduction was slightly improved, although not significantly, on *eds1-2* plants and significantly improved on *pad4-1* and *adr1 adr1*-*l1 adr1*-*l2* plants (Fig. 4A), suggesting that the EDS1-PAD4-ADR1 module is involved in plant immunity against aphids. The module regulates the expression of genes in both the SA and NHP pathways (*28*, *33*, *35*), and we found that these genes were consistently up-regulated in response to aphid feeding (Fig. 4B and fig. S20). However, the magnitude of up-regulation varied substantially across biological replicates (Fig. 4B and fig. S20). This variation may arise from several factors. First, aphid feeding behaviors—ranging from brief probing to sustained phloem ingestion—differentially activate plant defense genes (*36*), and the proportion of aphids engaged in each feeding mode likely varies across biological replicates. In addition, individual aphids may vary in the type and amount of effector they deposit in their OS, which can either enhance or suppress host defense responses (*37*). Because of this variation, we were unable to reliably assess the specific impact of CathB6 on the expression levels of these defense-related genes (fig. S20).

We generated stable CathB6-GFP transgenic lines in the *eds1-2* and *pad4-1* mutant backgrounds and tested these for aphid fecundity. As before, aphid fecundity was higher on CathB6-GFP in wild-type (Col-0) plants than on GFP-only Col-0 lines (Fig. 4C). Aphid fecundity also increased on *pad4-1* GFP versus wild-type GFP plants and was slightly, but not significantly, higher on *eds1-2* GFP plants compared to the wild type (Fig. 4C). This aligns with reports showing a minor role for EDS1 and a larger effect of PAD4 on aphid fecundity (*38*, *39*). Aphid fecundity was not additionally improved by CathB6 overexpression in *eds1-2* or *pad4-1* mutants compared to that in the Col-0 background (Fig. 4C). These findings indicate that CathB6 modulates plant immunity in the EDS1-PAD4 pathway.

To more consistently assess whether CathB6 influences the expression of these EDS1-responsive genes, we used the *Pseudomonas fluorescens* Pf0-1 effector-to-host analyzer (EtHAn) system (*40*) to deliver either the ETI-inducing *Pseudomonas syringae* effector AvrRps4 or its non-ETI mutant AvrRps4^EEAA^ (*41*) into stable *A. thaliana* CathB6-overexpressing lines. AvrRps4, but not AvrRps4^EEAA^, induced the increased expression of 10 SA- and NHP-regulated genes in wild-type and GFP-expressing control plants, and the induction of all, except EDS1, was significantly suppressed in lines expressing CathB6, including EDS1 partners PAD4 and SAG101 (Fig. 4D). Similarly, the induction of key marker genes ICS1 and PR1 and a series of other SAR marker genes was profoundly reduced in CathB6 lines (Fig. 4D). These data were consistent across independent repeats (fig. S21). Therefore, CathB6 suppresses the expression of defense genes in the EDS1-mediated response pathway.

We also investigated whether CathB6 modulates local ETI by analyzing HR levels induced by AvrRps4 in *A. thaliana* Col-0 plants (*41*). Controls included *A. thaliana* Col-0 treated with AvrRps4^EEAA^ and *A. thaliana eds1-2* null mutants treated with AvrRps4, both of which should not induce HR responses (*41*, *42*). We observed a slight reduction in HR responses to AvrRps4 in CathB6-GFP lines (14 or 15 of 20 plants) and His-CathB6 lines (16 or 18 of 20 plants) compared to wild-type Col-0 (19 of 20 plants) and GFP plants (19 of 20 plants). However, the HR response was not completely suppressed (fig. S22). As expected, AvrRps4^EEAA^-treated wild-type plants (0 of 12 plants) and AvrRps4-treated *eds1-2* null mutants (0 of 18 plants) showed no HR response (fig. S22). Therefore, CathB6 does not appear to have a clear HR suppression activity.

### Acd28.9 counteracts CathB-mediated EDS1 sequestration to p-bodies

**Fig. 5.**
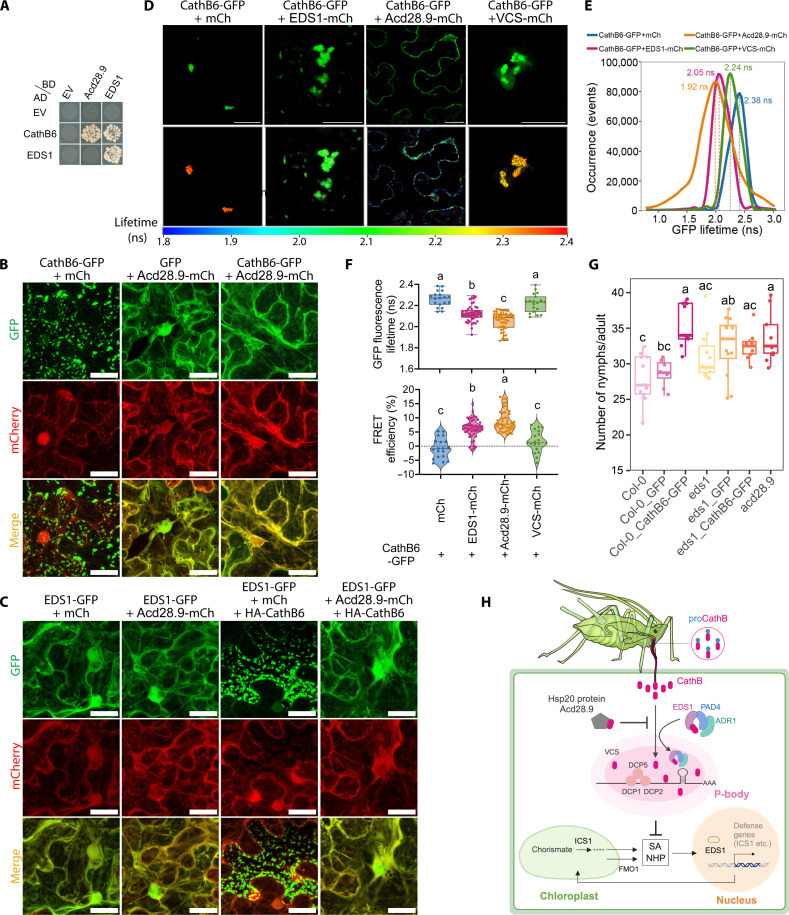
Acd28.9 counteracts CathB6 recruitment of EDS1 to p-bodies and contributes to plant resistance against aphids. (**A**) Y2H assays on selective SD-LWHA medium (yeast growth on SD-LW medium, fig. S14C). (**B** and **C**) Confocal images illustrating protein localization and colocalization: Acd28.9-mCherry coexpression depletes CathB6-GFP puncta in *N. benthamiana* (B), and EDS1-GFP forms puncta with HA-CathB6 and does not form puncta with Acd28.9-mCherry and HA-CathB6 (C). (**D** to **F**) FLIM-FRET analysis showing the reduced fluorescence lifetime of CathB6-GFP with EDS1 and Acd28.9, but not mCherry and VCS, with CathB6-GFP fluorescence and FLIM images (D), lifetime measurements of CathB6-GFP (E), and lifetime and FRET efficiency means ± SEM (*n* = 20 to 49, randomly selected leaf sections from three plants) (F). Scale bars, 30 μm. (**G**) *M. persicae* fecundity assays on *A. thaliana* lines (*n* = 8 to 12 replicates per line). Transgene expression levels in these lines are shown in fig. S4C. In [(F) and (G)], letters indicate differences determined by ANOVA (Tukey’s method, *P* < 0.05). (**H**) Model: CathB6 recruits EDS1-PAD4-ADR1 to p-bodies, suppressing defense, while Acd28.9 reduces CathB6 localization to p-bodies, counteracting this effect.

An Hsp20 family alpha crystallin domain protein 28.9 (Acd28.9) was also identified in CathB6 PL-MS (fig. S13). Given that another Acd Hsp20 family protein SLI1 is a resistance factor to *M. persicae* (*43*, *44*), we further investigated Acd28.9. We revealed that CathB6 and Acd28.9 interact in Y2H assays (Fig. 5A and figs. S14C and S23A), and Cath6 pulls down Acd28.9 from *N. benthamiana* leaves (fig. S23B). Acd28.9 did not interact with EDS1 in Y2H experiments (Fig. 5A). Acd28.9-mCherry locates throughout the cytoplasm and does not form obvious puncta (Fig. 5B and fig. S8E). Notably, CathB6 localization to puncta largely disappeared in the presence of Acd28.9 and resulted in CathB6 being widely distributed throughout the cytoplasm (Fig. 5B and fig. S7H), indicating that Acd28.9 may counteract CathB6 localization to p-bodies. Moreover, EDS1-GFP puncta that were observed in the presence of HA-CathB6 and free mCherry disappeared in the presence of Acd28.9-mCherry (Fig. 5C and fig. S7I), indicating that Acd28.9 also counteracts CathB6 relocation of EDS1 to p-bodies.

To investigate how CathB6 interacts with multiple proteins, a series of CathB6 fragments was tested for binding to EDS1 versus Acd28.9 in Y2H experiments. This showed that the CathB6^87–134^ region is primarily responsible for binding to EDS1 and the C-terminal CathB6^266–333^ region for interaction with Acd28.9 (fig. S24). A GFP fusion of a CathB6 mutant lacking the C-terminal region (CathB6^∆266–333^) localized to puncta and retained the ability to recruit EDS1 to p-bodies (fig. S25A). Whereas EDS1-GFP had a cytoplasmic distribution in the presence of Acd28.9-mCherry and full-length CathB6, EDS1-GFP located in puncta in the presence of HA-CathB6^∆266–333^ and Acd28.9-mCherry (fig. S25B). Notably, Acd28.9-mCherry also located to puncta in the presence of HA-CathB6^∆266–333^ (fig. S25B). Acd28.9 did not locate to p-bodies (Fig. 5B) and did not change the size and number of VCS-labeled p-bodies in the absence of CathB6 (fig. S26). These findings indicate that distinct regions of CathB6 mediate interactions with EDS1 and Acd28.9, with the C-terminal region specifically required for Acd28.9 binding and for Acd28.9-driven relocalization of CathB6 and EDS1 from p-bodies into the cytoplasm.

We conducted fluorescence lifetime imaging microscopy–based Förster resonance energy transfer (FLIM-FRET) experiments to obtain independent evidence for the direct interactions of CathB6 with EDS1 and Acd28.9 in plant cells. The lifetime of CathB6-GFP fluorescence was significantly reduced, and FRET efficiency significantly improved in the presence of EDS1-mCherry and Acd28.9-mCherry compared to mCherry (Fig. 5, D to F). No reduction of CathB6-GFP fluorescence lifetime was observed in the presence of VCS-mCherry versus mCherry (Fig. 5, D to F), suggesting that although VCS-mCherry was colocalized with CathB6-GFP, it does not directly interact with it. Thus, CathB6 directly interacts with EDS1 and Acd28.9, but not VCS, within plant cells.

Last, we investigated the impact of Acd28.9 on aphid performance. Aphid fecundity was significantly and consistently improved on *A. thaliana acd28.9* mutant plants compared to wild-type Col-0 (Fig. 5G and fig. S27), reaching levels similar to those observed on CathB-GFP and *eds1* CathB-GFP lines (Fig. 5G). This indicates that Acd28.9 contributes to *A. thaliana* resistance to *M. persicae*.

## DISCUSSION

We report here that CathB6 of the generalist peach-potato aphid *M. persicae* acts as an inhibitor of key plant immunity components. The data support a model in which CathB6 functions upstream of EDS1, recruiting EDS1, PAD4, and ADR1 to p-bodies, down-regulating SAR and promoting aphid fecundity (Fig. 5H). In addition, CathB6 interacts with a plant Hsp20 protein Acd28.9, which reduces CathB6 and EDS1 localization to p-bodies. Moreover, aphid fecundity increases on *acd28.9* mutant plants. Acd28.9 therefore counteracts the actions of CathB6 and mediates plant resistance to *M. persicae* (Fig. 5H).

This study shows that the CathB6 and CathB3 proteins in aphid OSs enhance aphid reproduction on *A. thaliana*, unlike CathB9 that is absent from OSs. CathB9-GFP has a diffuse cytoplasmic distribution similarly to GFP alone, does not enhance aphid fecundity, and does not interact with EDS1. Because the cysteine (C) in the catalytic triad is an aspartic acid (D) in CathB9, and it has been shown that mutating (*45*–*47*) or blocking (*48*) the catalytic cysteine abolishes protease catalytic activity in CathB cysteine proteases, we examined activities of CathB3 and CathB6 catalytic cysteine mutants, which were created by mutating the cysteine to aspartic acid (C to D) within the mature domain. We found that CathB3, CathB6, and their C-to-D mutants increase aphid fecundity; notably, CathB3 and CathB6^C114D^ also interact with EDS1 and colocalize with EDS1-mCherry in puncta. This indicates that aphid CathB proteins do not rely on peptidase activity mediated by the catalytic triad for in planta and aphid fecundity promotion activities. The aphid CathB effectors may function as inactive or “moonlighting” proteases, performing noncatalytic roles, a phenomenon commonly observed in proteins that resemble enzymes (*49*, *50*).

We found that CathB6 specifically associates with p-bodies, as indicated by their colocalization with the three p-body markers VCS, DCP1, and DCP5. P-bodies are membraneless compartments in the cytoplasm, primarily composed of mRNAs and proteins involved in translation repression and mRNA decay (*51*). In plants, the assembly and disassembly of p-bodies are regulated by light perception through the photoreceptor phytochrome, enabling the dynamic balance of mRNA storage, degradation, and translation (*25*, *52*). We observed that CathB6 increases p-body numbers and sizes and that multiple key defense genes are down-regulated in the presence of CathB. Given that p-bodies enlarge to serve as sites for mRNA storage and promote translational repression (*23*, *53*), these data suggest that CathB association with the p-bodies leads to repression of mRNA translation. Moreover, CathB6 recruits EDS1, PAD4, and ADR1 to p-bodies and the genes regulated by the EDS1-PAD4-ADR1 module were down-regulated. In contrast, CathB6 does not colocalize with SAG101 in the nucleus, and consistently, CathB6 exerts only a minor effect on HR suppression—an effect primarily mediated by the helper nucleotide-binding, leucine-rich repeat receptor (NLR) protein N requirement gene 1 (NRG1) via the EDS1-SAG101-NRG1 module (*28*). Therefore, CathB appears to target EDS1, PAD4, and ADR1 to p-bodies to down-regulate the EDS1-PAD4-ADR1–mediated immune pathway.

Our findings are consistent with previous studies showing that PAD4 is involved in aphid defense (*54*) and that aphid fecundity remains unchanged in *pad4 sag101* AtPAD4^R314A^ and *pad4 sag101* AtPAD4^K380A^ plants, where PAD4 EP cavity mutations disrupt EDS1-PAD4 signaling via ADR1 (*39*). Our data indicate that CathB6 functions upstream of EP domain signaling by recruiting the EDS1-PAD4-ADR1 complex to p-bodies, leading to gene expression down-regulation and mitigating the effects of the R314A and K389A mutations. These results also explain the modest increase in aphid fecundity on *eds1-2* plants, as *M. persicae* CathB6 likely suppresses EDS1 directly, while CathB6 indirectly influences PAD4 by down-regulating *pad4* expression. The direct inhibition of EDS1, compared to the delayed impact on PAD4, may account for the higher aphid fecundity observed on *pad4* and *adr1* mutants compared to *eds1* mutants.

In a previous study, Guo *et al.* (*12*) reported that a peptide corresponding to the propeptide region of a protein designated as CathB3, annotated as CathB20 in the *M. persicae* v2.1 genome (Fig. 1B and figs. S1 and S2), was detected in the OS of *M. persicae* feeding on *N. tabacum*. This propeptide was shown to activate plant immunity by interacting with the *N. tabacum* EDR1-like protein (*12*). In contrast, our study that used aphids reared on *A. thaliana* plants to collect the OS (*13*) identified a total of 14 peptides, all of which map to the mature domains of eight host-responsive CathB proteins, and these CathB proteins suppress *A. thaliana* plant defense responses. Notably, CathB expression appears to be host-specifically regulated, as it is consistently up-regulated in aphids feeding on *A. thaliana* and *Brassica rapa* (Brassicaceae) and down-regulated on *N. benthamiana* and potato (Solanaceae), with expression levels dynamically adjusting following host transfer (*10*, *11*). Therefore, *M. persicae* may modulate its OSs in a host-dependent manner, possibly because CathB exerts opposing effects on the immune responses of different plant species.

The CathB activity reported here, along with observations that *M. persicae* modulates CathB expression and OSs, is also intriguing in light of the fact that EDS1 plays a comparatively minor role in regulating plant defenses in *N. benthamiana* relatively to *A. thaliana* (*55*, *56*). The EDS1-PAD4 network is more dominant in Brassicaceae, possibly due to a greater diversity of TNLs [Toll/interleukin-1 receptor (TIR) domain-containing NLRs], while Solanaceae has a higher number of CNLs [Coiled-coil (CC) domain-containing NLRs] (*57*). Nonetheless, the EDS1-PAD4-ADR1 signaling node is broadly present across plants, including monocots, because of its role in both CNL function and basal immunity (*58*). Aphid CathB expression is up-regulated on monocots (fig. S1B), in agreement with a dominant role of the EDS1-PAD4-ADR1 module in regulating immunity in monocots (*28*). Whether CathB proteins suppress immunity in various plant species is still unknown, although CathB gene duplication, diversification, and positive selection in aphids (*59*) suggest adaptive immune modulation potential.

Acd28.9, an Hsp20 protein, counteracts the CathB6-mediated recruitment of EDS1 to p-bodies, enhancing *A. thaliana* resistance to *M. persicae*. Hsp20 family members are characterized by conserved C-terminal alpha crystallin domains and function as chaperones by preventing protein aggregation (*60*, *61*). Hsp20 proteins, found across all life domains, generally localize to the cytoplasm but may also occur in organelles and the nucleus, especially in plants (*61*). Here, we found that Acd28.9 localizes predominantly in the cytoplasm and can also locate in p-bodies. Plant Hsp20 proteins aid in salt and heat tolerance responses, and some, like RTM2 and SLI1, contribute to virus and insect resistance, respectively (*44*, *62*). Thus, at least two Hsp20 proteins contribute to aphid resistance, with Acd28.9 preventing accumulation of CathB6 in plant cell p-bodies.

Relying on plants for all life stages, aphids like *M. persicae* thrive across diverse hosts despite clonal reproduction, underscoring their ability to manipulate plant systems. In addition, aphid suppression of plant immunity creates an ideal pathway for viruses, facilitating infection and enhancing viral spread. Findings reported herein highlight an unexpected role of p-bodies in plant immunity and uncover a plant resistance mechanism to aphid infestation. This work shows that the functional study of aphid effectors, especially those in OSs, offers valuable insights into plant processes.

## MATERIALS AND METHODS

### Aphid colony maintenance

The *M. persicae* clone O (*63*) was reared on Chinese cabbage *B. rapa* (variety Hilton) or *A. thaliana* Col-0 and maintained in a growth chamber (20°C, 14-hour light/10-hour dark, 75% humidity) since 2010.

### Plant growth

Wild-type, mutant, or transgenic *A. thaliana* Col-0 plants used for seeds were grown under long day conditions (16-hour light/8-hour dark) at 20°C with a humidity of 80%. *A. thaliana* Col-0 plants used for fecundity assay were maintained under short day conditions (10-hour light/14-hour dark) at 22°C with a humidity of 70%. *N. benthamiana* plants were grown under a long day photoperiod (16-hour light/8-hour dark) at 22°C with a humidity of 80%.

### Phylogenetic analysis of *M. persicae* CathB protein

Genes encoding CathB proteins were extracted from the *M. persicae* clone O v2.0 genome database (*64*) and v2.1 annotation (*13*), identifying a total of 27 CathB proteins, one of which was truncated. A phylogenetic tree was generated using 26 full-length CathB sequences, with cathepsin L (CathL) and cathepsin O (CathO) as outgroups. Full-length protein sequence alignment was performed with the MUSCLE algorithm on the Phylogeny.fr web server (www.phylogeny.fr/index.cgi) (*65*). A maximum likelihood phylogenetic tree was constructed in FastTree using the Shimodaira-Hasegawa test with 1000 resamples (*66*). The resulting tree was visualized and edited with the Interactive Tree of Life (iTOL) online server (https://itol.embl.de) (*67*).

### CathB transcript level analysis across nine host plants

The updated CathB annotation was used to analyze the previously published RNA sequencing data of *M. persicae* clone O feeding on nine different plant species, which were reanalyzed by mapping reads to the v2.1 annotation (*13*) following similar methods (*10*). Transcript counts per million were generated, and differentially expressed CathB genes were identified by comparing transcript levels in aphids feeding on each host species to those in the original colonies on *B. rapa*. Differential expression criteria included a *P* value < 0.05, false discovery rate (FDR) < 5%, and log_2_(fold change) > 1. A heatmap was generated, scaled with *z*-scores (calculated by subtracting the mean expression and dividing by the standard deviation for each CathB gene across all hosts), and integrated with the phylogenetic tree using TBtools (*68*).

### Generation of CathB transgenic *A. thaliana* plants

The coding sequences corresponding to the mature domains of *M. persicae* CathB3 (Lys^61^-Asn^340^), CathB6 (Arg^61^-Asn^338^), and CathB9 (Glu^61^-Thr^338^) were amplified from *M. persicae* clone O cDNA and cloned into vector pBI121 for including an N-terminal His tag using Gibson assembly cloning methods (*69*) and to vector pB7FWG2.0 for including a C-terminal GFP tag using Gateway cloning methods (*70*). After verifying the inserts via sequencing, the constructs were transformed to *Agrobacterium tumefaciens* strain GV3101 and bacterial colonies were grown on LB solid medium containing rifampicin, gentamicin, and kanamycin (pBI121-CathB) or spectinomycin (pB7FWG2-CathB) at 28°C for 24 to 48 hours. Colonies were inoculated into liquid LB medium containing rifampicin, gentamicin, and kanamycin or spectinomycin and cultured overnight, followed by plasmid extraction to verify inserts using polymerase chain reaction (PCR) with gene-specific primers. Positive colonies were then cultured at 28°C in liquid culture and transformed to *A. thaliana* Col-0 plants using the floral dipping method (*71*). Transgenic seeds were harvested and selected on Murashige and Skoog medium containing kanamycin (50 μg/ml) for His-CathB transformants or phosphinothricin (20 μg/ml; BASTA) (Merck Life Sciences, 45520) for CathB-GFP transformants. Seeds from each generation were screened on Murashige and Skoog plates with the respective antibiotics to achieve a 3:1 survival-to-death segregation ratio. In T2- or T3-transformed *A. thaliana* plants, a 100% survival rate indicated homozygosity, and these plants were used for fecundity assays. Plasmids used for generating transgenic *A. thaliana* lines are listed in table S5. All *A. thaliana* lines used in the study are listed in table S6.

### *M. persicae* fecundity assay

Seeds from wild-type, mutant, or CathB transgenic *A. thaliana* lines were sown on Murashige and Skoog medium containing kanamycin or BASTA. Seedlings from the plates were transplanted to single pots of soil and grown in a controlled environmental room with a light period of 10-hour light/14-hour dark at 22°C. Before the assay, *M. persicae* clone O adult aphids were transferred from the *A. thaliana* stock colony to 3-week-old *A. thaliana* plants, followed by confining of the whole plant with a sealed cage. One day later, *M. persicae* nymphs produced by the adult were transferred to different lines of *A. thaliana* plants, with one nymph per plant. The nymphs matured into adults and began reproducing after 1 week, at which point their offspring were counted on days 7, 9, 11, 13, and 15 posttransfer. Results were calculated as the number of nymphs produced per adult. The assay was conducted two or three times, representing independent biological replicates, with 8 to 12 plants per line in each replicate. Plants used for the assay were randomly selected. Statistical significance was determined using a one-way analysis of variance (ANOVA) followed by Tukey’s multiple comparison test, conducted with GraphPad Prism 10 software. Differences were considered statistically significant with a *P* value <0.05. Box plots were generated using R (version 4.3.2).

### Quantification of CathB transgene expression in plants by qRT-PCR

CathB transgenic plants in Col-0, *eds1-2*, and *pad4-1* backgrounds used for *M. persicae* fecundity assay were quantified for CathB expression using quantitative reverse transcription PCR (qRT-PCR). Briefly, total RNA was isolated from a randomly selected plant using the RNeasy plant mini kit (QIAGEN, 74904) and subsequent deoxyribonuclease (DNase) treatment using ribonuclease-free DNase I (Thermo Fisher Scientific, EN0521). cDNA was synthesized from 1 μg of total RNA with the RevertAid First Strand cDNA synthesis Kit (Thermo Fisher Scientific, K1622). The qRT-PCR reactions were performed on a CFX96 Touch Real-Time PCR detection system (Bio-Rad) in triplicate. Each reaction was conducted in a 20-μl system containing 10 μl of Maxima SYBR Green master mix (Thermo Fisher Scientific, K0221), 0.5 μl of each primer (10 μM), 1 μl of sample cDNA, and 8 μl of nuclease-free water (Thermo Fisher Scientific, R0581). The cycle conditions were as follows: 95°C for 10 min, 40 cycles at 95°C for 15 s, and 60°C for 60 s. All data were normalized to EF1α (accession number AT5G60390). Relative quantification was calculated using a comparative method as 2^−△Ct^ (*72*). Box plots were generated using GraphPad Prism 10. Primers used for qRT-PCR are shown in table S7.

### Subcellular localization

#### *Transient expression in* N. benthamiana *leaves and* A. thaliana *protoplasts*

Plasmids used for transient expression in *N. benthamiana* leaves and transformation of *A. thaliana* protoplasts were generated by amplifying target sequences from cDNA and cloning these into pB7FWG2.0 for adding GFP, pB7RWG2.0 for RFP, or pB7WG2.0 for mCherry tags, using Gibson assembly and Gateway cloning, and are listed in table S5.

For transient expression *N. benthamiana* leaves, the constructs were transformed into the *A. tumefaciens* strain GV3101, plated on LB solid medium with appropriate antibiotics, and grown at 28°C for 24 to 48 hours. Colonies were picked and verified by PCR with gene-specific primers using plasmid DNA from overnight liquid cultures. Positive colonies were cultured overnight at 28°C, harvested, and resuspended in infiltration buffer (10 mM MgCl_2_ and 10 mM MES, pH 5.6) with 100 μM acetosyringone. For infiltration of leaves, GFP- or YFP (yellow fluorescent protein)–tagged constructs were combined with RFP- or mCherry-tagged constructs and pCB301-P19 at an optical density at 600 nm of 0.3 for each and infiltrated into the abaxial surface of 4-week-old *N. benthamiana* leaves using a 1-ml needleless syringe. Infiltrations were performed on randomly selected leaves of two different plants. Leaf samples were collected for imaging at 48 to 72 hours postinfiltration (hpi). Live leaf sections were mounted in water for microscopy.

For *A. thaliana* protoplast transformation, plasmids were extracted and purified from *Escherichia coli* using the QIAGEN Plasmid Midi Kit (QIAGEN, 12143). *A. thaliana* mesophyll protoplasts were isolated and transformed as reported (*73*). Briefly, protoplasts were isolated from leaves of 3-week-old *A. thaliana* plants, which were grown at 22°C under short day (10-hour light/14-hour dark) conditions with a humidity of 70%. Transformation was conducted by cotransforming 300 μl of fresh protoplast solution (400,000/ml) and 12 μg of high-quality plasmids of each construct using the PEG-calcium method. Transformed protoplasts were kept in the dark for 16 hours at 22°C. Transformed protoplasts were observed by confocal microscopy the next day.

#### Colocalization analysis using confocal microscopy

Colocalization analyses were conducted with a Leica TCS SP8X upright confocal laser scanning microscope using either a 20×/0.75 dry or 63×/1.20 water immersion objective (HC PL APO CS). Sequential, unidirectional scans were carried out using the following laser lines: 405 nm, 488 nm, 514 nm (from a 65-mW argon ion laser), and 580 nm (pulsed SuperK EXTREME supercontinuum white light laser, 470 to 670 nm, 1.5 mW per line) to excite BFP (blue fluorescent protein)/DAPI (4′,6-diamidino-2-phenylindole), GFP, YFP, and RFP/mCherry, respectively. Fluorescence emissions were collected at 420 to 450, 505 to 540, 529 to 550, and 595 to 620 nm. Images were acquired on hybrid detectors at a laser power <5%, with line averaging 2 and a pinhole of 1 Airy unit. Images were generated with varying gains and *z*-steps. The pixel size was set at 0.18 μm by 0.18 μm and pixel dwell time at 600 ns.

#### Time-lapse recording using confocal microscopy

The time-lapse experiment was performed with a Leica Stellaris 8 FALCON upright confocal microscope using a 63×/1.20 water immersion objective (HC PL APO CS2) and xyzt scan mode. Bidirectional scans were carried out using a 20-mW 488-nm laser diode to excite GFP, and fluorescence emissions were collected at 505 to 540 nm. Images were acquired on hybrid detector S at a laser power < 5%. *z*-Stacks through the entire puncta were taken at minimum intervals. The pixel size was set at 0.18 μm by 0.18 μm and pixel dwell time at 1.0375 μs.

Image processing and statistical analyses (e.g., punctum size, number, and intensity profiles) were performed with ImageJ (FIJI). Graphs were generated using GraphPad Prism 10 software. Significance was assessed using two-tailed Student’s *t* test. Differences were considered statistically significant with a *P* value <0.05. Each experiment was independently repeated at least three times.

#### Protein presence analyses via Western blotting

Following confocal microscopy, leaf samples were extracted with 4× NuPAGE LDS Sample Buffer (Invitrogen, NP0007) supplemented with 10 mM dithiothreitol (DTT). Proteins were separated on 12% NuPAGE bis-tris gels (Invitrogen, NP0343BOX) and transferred to 0.45-μm polyvinylidene difluoride membranes (Thermo Fisher Scientific, 88518). Membranes were then probed with the following antibodies: anti-GFP (Santa Cruz, sc-9996), anti-mCherry (Abcam, AB167453), anti-HA (BioLegend, 901502), anti-Flag (Merck Life Sciences, M8823), and anti-EDS1 (Agrisera, AS132751).

### Fluorescence recovery after photobleaching

CathB6-GFP constructs were transformed into the *A. tumefaciens* strain GV3101 and infiltrated into *N. benthamiana* leaves as described. After 48 hours, leaf sections from infiltrated plants were mounted in water and observed with a Leica Stellaris 8 FALCON upright confocal microscope. Using a 63×/1.20 water immersion objective, a region within a CathB6-GFP punctum was photobleached with 50% laser intensity at 488 nm for three iterations. Recovery was recorded every 2 s for a total of 120 s postbleaching. Recovery analysis was performed with FIJI, with the fluorescence intensity at each time point normalized using the FRAP (fluorescence recovery after photobleaching) profiler version 2 plug-in. The recovery curve was then analyzed and plotted using GraphPad Prism 10 software.

### Yeast two-hybrid assay

Coding sequences corresponding to the mature domains of *M. persicae* CathB3 (Lys^61^-Asn^340^), CathB6 (Arg^61^-Asn^338^), CathB9 (Glu^61^-Thr^338^), and the CathB6^C114D^ mutant were amplified and cloned into the Gateway vector pDEST-GBKT7 (BD). Full-length sequences of *A. thaliana* EDS1, PAD4, SAG101, and Acd28.9 were amplified and cloned into pDEST-GADT7 (AD). To test interactions between CathB6 and various EDS1 truncations, different fragments of EDS1—including full-length EDS1, the lipase-like domain (EDS1^1–384^), the EP domain (EDS1^385–623^), and additional truncations of the EP domain (EDS1^385–444^, EDS1^445–504^, EDS1^505–564^, EDS1^565–623^, and EDS1^405–554^)—were cloned into pDEST-GBKT7 (BD), with the CathB6 mature domain cloned into pDEST-GADT7 (AD) using Gateway cloning methods. All plasmids generated are listed in table S5.

Constructs for protein-protein interaction testing were cotransformed into the *Saccharomyces cerevisiae* strain AH109. Empty pDEST-GBKT7 and pDEST-GADT7 vectors served as negative controls. Transformants were first assessed on solid double dropout medium lacking leucine and tryptophan (SD-LW) to confirm the presence of both AD and BD constructs. Interactions between AD and BD fusion proteins were screened on triple dropout medium lacking leucine, tryptophan, and histidine (SD-LWH) supplemented with 5 mM 3-amino-1,2,4-triazole and on quadruple dropout medium lacking leucine, tryptophan, histidine, and adenine (SD-LWHA). Yeast plates were incubated at 28°C for 5 days before imaging.

### Coimmunoprecipitation in *N. benthamiana*

The coding sequences of the CathB6 mature domain (Arg^61^-Asn^338^) and its corresponding catalytic cysteine mutant were constructed to pB7FWG2.0 for CathB6-GFP and CathB6^C114D^-GFP. Full-length coding sequence *A. thaliana* EDS1 or Acd28.9 was amplified and tagged with 3× HA (hemagglutinin) at the N terminus and then ligated into the Gateway vector pB7WG2.0. Constructs were separately transformed to the *A. tumefaciens* strain GV3101 and co-infiltrated into 4-week-old *N. benthamiana* leaves using the same methods above. Infiltrated leaves were harvested between 48 and 72 hpi. Total proteins were extracted with extraction buffer [150 mM tris-HCl (pH 7.5), 150 mM NaCl, 10 mM EDTA, 10% glycerol, 20 μM NaF, 10 mM DTT, 0.5% (w/v) polyvinylpolypyrrolidone (PVPP), 1% protease inhibitor cocktail (Sigma-Aldrich), and 0.2% Igepal]. GFPtrap beads (Chromotech, gtma-20) were then added into the extracts and incubated on a rotor wheel at 4°C overnight. The next day, beads were washed six times with washing buffer [10 mM tris-HCl (pH 7.5), 150 mM NaCl, 0.5 mM EDTA, and 0.2% Igepal]. After washing, proteins were eluted from the beads using 4× NuPAGE LDS Sample Buffer (Invitrogen, NP0007) with 10 mM DTT. Proteins were detected via Western blotting as described above. The membranes were probed with anti-GFP (Santa Cruz, sc-9996) and anti-HA (BioLegend, 901502) antibodies.

### Protein expression in Sf9 cells using the baculovirus expression system

The coding sequences of the CathB6 mature domain (Arg^61^-Asn^338^) and full-length *A. thaliana* EDS1 were individually tagged with GFP and HA and then cloned into the pFastBac HTB vector using Gibson assembly. After sequencing confirmation, the constructs were transformed into *E. coli* DH10Bac cells, which contain a baculovirus shuttle vector—a bacterial artificial chromosome carrying the full genome of *Autographa californica* multiple-nucleocapsid nucleopolyhedrosis virus. All generated plasmids are listed in table S5.

Following transformation, cells were plated on LB agar with kanamycin (50 μg/ml), tetracycline (10 μg/ml), gentamicin (7 μg/ml), isopropyl-β-d-thiogalactopyranoside (40 μg/ml), and X-galactosidase (100 μg/ml). White colonies were selected, streaked on fresh plates, and screened through four cycles. Bacmids were extracted from positive colonies using an SDS lysis, chloroform extraction, and ethanol precipitation method (*74*). After verifying recombinant bacmids by PCR, they were transfected into Sf9 cells (Thermo Fisher Scientific, 11496015)—a cell line derived from the lepidopteran insect *Spodoptera frugiperda*—using Lipofectamine 2000 (Thermo Fisher Scientific, 11668027).

Five to six days posttransfection, when cells exhibited signs of infection—such as increased size, granularity, cessation of growth, detachment, and lysis—the cell medium containing viral particles was collected as the P0 viral stock. This stock was used to infect fresh Sf9 cells, generating a P1 viral stock. The virus was further amplified through two additional cycles, producing a P3 viral stock for optimal protein expression.

### In vitro coimmunoprecipitation following expression in Sf9 cells

P3 viral stocks of CathB6-GFP and HA-EDS1 were used to infect fresh Sf9 cells for protein expression. Cells were harvested 48 to 72 hours postinfection, and total protein was extracted using a lysis buffer [25 mM tris-HCl (pH 7.4), 150 mM NaCl, 1% NP-40, 1 mM EDTA, and 5% glycerol] with or without 1% protease inhibitor cocktail (Merck Life Sciences, P9599). Anti-HA magnetic beads (Thermo Fisher Scientific, 88836) were added to the extracts, followed by overnight incubation on a rotating wheel at 4°C. The following day, the beads were washed six times with washing buffer (tris-buffered saline with 0.05% Tween 20). After washing, proteins were eluted from the beads using 4× NuPAGE LDS Sample Buffer (Invitrogen, NP0007) supplemented with 10 mM DTT. Proteins were detected via Western blotting as described above. The membranes were probed with anti-GFP (Santa Cruz, sc-9996), anti-HA (BioLegend, 901502), and anti-EDS1 (Agrisera, AS132751) antibodies.

### Expression analysis of EDS1-regulated genes by qRT-PCR

#### Plants treated with aphids

The *A. thaliana* Col-0 wild-type, GFP, and CathB6-GFP transgenic *A. thaliana* plants were grown in a controlled environmental room with a light period of 10-hour light/14-hour dark at 22°C as previously mentioned. Two 3-week-old plants from each line were randomly selected and confined in sealed cages. Around 500 *M. persicae* (including both adults and nymphs) were inoculated to the plants and kept for 24 hours. The next day, all aphids were removed and plant leaves were harvested for RNA extraction. Total RNA extraction, DNase treatment, cDNA synthesis, and qRT-PCR were conducted using the same method as described above. Primers used for qRT-PCR are shown in table S7. All gene expression data were normalized to EF1α (accession number AT5G60390), and relative quantification was calculated using a comparative method as 2^−ΔΔCt^ (*72*). Column charts were generated using GraphPad Prism 10.

#### Plants treated with AvrRps4/AvrRps4^EEAA^ effectors

The *P. fluorescens* Pf0-1(T3SS) EtHAn system (*40*) carrying AvrRps4 or AvrRps4^EEAA^ strains (*41*) was grown on King’s B agar plate supplemented with chloramphenicol (30 μg/ml) and genetamycin (50 μg/ml) at 28°C overnight. Bacteria were harvested, resuspended in infiltration buffer (100 mM MES and 100 mM MgCl_2_), and adjusted to an optical density at 600 nm of 0.2. The bacterial suspensions were infiltrated into the abaxial surface of 5-week-old *A. thaliana* plants using a 1-ml needleless syringe. Three plants from each *A. thaliana* line were randomly selected, with one leaf per plant infiltrated with Pf0-1 AvrRps4 and another with AvrRps4^EEAA^. Mock samples were infiltrated with buffer only. Leaves were harvested 8 hpi, and total RNA extraction, DNase treatment, cDNA synthesis, and qRT-PCR were conducted using the same method as described above. Primers used for qRT-PCR are shown in table S7. All gene expression data were normalized to EF1α (accession number AT5G60390), and relative expression levels were calculated using the 2^−ΔΔCt^ method (*72*), with mock samples as the reference. Grouped column charts were generated with GraphPad Prism 10 software, with data represented as the means ± SEM. Statistical significance was assessed using two-way ANOVA (plant lines and treatments) followed by Tukey’s multiple comparison test (GraphPad Prism 10). Differences were considered statistically significant with a *P* value < 0.05.

### HR assay in *Arabidopsis*

The *P. fluorescens* Pf0-1EtHAn system carrying either the AvrRps4 or AvrRps4^EEAA^ strain was prepared and infiltrated into 5-week-old *A. thaliana* plants as described. Infiltrations were conducted on randomly selected leaves, with each plant receiving one infiltration of Pf0-1 AvrRps4 on one leaf and AvrRps4^EEAA^ on another. A total of 12 to 20 plants was treated. Plants were then observed and assessed for cell death as an indicator of HR 24 hpi.

### FLIM-FRET analysis

Constructs expressing genes of interest (CathB6-GFP, mCherry, EDS1-mCherry, Acd28.9-mCherry, and VCS-mCherry) were transformed into the *A. tumefaciens* strain GV3101 and co-infiltrated into 4-week-old *N. benthamiana* leaves as described. Infiltrations were performed on randomly selected leaves of two different plants. Leaf samples were collected for imaging after 48 hpi. Live leaf sections were mounted in water and examined using a Leica Stellaris 8 FALCON scanning confocal microscope with a 63×/1.20 water immersion objective. FLIM experiments were conducted in time-correlated single-photon counting mode with highly sensitive photon detectors (HyD X). GFP was excited using a pulsed white light laser at 488 nm, with emission collected between 505 and 520 nm. The laser power was maintained below 1% to avoid sample bleaching, with a frequency set to 80 MHz.

The instrument response function was calibrated using potassium iodide and erythrosine B, as previously described (*75*). FLIM datasets were recorded using Leica LAS X software with the FLIM Wizard, with each image acquisition continued until a minimum of 5000 photon counts per pixel was reached. Images were acquired at a resolution of 128 pixels by 128 pixels with a pixel dwell time of 19 μs, and the laser power was adjusted to a maximum count rate of 2000 kcounts per second. Each acquisition was stopped after 40 frames.

Data analysis involved measuring the excited-state lifetime values of regions of interest randomly selected from the observation field, with calculations performed using LAS X software. Lifetime values were obtained by reconvolution fitting, selecting a two-exponential fit, and ensuring a χ^2^ value between 0.90 and 1.20. The amplitude-weighted mean lifetime (τ) was used for comparison, and FRET efficiency was calculated by comparing the lifetime of CathB6-GFP coexpressed with EDS1-mCherry, Acd28.9-mCherry, and VCS-mCherry (τ_x_) to that of CathB6-GFP with mCherry alone (τ_0_) using the formula FRET efficiency = 1 − (τ_x_/τ_0_).

Box plots and violin plots were generated using GraphPad Prism 10, and statistical significance was assessed by one-way ANOVA followed by Tukey’s multiple comparison test with GraphPad Prism 10 software. Differences were considered statistically significant with a *P* value < 0.05.

## Supplementary Materials

### The PDF file includes:

Figs. S1 to S27 Tables S1 to S7 Uncropped Western blots for fig. S7 Legend for movie S1 References
