# Supplementary material for: Aphid effectors suppress plant immunity via recruiting defense proteins to processing bodies
Source: Sci Adv. 2025 Jul 16;11(29):eadv1447. doi: 10.1126/sciadv.adv1447 (PMC12266115; doi:10.1126/sciadv.adv1447)
Supplement: Supplementary file 1 — Figs. S1 to S27 Tables S1 to S7 Uncropped Western blots for fig. S7 Legend for movie S1 References [file sciadv.adv1447_sm.pdf]

Science Advances
AAAS

Supplementary Materials for

Aphid effectors suppress plant immunity via recruiting defense proteins to processing bodies

Qun Liu *et al.*

Corresponding author: Saskia A. Hogenhout, saskia.hogenhout@jic.ac.uk

Sci. Adv. **11**, eadv1447 (2025)
DOI: 10.1126/sciadv.adv1447

The PDF file includes:

Figs. S1 to S27
Tables S1 to S7
Uncropped Western blots for fig. S7
Legend for movie S1
References

Other Supplementary Material for this manuscript includes the following:

Movie S1

A

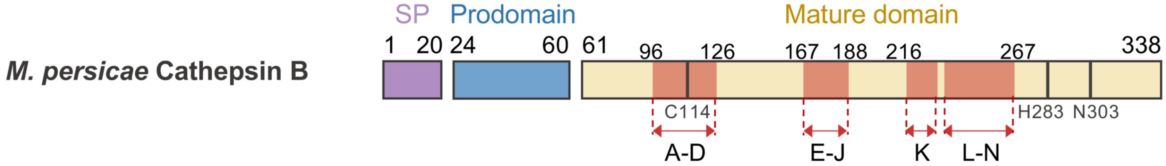

No.	Peptides	Targeted CathB	No.	Peptides	Targeted CathB
A	WVYCDTIGR	CathB14	H	KGLVTGGDYK	CathB6, 12
B	VRDQGNCGSCWAVATSSAFADR	CathB6, 12, 14	I	GLVTGGDYK	CathB6, 12
C	DQGNCGSCWAVATSSAFADR	CathB6, 12, 14, 17, 18	J	SGEGCEPYR	CathB3, 6, 12, 17, 18, 19, 20
D	DQGNCGSCWALATSSAFADR	CathB19, 20	K	MCYGDQDLDFDEDHR	CathB6, 12
E	FSTHGLVTGGDYNSGEGCEPYR	CathB14	L	DYYYLTYGSIQK	CathB3, 6, 12, 14, 17, 18
F	SHGLVTGGDYK	CathB19, 20	M	DSYYLTYGSIQK	CathB19,20
G	HGLVTGGDYK	CathB3, 17, 18	N	DVMTYGPIEASFDVYDDFPSYK	CathB3, 6, 12, 17, 18, 19

B

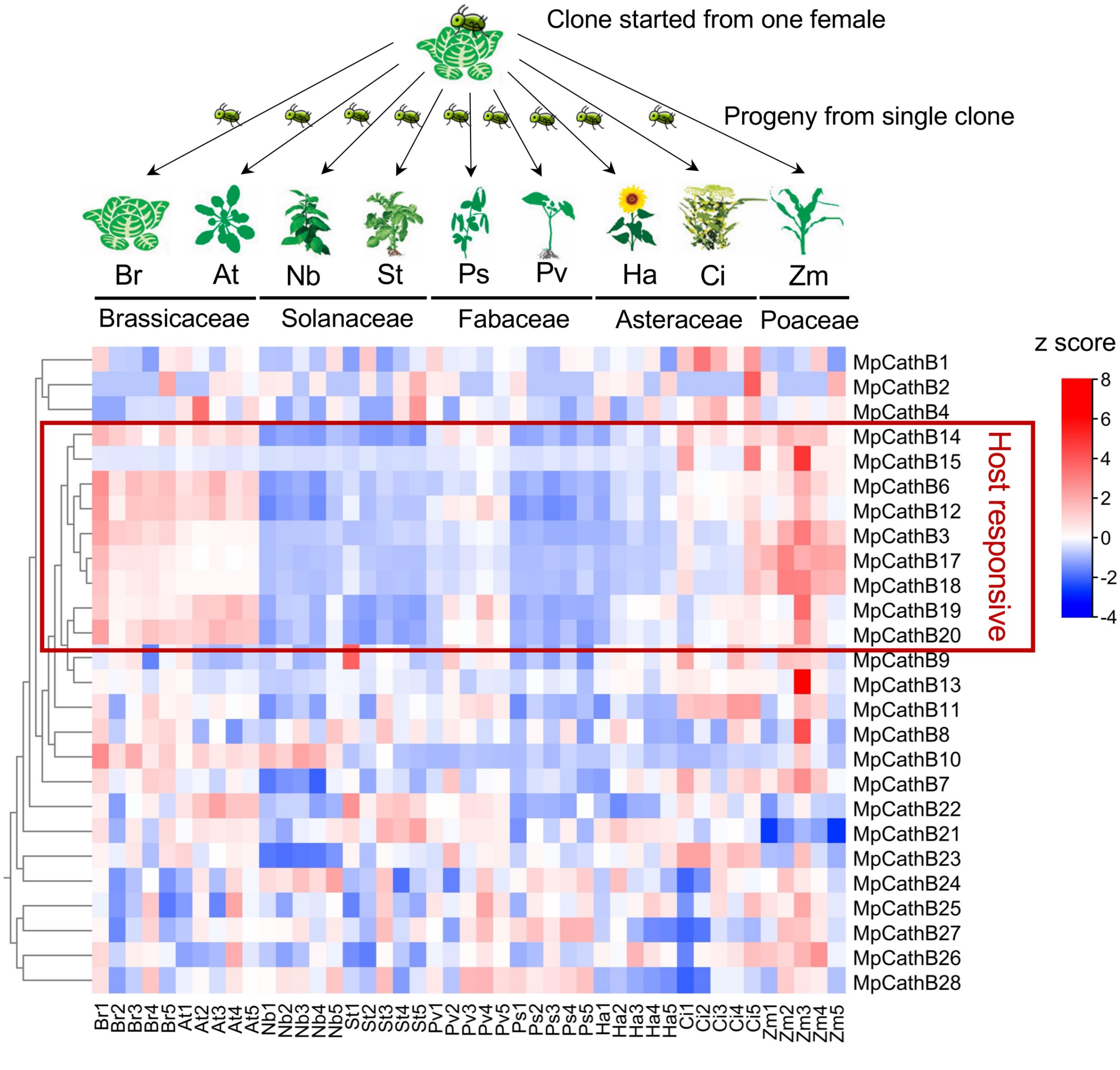

Fig. S1. *M. persicae* CathB proteins present in oral secretion (OS) are host-responsive. (A) Location of CathB peptides detected in *M. persicae* oral secretions (OS). The peptides matched the CathB mature domain. The signal peptide (SP) and prodomain are also indicated. Detected

peptides by MS are marked in light red. The catalytic triad residues (Cys114, His283 and Asn303) are marked. Table shows the specific peptides that were detected and which CathB they are likely derived from. The A to N numbering match those in the schematic overview above. Full proteome dataset of *M. persicae* OS is shown in (*13*). (**B**) *M. persicae* CathB differentially express in the aphids on different host plants. (Top) Schematic overview of *M. persicae* clonal populations transferred to 9 plant species from 5 families, as shown. Br, *Brassica rapa*; At, *A. thaliana*; Nb, *Nicotiana benthamiana*; St, *Solanum tuberosum*; Ps, *Pisum sativum*; Pv, *Phaseolus vulgaris* (Pv); Ha, *Helianthus annuus*; Ci, *Chrysanthemum indicum*; Zm, *Zea mays* (Zm). (Bottom) Heatmap of CathB expression values (TPM) of *M. persicae* on nine plant species at five biological replicates per host plant (as indicated below the heatmap). CathB marked in a red rectangle were found to have synchronized up- or down regulation depending on the plant species the aphids were reared on. Gene IDs for MpCathBs are provided in Table S1.

```
                1                                                                      70
MpCathB3        MARVLMLLSV IFVSVYVTEQ AYFLQKDFID NINEQATTWK AGVNFDPNTP KEHFLKLLGS KGVQTPNKNN
MpCathB6        MARVLMLLSV IFVSVYLTEQ AYFLEKDFID NINAQATTWK AGVNFDPKTS KEHIMKLLGS RGVQIPNKNN
MpCathB12       MARVLMLLSV IFVSVYMTEQ AYFLEKDFID NINEQATTWK AGVNFDPKTS KEHIMKLLGS RGVQIPNKNN
MpCathB14       MARVFMLLSV IFVSVYATEQ AYFLQEDFIN NINEQATTWK AGMNFDPNTP HDDIIKLLGS RGVQNLDKVN
MpCathB15       MAGVLMLLSV IFISVYVTEQ AYFLQEDFIN NINEQATTWK AGVNFDPNTP HDHLIRLLGS KGVQIPDKVN
MpCathB17       MARVLMLLSV IFVSVYVTEQ AYFLQKDFID NINEQATTWK AGVNFDPSTP KEHFLKLLGS KGVQTPNKNN
MpCathB18       MARVLMLLSV IFVSVYVTEQ AYFLQKDFID NINEQATTWK AGVNFDPSTP KEHFLKLLGS KGVQTPNKNN
MpCathB19       ---------- ------MTEQ AHFLEKDYID SINAQATTWK AGVNFPPSTP KEAILRLLGS RGVQIPNKAN
MpCathB20       MARVLILLSV ILFSVYMTEQ AYFLEKDYID SINAQATTWK AGVNFPPSTP KEAILRLLGS RGVQIPNKAN
                ::.:.::::: ::.:::.*** *:**::*:*: :**.****** **:**:*.*: ::..::**** :***.::*.*

                71                                                                    140
MpCathB3        IKMYKTDDAA YDNLFGRIPR HFDARRKWKK CHTIGAVRDQ GNCGSCWAVA TSSAFADRLC VATNADFNEL
MpCathB6        MNLYKSEDAD YDNTY--IPR FFDARRKWRH CSTIGRVRDQ GNCGSCWAVA TSSAFADRLC VATNADFNEL
MpCathB12       MNLYKSEDAD YNNTY--IPR FFDARRKWRH CKTIGRVRDQ GNCGSCWAVA TSSAFADRLC VATNADFNEL
MpCathB14       HKLYKTHDEA YDNLFGRIPK HFDARNKWVY CDTIGRVRDQ GNCGSCWAVA TSSAFADRLC VATTGDFNEL
MpCathB15       SKLYKTHDEA YDNFFGRIPK HFDARRKWRH CDTIGKVLDQ GNCGSDWGVA TSSAFADRLC IATNGDFNEL
MpCathB17       IKMYKSDDAA YDNLFGRIPR HFDARRKWKK CHTIGAVRDQ GNCGSCWAVA TSSAFADRLC VATNADFNEL
MpCathB18       IKMYKSDDAA YDNLFGRIPR HFDARRKWKK CHTIGAVRDQ GNCGSCWAVA TSSAFADRLC VATNADFNEL
MpCathB19       YKMYKSRDSN YDNLFGRIPK KFDARKKWRK CKTIGAVRDQ GNCGSCWALA TSSAFADRLC VATDADFNEF
MpCathB20       YKMYKSRDSN YDNLFGRIPK KFDARKKWRK CKTIGAVRDQ GNCGSCWALA TSSAFADRLC VATDADFNEF
                 ::**:.*.. *:*.:::**: .****:**.. *.***.*:** *****:*::* ********** :**::****:

                141                                                                   210
MpCathB3        LSAEEITFCC HSCGFGCDGG YPIKAWERFS KHGLVTGGDY KSGEGCEPYR VPPCPYDEQG NNTCAGKPME
MpCathB6        LSAEEITFCC HTCGFGCNGG YPIKAWKRFS KKGLVTGGDY KSGEGCEPYR VPPCPNDDQG NNTCAGKPME
MpCathB12       LSAEEITFCC HTCGFGCNGG YPIKAWKRFS KKGLVTGGDY KSGEGCEPYR VPPCPNDDQG NNTCAGKPME
MpCathB14       LSAEEITFCC HTCGFGCHGG YPIKAWKRFS THGLVTGGDY NSGEGCEPYR VPPS-ND--G NSSSSDQPLA
MpCathB15       LSAEEITFCC HTCGFGCHGG YPIKAWKRFS THGLVTGGEY NSGEGCQPYK FPPT-NDDEG NSTSSKQPSV
MpCathB17       LSAEEITFCC HSCGFGCDGG YPIKAWERFS KHGLVTGGDY KSGEGCEPYR VPPCPYDEQG NNTCAGKPME
MpCathB18       LSAEEITFCC HSCGFGCDGG YPIKAWERFS KHGLVTGGDY KSGEGCEPYR VPPCPYDEQG NNTCAGKPME
MpCathB19       LSPEELTFCC HTCGNGCNGG HSIKAWERFK SHGLVTGGDY KSGEGCEPYR VPPCRHHAEG NNSCSDKPME
MpCathB20       LSPEELTFCC HTCGYGCNGG YPIKAWERFK SHGLVTGGDY KSGEGCEPYR VPPCRHHAEG NNSCSDKPME
                **:**:**** *:**:**.** ::****:**: .:******:* :*****:**: :**: .:..* *::::.:*:.

                211                                                                   280
MpCathB3        SNHRCTRMCY GNQDLDFDQD HRYTRDYYYL TYGSIQKDVM TYGPIEASFD VYDDFPSYKS GVYVKSENAT
MpCathB6        SNHRCTRMCY GDQDLDFDED HRYTRDYYYL TYGSIQKDVM TYGPIEASFD VYDDFPSYKS GVYVKSENAS
MpCathB12       SNHRCTRMCY GDQDLDFDED HRYTRDYYYL TYGSIQKDVM TYGPIEASFD VYDDFPSYKS GVYVKSENAS
MpCathB14       INHICRRHCY GNQSIDFNDD HRYTRDYYYL TYGSIQKDVL TYGPIEASFD VYDDFPSYKS GVYVKSDNAS
MpCathB15       WDLRCTRSCY GNQSIDFYDD HRYTRDYYYL TYGSIQKDVM AYGPIEASFD VYDDFFVYKS GVYVRSENAT
MpCathB17       SNHRCTRMCY GNQDLDFDQD HRYTRDYYYL TYGSIQKDVM TYGPIEASFD VYDDFPSYKS GVYVKSENAT
MpCathB18       SNHRCTRMCY GNQDLDFDQD HRYTRDYYYL TYGSIQKDVM TYGPIEASFD VYDDFPSYKS GVYVKSENAT
MpCathB19       KNHRCTRMCY GDQDLDFDDD HRYTRDSYYL TYGSIQKDVM TYGPIEASFD VYDDFPSYKS GVYIRSDNAS
MpCathB20       KNHRCTRMCY GDQDLDFDDD HRYTRDSYYL TYGSIQKDVM NYGPIEASFD VYDDFPSYKS GVYIRSDNAS
                 :::*:*.** *:*::**::* ******:*** *********: :********* *****::*** ***::*:**:
```

```
                281                                                         340
MpCathB3        YLGGHAAKLI GWGEEYGVPY WLMVNSWNAD WGDNGLFKIQ RGTNECGIDN STTAGVPVTN
MpCathB6        YLGGHAVKLI GWGEEYGVPY WLMVNSWNED WGDNGFFKIQ RGTNECGVDN STTAGVPVTN
MpCathB12       YLGGHAVKLI GWGEEYGVPY WLMVNSWNAD WGDNGLFKIQ RGTNECGVDN STTAGVPVTN
MpCathB14       YLGGHAVKLI GWGEEDGTPY WLMVNSWNTQ WGDNGFFKIR RGTNECGVDN STTAGVPVTN
MpCathB15       YVGGHAVKLI GWGEENGTPY WLMVNSWNED WGDNGLFKIQ RGTNECGVDN STTAGVPVTN
MpCathB17       YLGGHAAKLI GWGEEYGVPY WLMVNSWNAD WGDNGLFKIQ RGTNECGIDN STTAGVPVTN
MpCathB18       YLGGHAAKLI GWGEEYGVPY WLMVNSWNAD WGDNGLFKIQ RGTNECGIDN STTAGVPVTN
MpCathB19       YLGGHAVKLI GWGEESGVPY WLMVNSWNTD WGDKGLFKIQ RGTNECGVDN STTAGVPVTN
MpCathB20       YLGGHAVKLI GWGEESGVPY WLMVNSWNTD WGDKGLFKIQ RGTNECGVDN STTAGVPVTN
                *:****:*** *****.*:** ********.: ***:*:***: *******:** **********
```

Fig. S2. Multiple sequence alignment of host-responsive *M. persicae* CathB proteins generated using MUSCLE. Fully conserved residues across all sequences are highlighted with a grey background and marked by asterisks (*). Strongly similar residues are indicated by colons (:) and weakly similar residues by periods (.). Corresponding gene IDs are listed in Table S1. MpCathB20 (MYZPE13164_O_EIv2.1_0159280) was previously designated as "CathB3" (A9JSH5) in Guo et al. (2020) (*12*), based on a different genome version.

```
            1   Signal Peptide                   30        Prodomain               60
MpCathB3    MARVLMLLSV IFVSVYVTEQ AYFLQKDFID NINEQATTWK AGVNFDPNTP KEHFLKLLGS
MpCathB6    MARVLMLLSV IFVSVYLTEQ AYFLEKDFID NINAQATTWK AGVNFDPKTS KEHIMKLLGS
MpCathB9    MTRVLIIVSV ILINAYLTKQ AYFLEKDFID NINKQATTWK AGVNFDPNTP KEHILRLLGS

            61                            90                               * 120
MpCathB3    KGVQTPNKNN IKMYKTDDAA YDNLFGRIPR HFDARRKWKK CHTIGAVRDQ GNCGSCWAVA
MpCathB6    RGVQIPNKNN MNLYKSEDAD YDNTY--IPR FFDARRKWRH CSTIGRVRDQ GNCGSCWAVA
MpCathB9    EGVQIPNKVN FKMYKTKDDN Y-NSYVEIPI KFDSRKKWTR CKTIGEVRDQ GNCRSDWALS

            121                          150                                 180
MpCathB3    TSSAFADRLC VATNADFNEL LSAEEITFCC HSCGFGCDGG YPIKAWERFS KHGLVTGGDY
MpCathB6    TSSAFADRLC VATNADFNEL LSAEEITFCC HTCGFGCNGG YPIKAWKRFS KKGLVTGGDY
MpCathB9    TSSAFSDRLC VATNGDFNQL LSAEEITFCC HNCGDGCSGG HPIRAWKRFN KHGLVTGGDY
                                     Mature domain
            181                          210                                 240
MpCathB3    KSGEGCEPYR VPPCPYDEQG NNTCAGKPME SNHRCTRMCY GNQDLDFDQD HRYTRDYYYL
MpCathB6    KSGEGCEPYR VPPCPNDDQG NNTCAGKPME SNHRCTRMCY GDQDLDFDED HRYTRDYYYL
MpCathB9    KSDQGCQPYT VPPCPYDEQG KKTCSDQPME SNHKCMTTCY GEKDLDYQKD HRYTRDYYNL

            241                           270                                300
MpCathB3    TYGSIQKDVM TYGPIEASFD VYDDFPSYKS GVYVKSENAT YLGGHAAKLI GWGEEYGVPY
MpCathB6    TYGSIQKDVM TYGPIEASFD VYDDFPSYKS GVYVKSENAS YLGGHAVKLI GWGEEYGVPY
MpCathB9    NYRIIQKDLM NYGPIEASFD VYDDFPSYKS GIYVKSENAS YLGGHSVKLI GWGEEYGVLY

            301                           330        340
MpCathB3    WLMVNSWNAD WGDNGLFKIQ RGTNECGIDN STTAGVPVTN
MpCathB6    WLMVNSWNED WGDNGFFKIQ RGTNECGVDN STTAGVPVTN
MpCathB9    WLMVNSWNSD WGDKGLFKIR RGTNECGVDN STTGGVPAT-
```

Fig. S3. Multiple sequence alignment of *M. persicae* CathB3, 6 and 9 proteins using MUSCLE. The conserved residues are marked in grey background. Signal peptide and prodomain are labelled with rectangles. Mature domain is marked with a yellow line underneath the alignment. The catalytic cysteine is indicated as red font in yellow background and marked with a red asterisk. The catalytic histidine and asparagine are highlighted in cyan background.

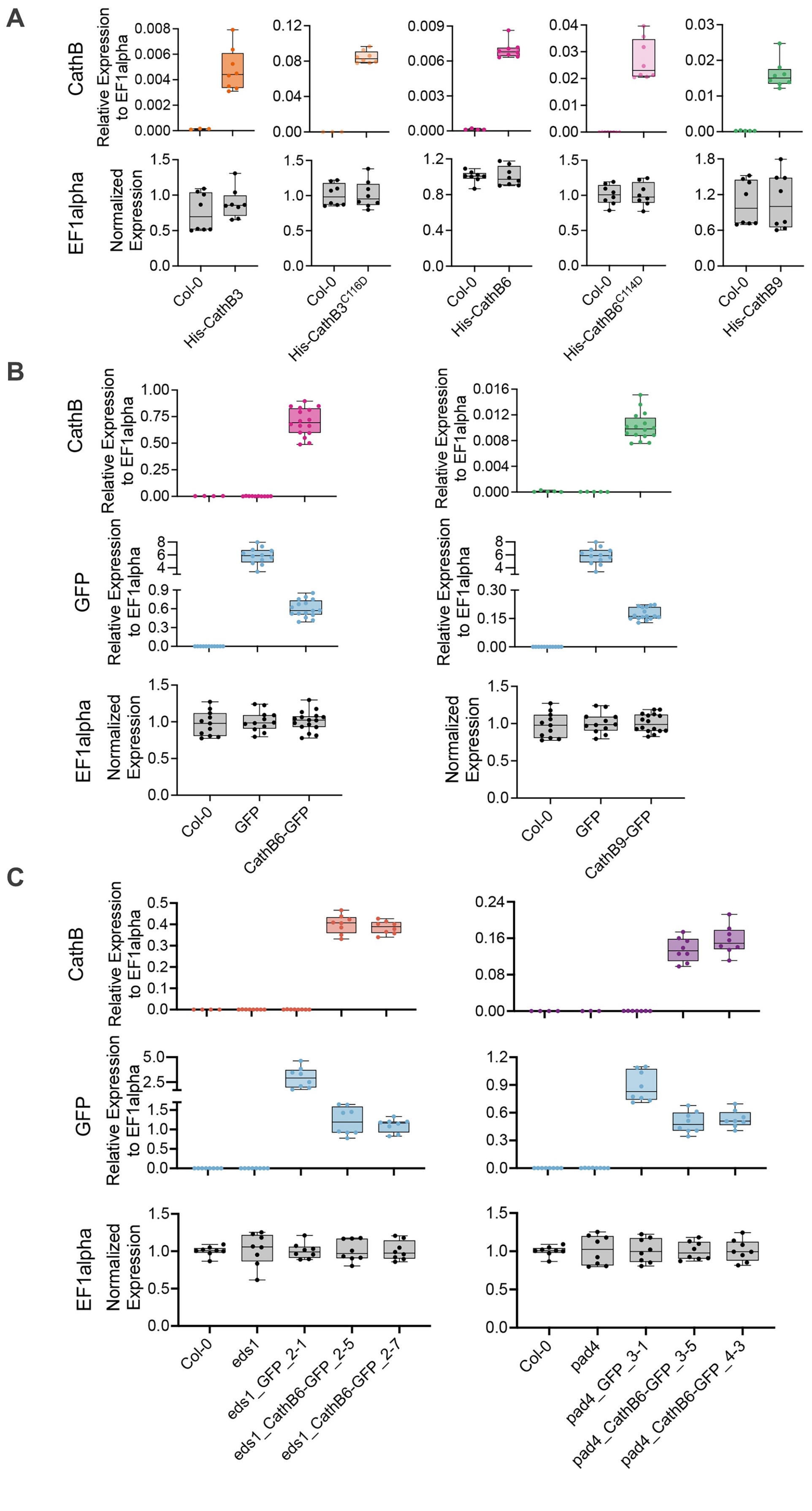
A
CathB
Relative Expression to EF1alpha
EF1alpha
Normalized Expression
Col-0
His-CathB3
His-CathB3C116D
His-CathB6
His-CathB6C114D
His-CathB9
B
GFP
CathB6-GFP
CathB9-GFP
C
eds1
eds1_GFP_2-1
eds1_CathB6-GFP_2-5
eds1_CathB6-GFP_2-7
pad4
pad4_GFP_3-1
pad4_CathB6-GFP_3-5
pad4_CathB6-GFP_4-3

Fig. S4. Quantification of transgene expression levels in CathB3, B6 and B9 stable transgenic *A. thaliana* lines used for *M. persicae* fecundity assay. (**A** to **C**) Quantifications of transgene transcript levels by qRT-PCR using specific primers to CathB3 (and $B3^{C116D}$), B6 (and $B6^{C114D}$) and B9 (A) and CathB6 and GFP (B and C). Samples for transgene quantification were collected from three single plants and quantified with n = 3 to 12 repeats (dots on boxplots). Relative transcript levels were determined by qRT-PCR and normalized to the internal control EF1alpha. This figure relates to Figures 1C, 1D and 4C.

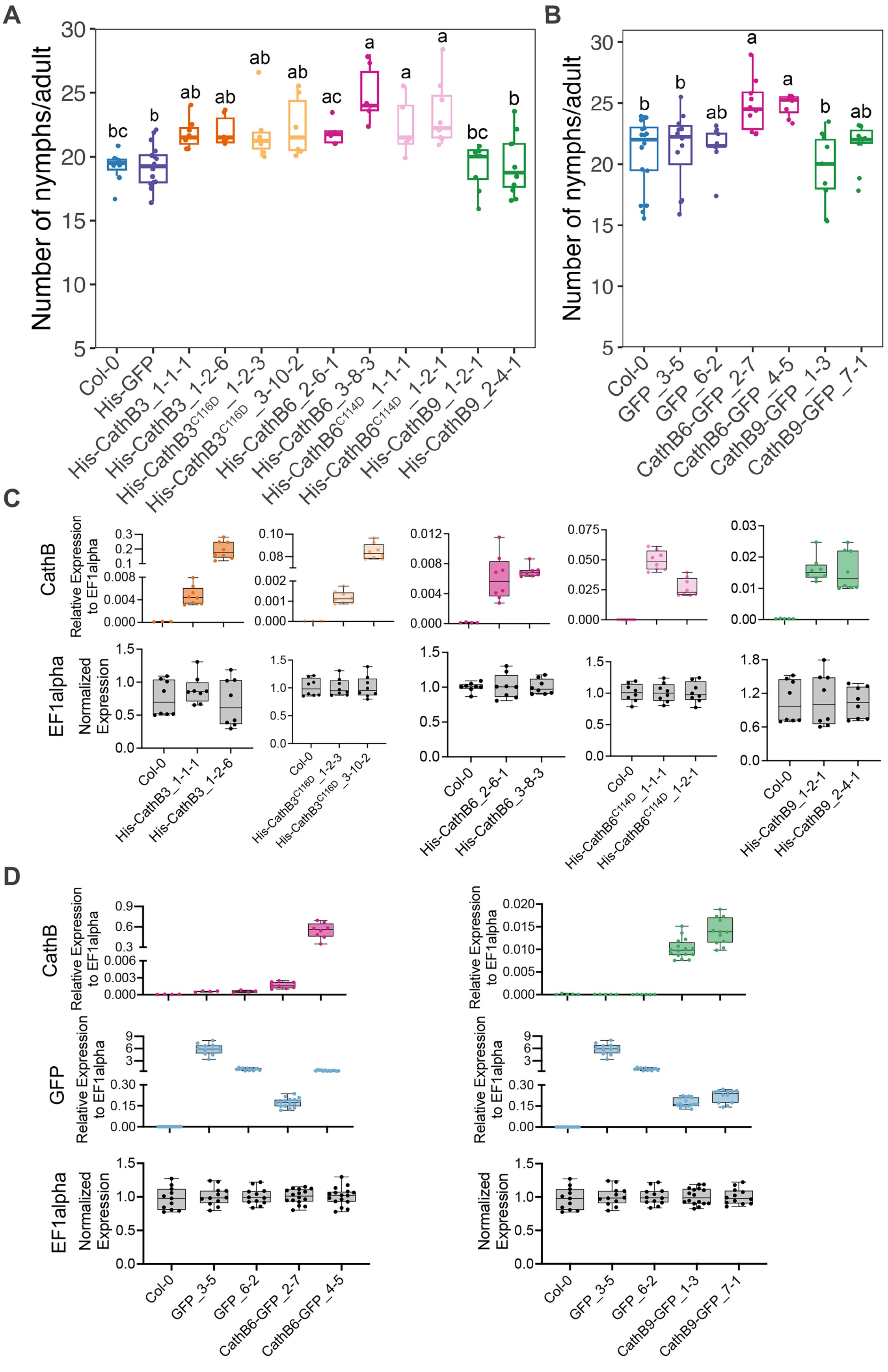
A
B
C
D
Number of nymphs/adult
30
25
20
15
10
5
bc
b
ab
ab
ab
ab
ac
a
a
a
bc
b
Col-0
His-GFP
His-CathB3_1-1-1
His-CathB3_1-2-6
His-CathB3^C116D_1-2-3
His-CathB3^C116D_3-10-2
His-CathB6_2-6-1
His-CathB6_3-8-3
His-CathB6^C114D_1-1-1
His-CathB6^C114D_1-2-1
His-CathB9_1-2-1
His-CathB9_2-4-1
b
b
ab
a
a
b
ab
GFP_3-5
GFP_6-2
CathB6-GFP_2-7
CathB6-GFP_4-5
CathB9-GFP_1-3
CathB9-GFP_7-1
CathB
Relative Expression to EF1alpha
EF1alpha
Normalized Expression
GFP

Fig. S5. ***M. persicae*** **reproduction is enhanced on transgenic plants stably expressing CathB3, CathB6, or their catalytic mutants, but not on plants expressing CathB9.** (**A** and **B**) Aphid fecundity assays on stable His-GFP, His-CathB3, His-CathB3^{C116D}, His-CathB6, His-CathB6^{C114D}, and His-CathB9 (A) and GFP, CathB6-GFP and CathB9-GFP (B) transgenic *A. thaliana* lines. Box plots show the distribution of nymphs produced by an adult female aphid per plant (dots) collected from n = 6 to 16 female aphids per *A. thaliana* line. Statistical significance was determined by ANOVA (Tukey method, $p < 0.05$) and presented as different letters. (**C** and **D**) Quantifications of transgene transcript levels by qRT-PCR using specific primers to CathB3 (and B3^{C116D}), B6 (and B6^{C114D}) and B9 (C) and CathB6 and GFP (D). Samples for transgene quantification were collected from three single plants and quantified with n = 3 to 12 repeats (dots on boxplots). Quantification refers to relative transcript levels which were determined by qRT-PCR and normalized to the internal control EF1alpha.

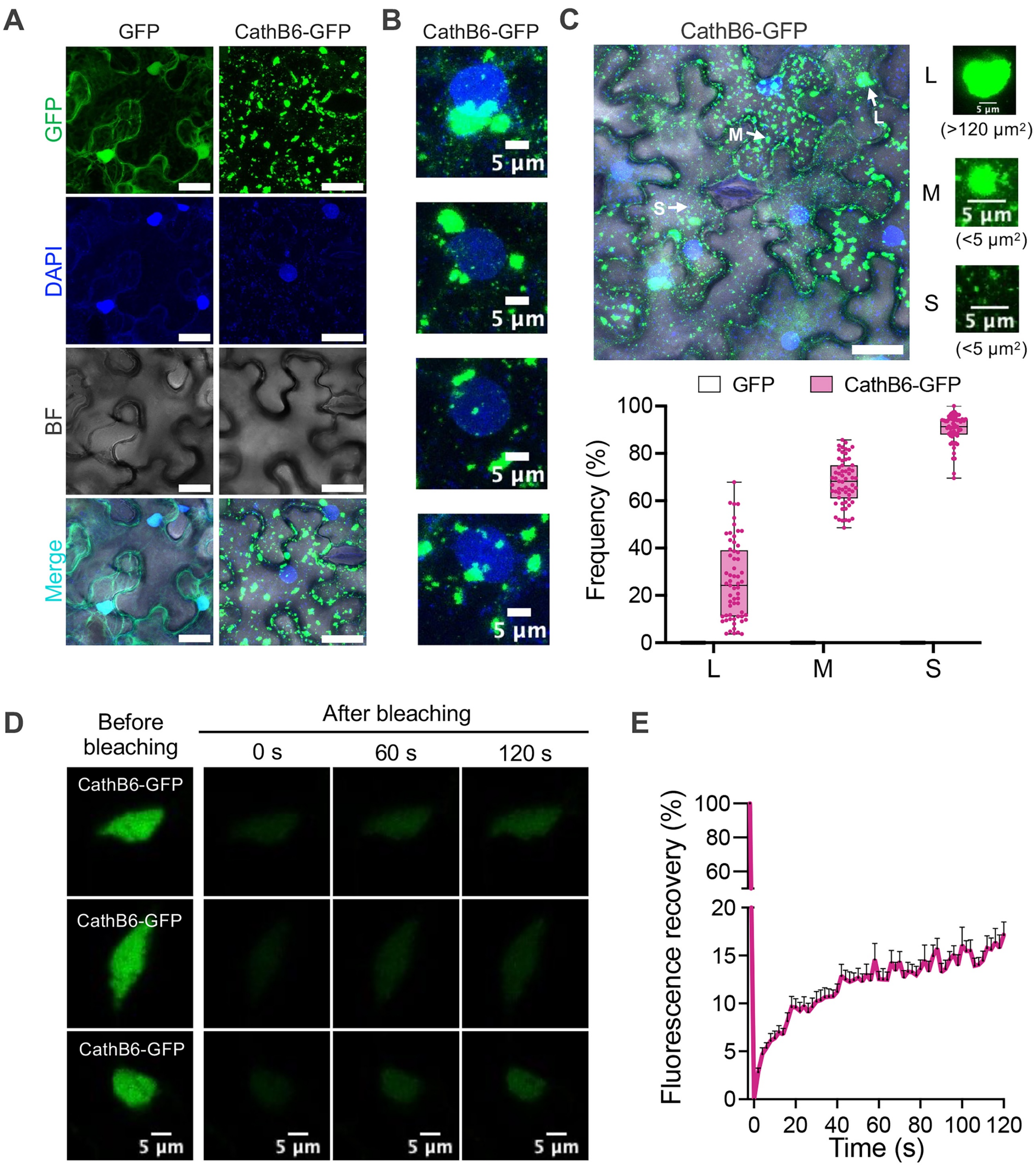

Fig. S6. *M. persicae* CathB6 locates to mobile puncta within plant cells. (**A**) Confocal images of GFP or CathB6-GFP in *N. benthamiana* cells. CathB6-GFP forms distinct puncta that are not seen for GFP. (**B**) CathB6-GFP puncta position adjacently but not inside plant cell nuclei. (**C**) Classification of CathB6-GFP puncta into three size categories: Large (L), Medium (M), and Small (S) based on area. Puncta frequency was determined as the ratio of cells with CathB6-GFP puncta to the total number of cells observed in the field. Data were compiled from 61 observation fields across three independent experiments, with 22–40 cells per field. Scale bars in (A) and (C), 30 µm. (A)-(C), Nuclei are stained with 4′,6-diamidino-2-phenylindole (DAPI). BF, Bright field. (**D**)

Fluorescence recovery after photobleaching (FRAP) analysis of CathB6-GFP puncta. Representative FRAP images show fluorescence recovery over time, with Time 0 marking the photobleaching pulse. Data are representative of 25 independent experiments. (**E**) Time-course plot of fluorescence recovery for CathB6-GFP puncta after photobleaching. Recovery was quantified as the percentage of fluorescence intensity post-bleaching relative to pre-bleaching intensity. Data are presented as mean ± s.e.m. (n = 25).

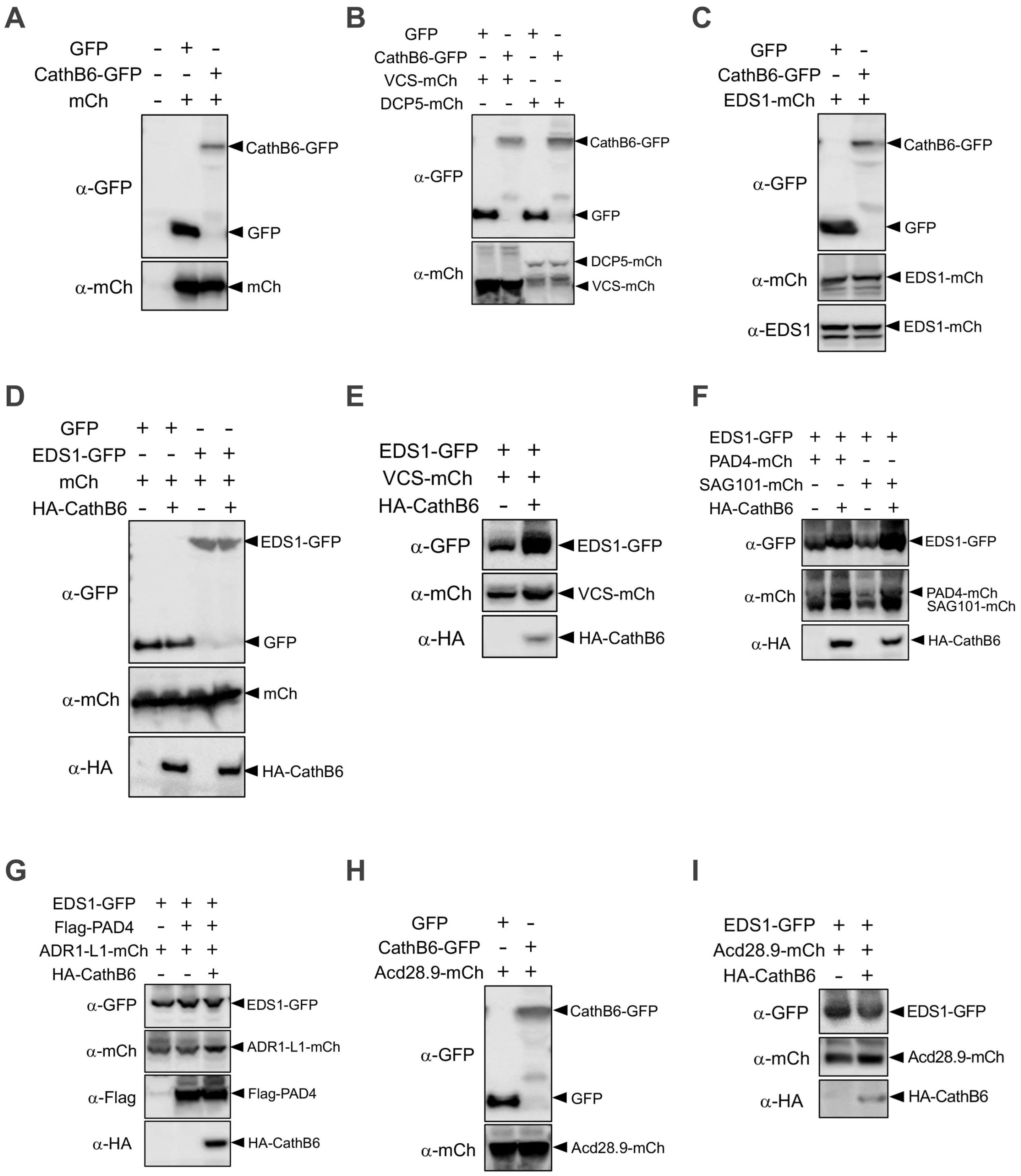

Fig. S7. Western blots showing the presence of expected proteins in agroinfiltrated *N. benthamiana* leaf sections used for confocal microscopy experiments. (**A**) GFP, CathB6-GFP and mCherry, relates to Fig. 2A and fig. S6. (**B**) GFP, CathB6-GFP, VCS-mCherry and DCP5-mCherry, relates to Fig. 2, C and D , and fig. S10, A and B. (**C**) GFP, CathB6-GFP and EDS1-mCherry, relates to Fig. 3C. (**D**) GFP, EDS1-GFP, mCherry and HA-CathB6, relates to fig. S17A. (**E**) EDS1-GFP, VCS-mCherry and HA-CathB6, relates to Fig. 3D. (**F**) EDS1-GFP, PAD4-mCherry, SAG101-mCherry and HA-CathB6, relates to Fig. 3E and fig. S17B. (**G**) EDS1-GFP,

Flag-PAD4, ADR1-L1-mCherry and HA-CathB6, relates to Fig. 3F. (**H**) GFP, CathB6-GFP and Acd28.9-mCherry, relates to Fig. 5B. (**I**) EDS1-GFP, Acd28.9-mCherry and HA-CathB6, relates to Fig. 5C. The protein sizes are: GFP, 26.9 kDa; CathB6-GFP, 59.9 kDa; EDS1-GFP, 98.9 kDa; mCherry, 26.7 kDa; VCS-mCherry, 64.7 kDa; DCP1-RFP, 67.3 kDa; DCP5-mCherry, 91.3 kDa; EDS1-mCherry, 98.6 kDa; PAD4-mCherry, 87.9 kDa; SAG101-mCherry, 89.0 kDa; ADR1-L1-mCherry, 118.8 kDa; Acd28.9-mCherry, 55.9 kDa; HA-CathB6, 36.6 kDa; Flag-PAD4, 65.6 kDa.

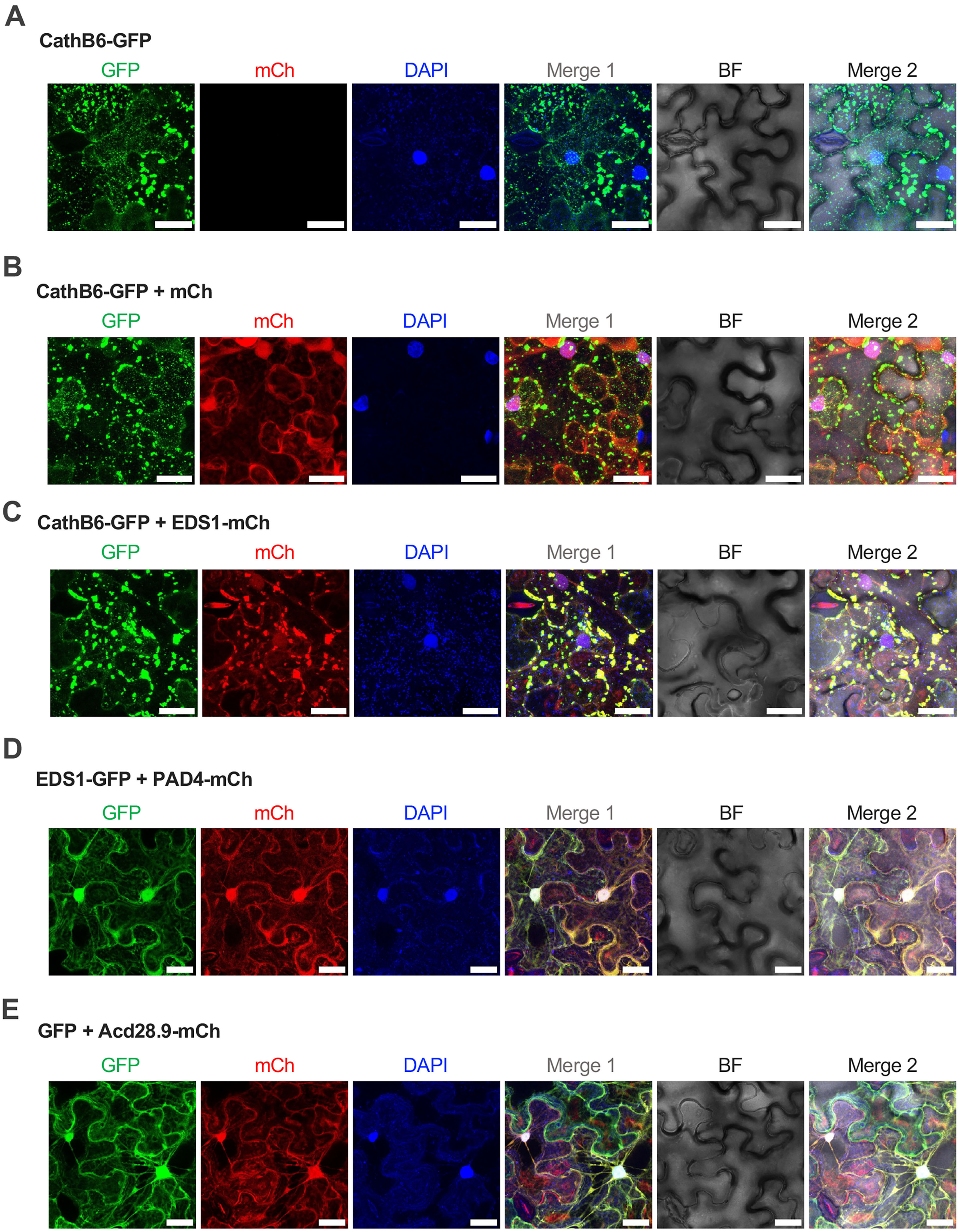

Fig. S8. Localization of CathB6-induced puncta relative to plant cell nuclei. DAPI was used to visualize the nuclei in the presence of CathB6-GFP alone (**A**), CathB6-GFP and mCherry (**B**), CathB6-GFP and EDS1-mCherry (**C**), EDS1-GFP and PAD4-mCherry (**D**), and GFP and

Acd28.9-mCherry (**E**). Merge 1 overlay the GFP, mCherry (mCh) and DAP1 images, and Merge 2 overlay the Bright field (BF) and Merge 1 images. Scale bars, 30 μm.

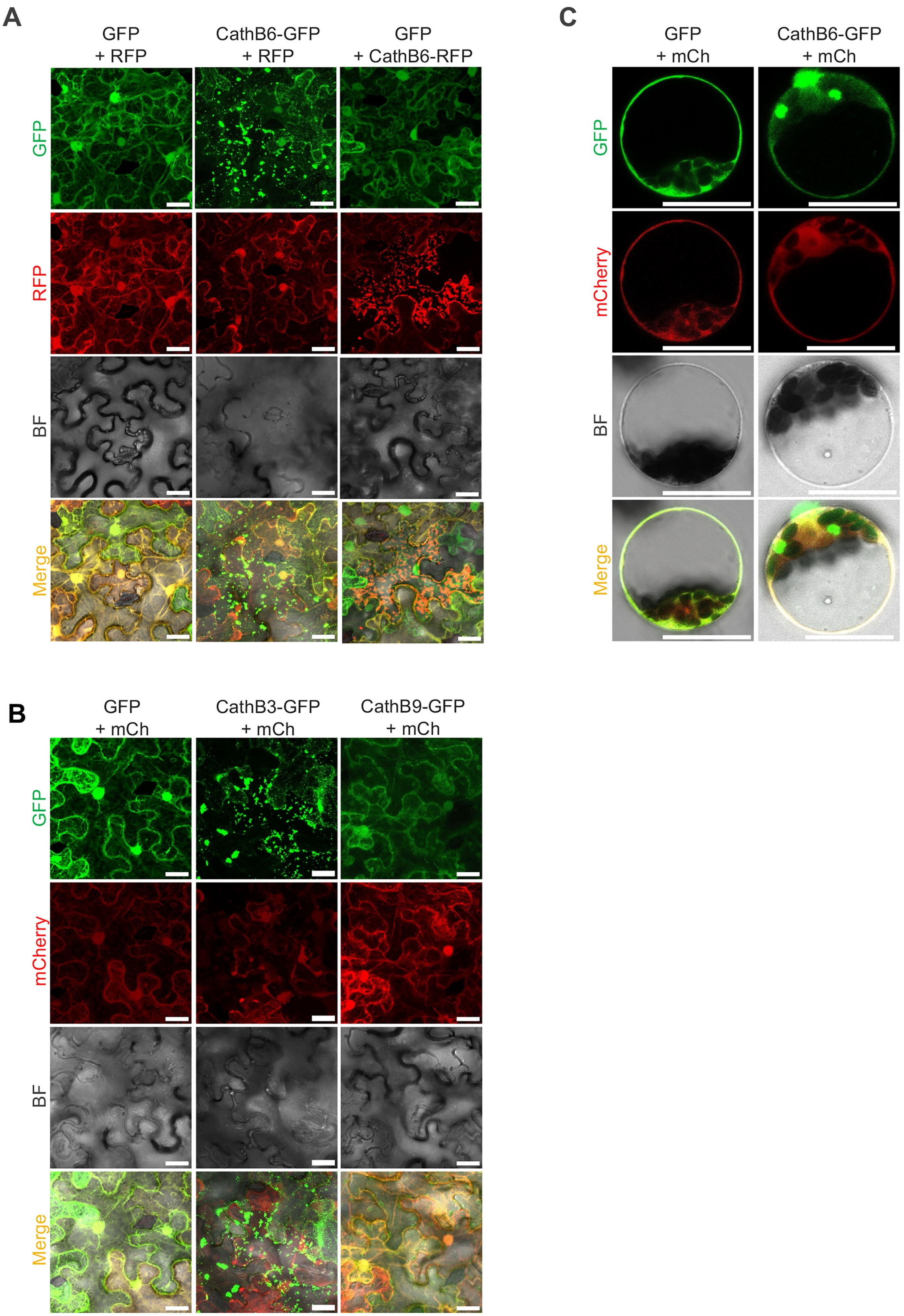
A
GFP
+ RFP
CathB6-GFP
+ RFP
GFP
+ CathB6-RFP
GFP
RFP
BF
Merge
B
GFP
+ mCh
CathB3-GFP
+ mCh
CathB9-GFP
+ mCh
GFP
mCherry
BF
Merge
C
GFP
+ mCh
CathB6-GFP
+ mCh
GFP
mCherry
BF
Merge

Fig. S9. *M. persicae* CathB6 and CathB3, and to a lesser extent CathB9, form cytoplasmic puncta in *N. benthamiana* epidermal cells and *A. thaliana* protoplasts. (**A**) CathB6 forms puncta in *N. benthamiana* cells when fused to GFP (CathB6-GFP) or RFP (CathB6-RFP), with free RFP or GFP as internal references. (**B**) CathB3-GFP forms puncta of varying sizes in *N. benthamiana* cells, whereas CathB9-GFP generates significantly fewer puncta. (**C**) CathB6-GFP forms puncta of varying sizes in *A. thaliana* protoplasts. Scale bars, 30 µm
.

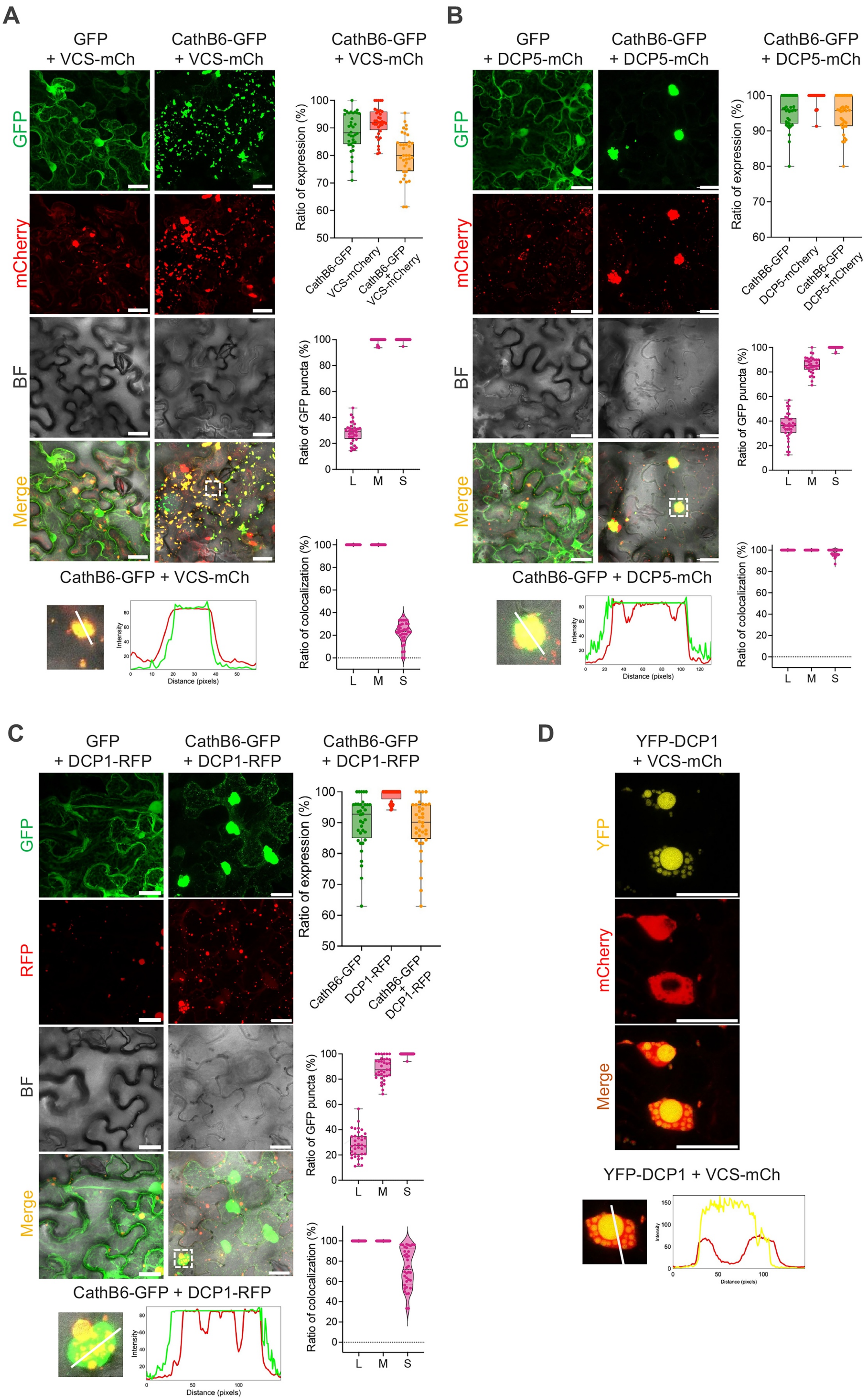
A
GFP + VCS-mCh
CathB6-GFP + VCS-mCh
CathB6-GFP + VCS-mCh
GFP
mCherry
BF
Merge
Ratio of expression (%)
CathB6-GFP
VCS-mCherry
CathB6-GFP + VCS-mCherry
Ratio of GFP puncta (%)
L
M
S
Ratio of colocalization (%)
CathB6-GFP + VCS-mCh
Intensity
Distance (pixels)
B
GFP + DCP5-mCh
CathB6-GFP + DCP5-mCh
CathB6-GFP + DCP5-mCh
DCP5-mCherry
CathB6-GFP + DCP5-mCherry
CathB6-GFP + DCP5-mCh
C
GFP + DCP1-RFP
CathB6-GFP + DCP1-RFP
CathB6-GFP + DCP1-RFP
RFP
DCP1-RFP
CathB6-GFP + DCP1-RFP
CathB6-GFP + DCP1-RFP
D
YFP-DCP1 + VCS-mCh
YFP
mCherry
Merge
YFP-DCP1 + VCS-mCh

Fig. S10. CathB6-GFP colocalizes with p-body markers VCS, DCP5 and DCP1 in plant cells. (**A** to **C**) CathB6-GFP colocalizes with VCS-mCherry (A), DCP5-mCherry (B) and DCP1-RFP (C) in *N. benthamiana* cells, unlike GFP alone (control). Panels at right of the confocal images provide quantification of colocalization from three independent experiments across 36 observation fields. Top: ratios of cells expressing CathB6-GFP and/or VCS-mCherry/DCP5-mCherry/DCP1-RFP. Middle: proportions of cells with large, medium, and small CathB6-GFP puncta colocalized with VCS-mCherry/DCP5-mCherry/DCP1-RFP. Bottom: Proportions of VCS-mCherry/DCP5-mCherry/DCP1-RFP puncta colocalized with CathB6-GFP in cells with CathB6-GFP. (**D**) Confocal images of YFP-DCP1 and VCS-mCherry in *N. benthamiana* cells, showing YFP-DCP1 localizing within VCS-mCherry puncta. Graphs below the confocal images in (A) to (D) are intensity profiles along the marked lines of one of the puncta in the confocal image above. Scale bars, 30 μm.

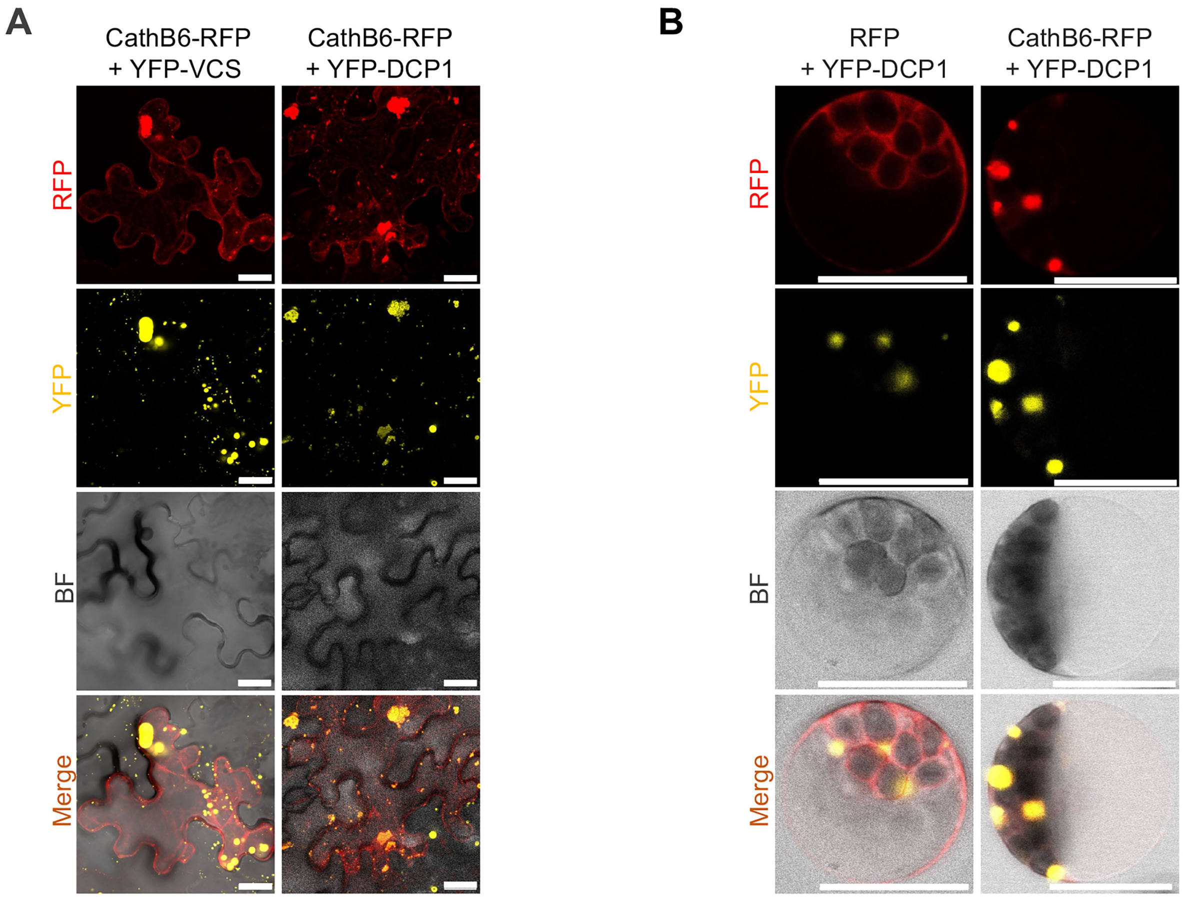

Fig. S11. CathB6-RFP colocalizes with p-body markers YFP-VCS and YFP-DCP1. *N. benthamiana* leaf cells (**A**) and *A. thaliana* protoplasts (**B**). Scale bars, 30 µm (A) and 20 µm (B).

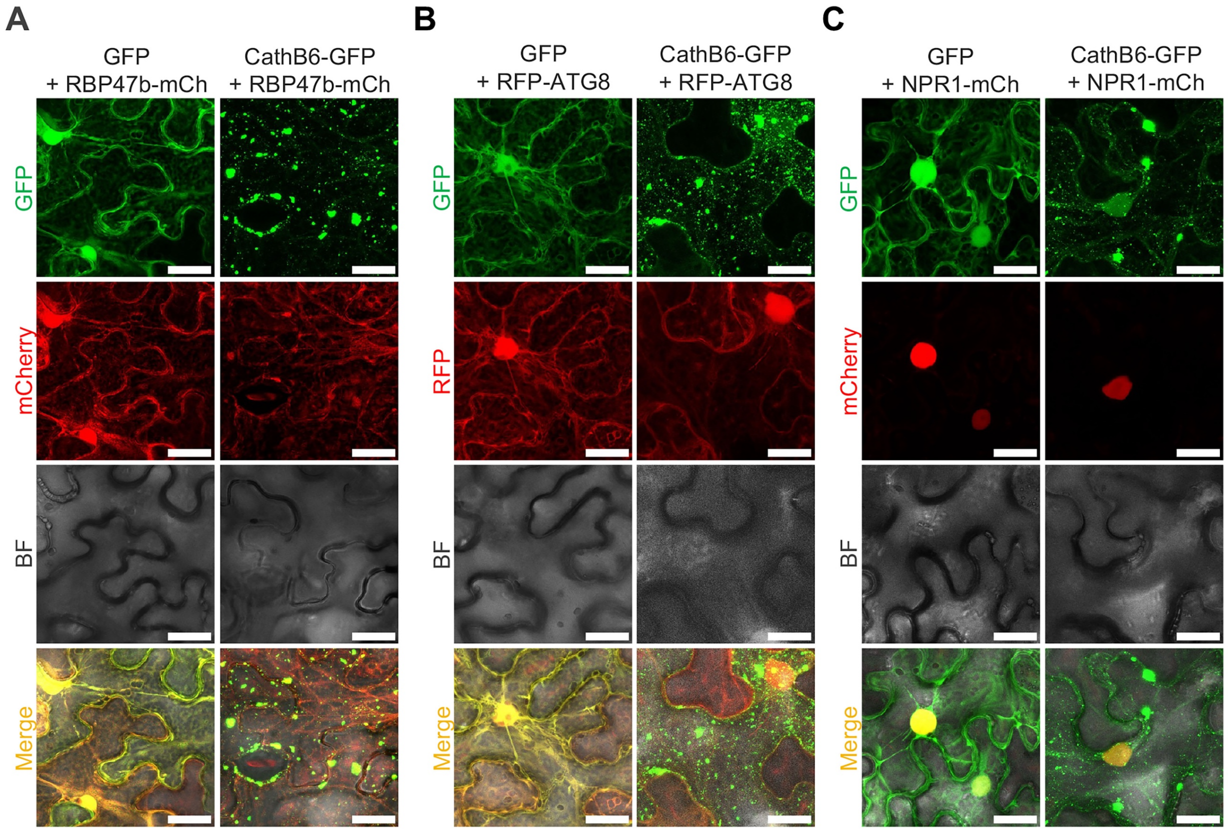

Fig. S12. CathB6-GFP puncta partially colocalize with the stress granule marker RBP47b-mCherry (A), while no obvious colocalizations were observed with RFP-ATG8 (B) or NPR1-mCherry (C) in *N. benthamiana* epidermal cells. Scale bars, 30 µm.

Protein	Accession	Unique peptides	Normalized FC	*p*-value
• GFP		19	0.01	1E-17
M. persicae				
• CathB6	MYZPE13164_O_EIv2.1_0111090	20	100	1E-17
***A. thaliana* (267)**				
Proteins involved in plant immunity (76)				
• Enhanced disease susceptibility, EDS1	AT3G48090	12	8.015	0.0072
• Heat shock protein HSP20/alpha crystallin family, Acd28.9	AT5G47590	21	33.686	0.0008
…	…	…	…	…

Fig. S13. CathB6-TurboID PL-MS identifies EDS1 and Acd28.9 as CathB6 interactors in *A. thaliana*. *A. thaliana* proteins identified from CathB6-TurboID PL-MS were screened with a foldchange of CathB6-TurboID/GFP-TurboID > 2 and *p*-value < 0.05. Nineteen (19) unique peptides matching GFP were identified in the GFP-TurboID sample, and 20 matching CathB6 in the CathB6-GFP TurboID sample. Among a total of 267 *A. thaliana* proteins identified in the CathB6-GFP TurboID sample, 76 proteins are in involved in regulating plant immunity, and these include EDS1 and Acd28.9. Full CathB6-TurboID PL-MS dataset is shown in (*27*).

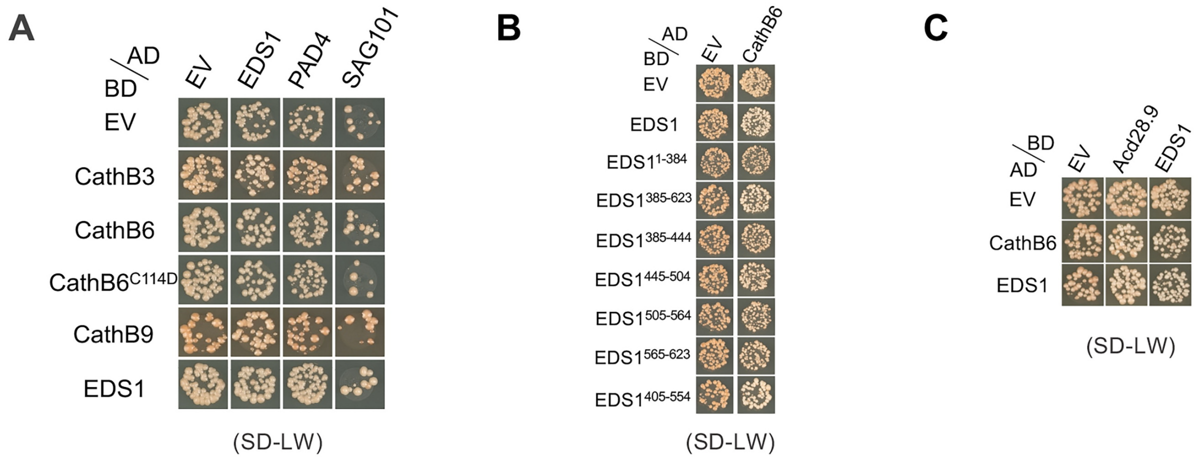

Fig. S14. Yeast growth on double dropout medium lacking leucine and tryptophan indicating the expression of AD and BD constructs in Y2H assays. Related to Figure 3A (A), Figure 3B (B) and Figure 5A (C) of the main text.

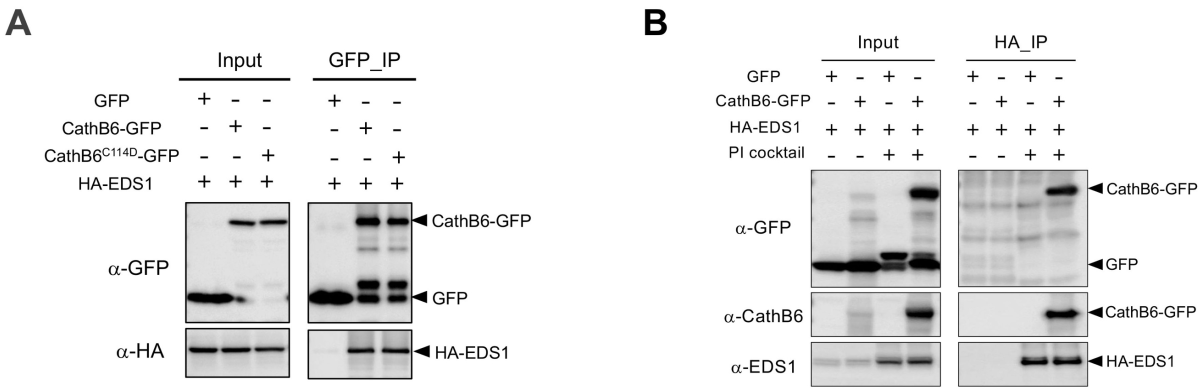

Fig. S15. ***M. persicae*** **CathB6 interacts with EDS1** ***in planta*** **and** ***in vitro*****.** (**A**) Co-IP of HA-EDS1 with CathB6-GFP and CathB6^{C114D}-GFP from agroinfiltrated *N. benthamiana* leaves using GFPtrap beads. (**B**) Co-IP of CathB6-GFP with HA-EDS1 from insect cell (Sf9) extracts, using anti-HA beads. The addition of protease inhibitor (PI) cocktail improved CathB6 and EDS1 stability in co-IP experiments. Arrowheads indicate bands of the expected sizes. The protein sizes are: GFP, 26.9 kDa; CathB6-GFP, 59.9 kDa; HA-EDS1, 76.7 kDa.

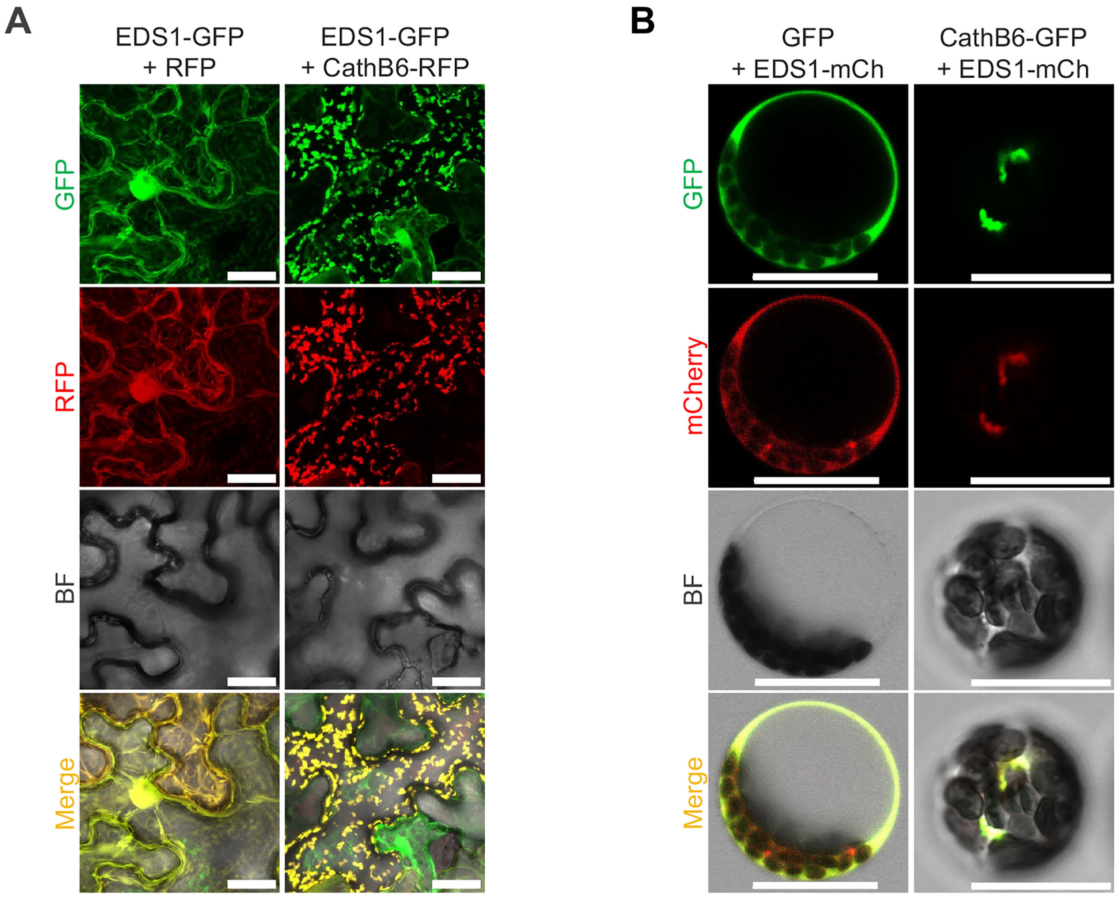

Fig. S16. CathB6-RFP relocates EDS1-GFP to puncta in *N. benthamiana* epidermal cells (A) and *A. thaliana* protoplasts (B). Scale bars, 30 µm.

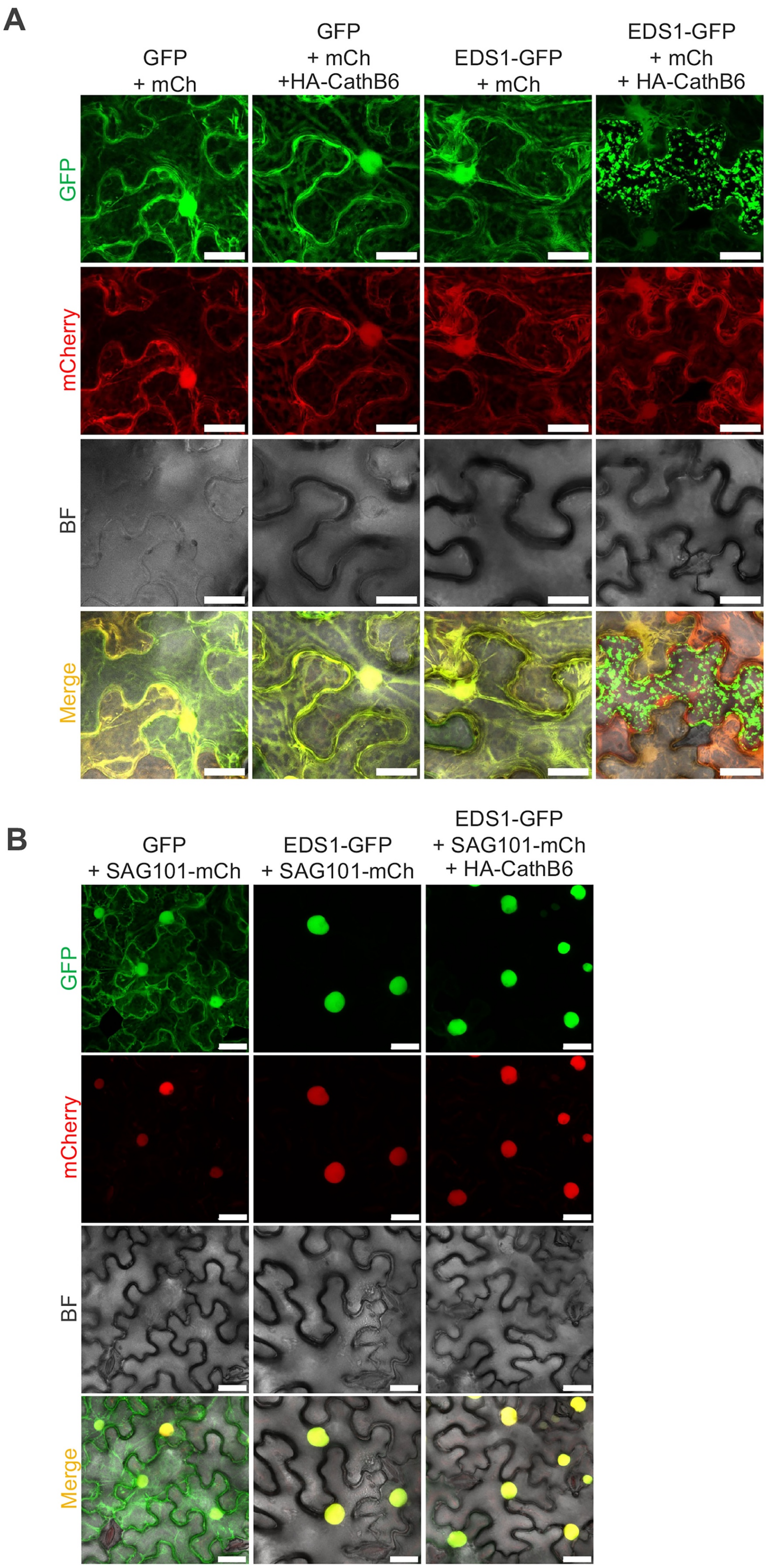
A
GFP
+ mCh
GFP
+ mCh
+HA-CathB6
EDS1-GFP
+ mCh
EDS1-GFP
+ mCh
+ HA-CathB6
GFP
mCherry
BF
Merge
B
GFP
+ SAG101-mCh
EDS1-GFP
+ SAG101-mCh
EDS1-GFP
+ SAG101-mCh
+ HA-CathB6
GFP
mCherry
BF
Merge

Fig. S17. ***M. persicae*** **CathB6 relocalizes EDS1 alone localizes to cytoplasmic puncta/p-bodies (A), unlike the EDS1-SAG101 complex that remains localized to nuclei (B)**. Images show selected cells of *N. benthamiana* leaves infiltrated with various plasmids for the expression of various proteins. Scale bars, 30 µm.

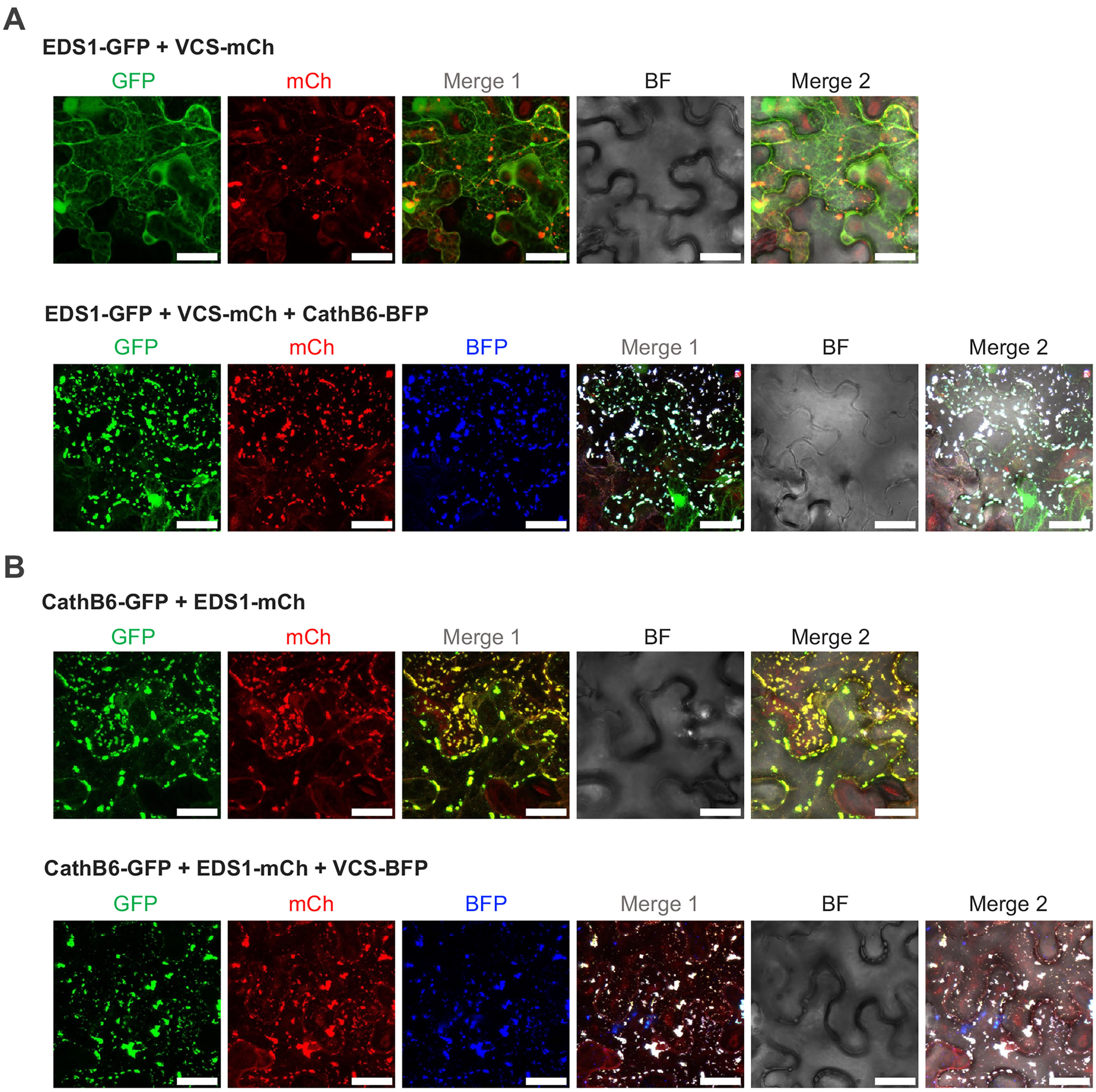

Fig. S18. Three-partite subcellular analysis show that CathB6 relocate EDS1 to p-bodies in *N. benthamiana* epidermal cells. (A) CathB6-BFP relocate EDS1-GFP to the position of VCS-mCherry. (B) CathB6-GFP relocate EDS1-GFP to the puncta labelled by VCS-BFP. Scale bars, 30 μm.

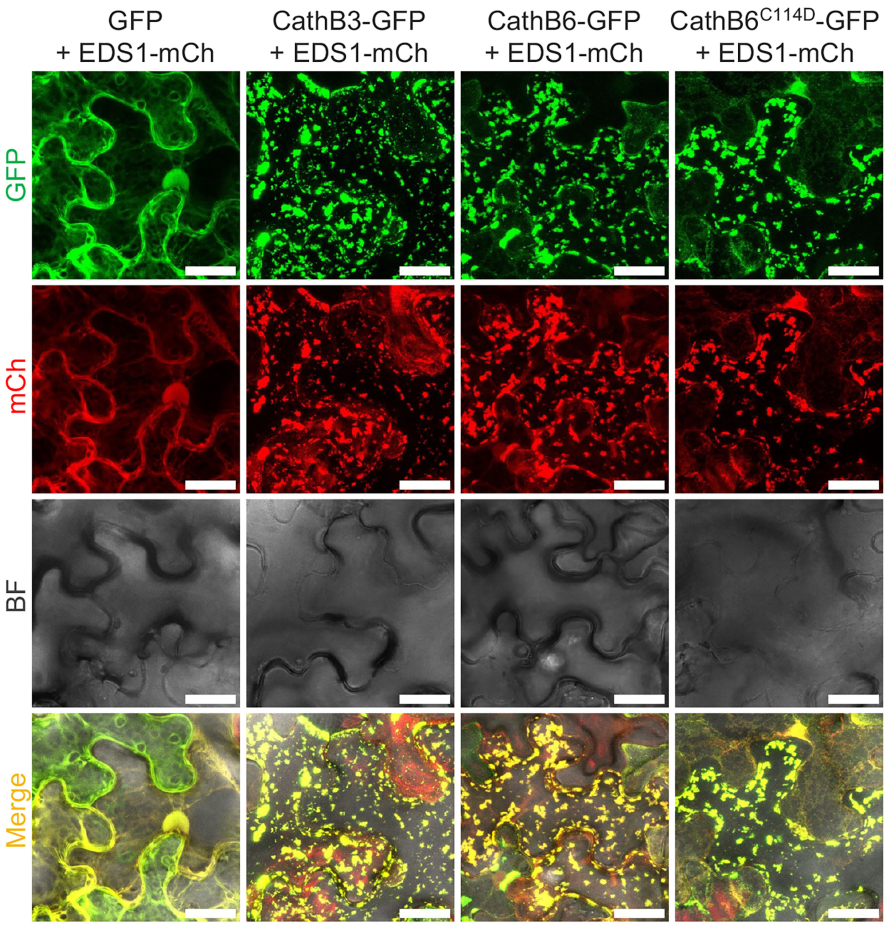

Fig. S19. CathB3, CathB6 and CathB6^{C114D}-GFP relocate EDS1-GFP to puncta in *N. benthamiana* epidermal cells. Scale bars, 30 µm.

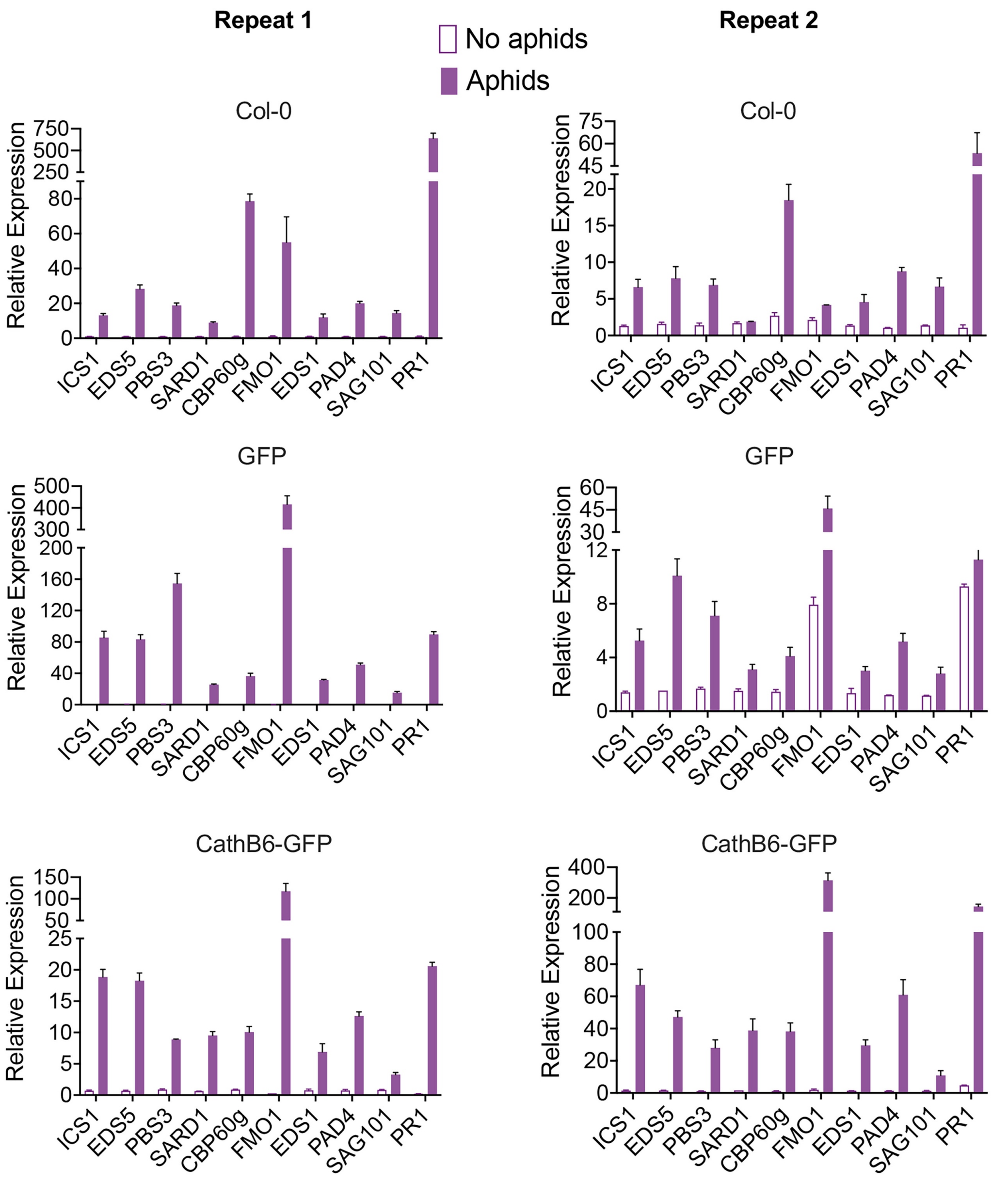

Fig. S20. ***M. persicae*** **feeding induces variable upregulation of EDS1 and SAR-related genes on different** ***A. thaliana*** **lines.** Two biological repeats of aphid infestation on *A. thaliana* wild-type Col-0, GFP and CathB6-GFP transgenic lines show variable expression levels of *EDS1*, SA biosynthesis (*ICS1, EDS5, PBS3*) and SAR regulators (*PAD4, SAG101, SARD1, CBP60g, FMO1, PR1*).

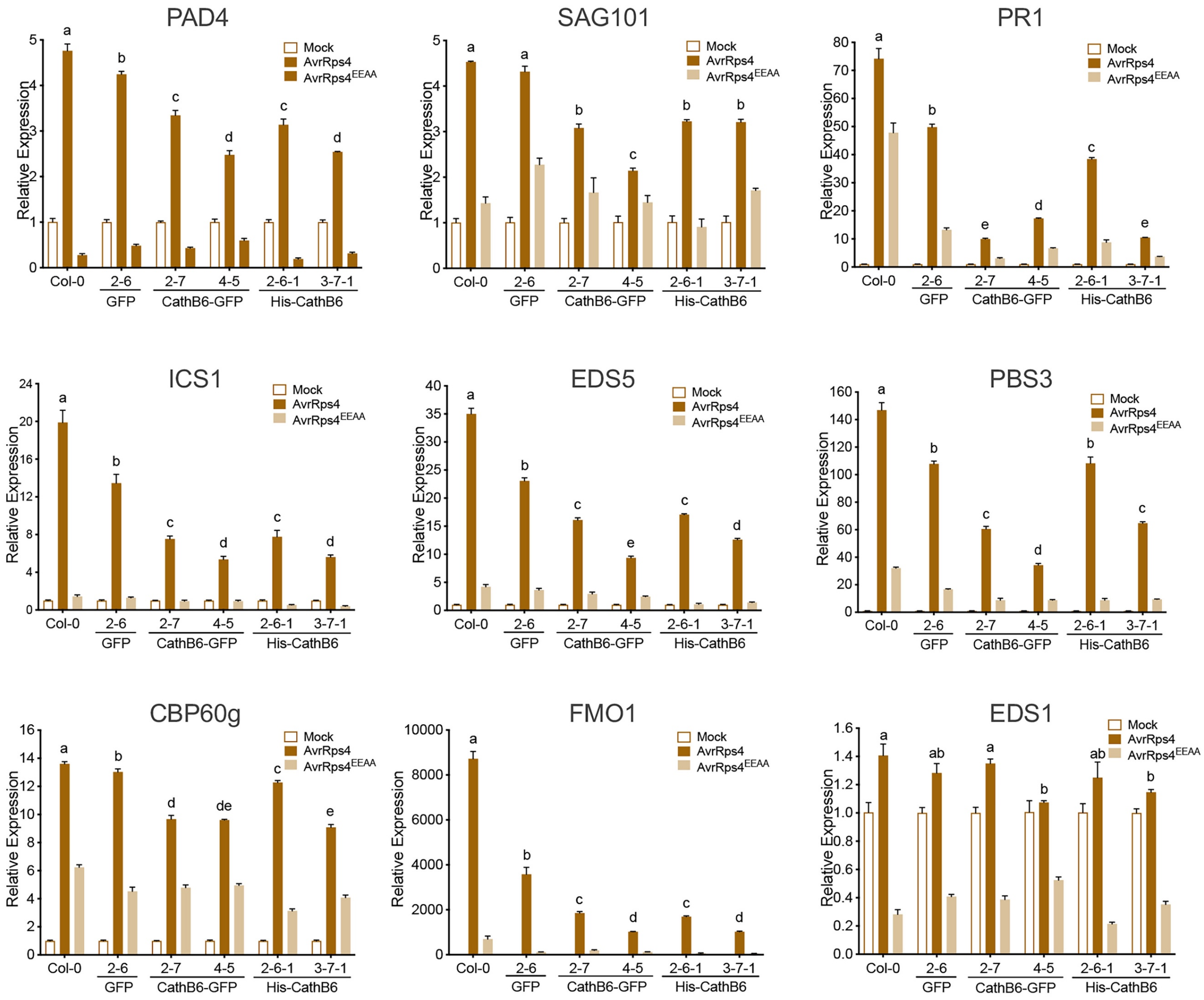

Fig. S21. ***M. persicae*** **CathB6 suppresses EDS1-responsive genes regulating systemic acquired resistance (SAR).** Quantifications of transcript levels of EDS1 and SAR-related genes *ICS1, EDS5, PBS3* (SA biosynthesis) and *PAD4, SAG101, CBP60g, FMO1, PR1* (SAR regulators) in response to mock, Pf0-1 AvrRps4, or Pf0-1 AvrRps4^{EEAA} treatments using gene-specific primers. Data represent means ± s.e.m. from data of two biological replications; significant differences were analyzed by ANOVA. More replicates are shown in Fig. 4D.

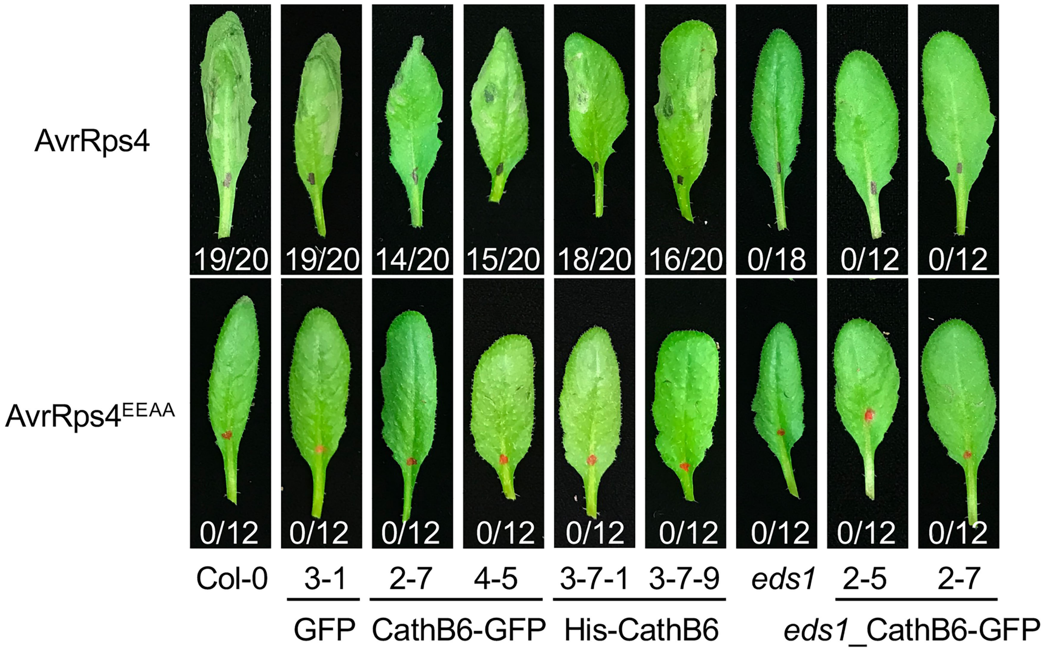

Fig. S22. *M. persicae* CathB6 does not have obvious hypersensitive response (HR) suppression activity. Photos are representative leaves selected from 12 to 20 leaves of stable His-CathB6 or CathB6-GFP *A. thaliana* lines in Col-0 and *eds1-2* backgrounds, after infiltrating Pf0-1 AvrRps4 and AvrRps4^{EEAA} for 24 h. HR response typically seen upon infiltration with AvrRps4, but not AvrRps4^{EEAA}, is evident from cell death symptoms on the leaves. The ratio beneath each leaf indicates number of leaves with visible tissue collapse in all infiltrated leaves from three independent experiments.

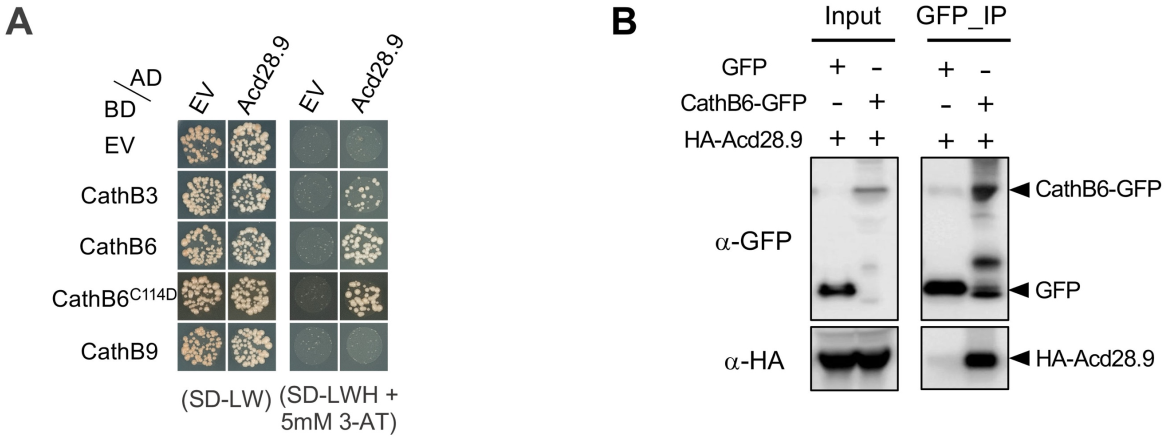

Fig. S23. ***M. persicae*** **CathB6 interacts with** ***A. thaliana*** **Hsp20 protein Acd28.9.** (**A**) Yeast two-hybrid assays showing Acd28.9 interaction with CathB3, CathB6 and CathB6^{C114D}. (**B**) Co-IP of HA-Acd28.9 with CathB6-GFP from agroinfiltrated *N. benthamiana* leaves using GFPtrap beads. Arrowheads indicate bands of the expected sizes. The protein sizes are: GFP, 26.9 kDa; CathB6-GFP, 59.9 kDa; HA-Acd28.9, 34.2 kDa.

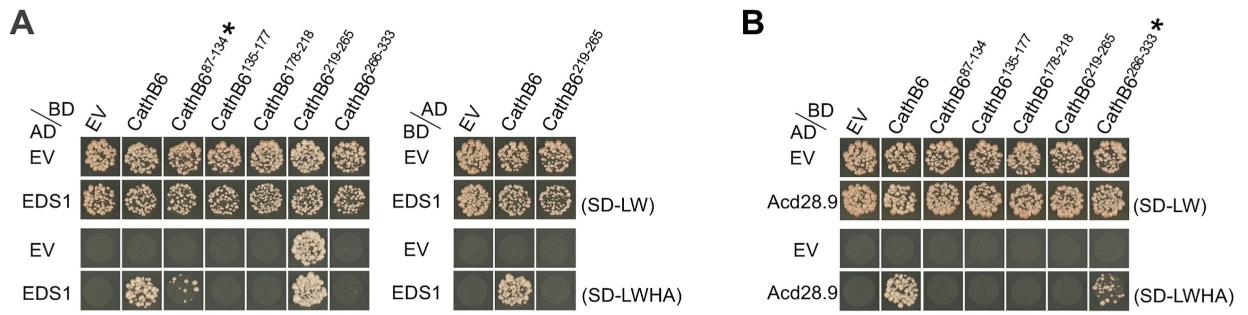

Fig. S24. Yeast two hybrid assays showing that different fragments of *M. persicae* CathB6 interact with EDS1 (A) versus Acd28.9 (B). EV, empty vector control. AD, GAL4-activation domain. BD, GAL4-DNA binding domain. SD-LW, double dropout medium lacking leucine and tryptophan. SD-LWHA, quadruple dropout medium lacking leucine, tryptophan, histidine, and adenine. * indicates the fragment interacting with EDS1 (**A**) or Acd28.9 (**B**).

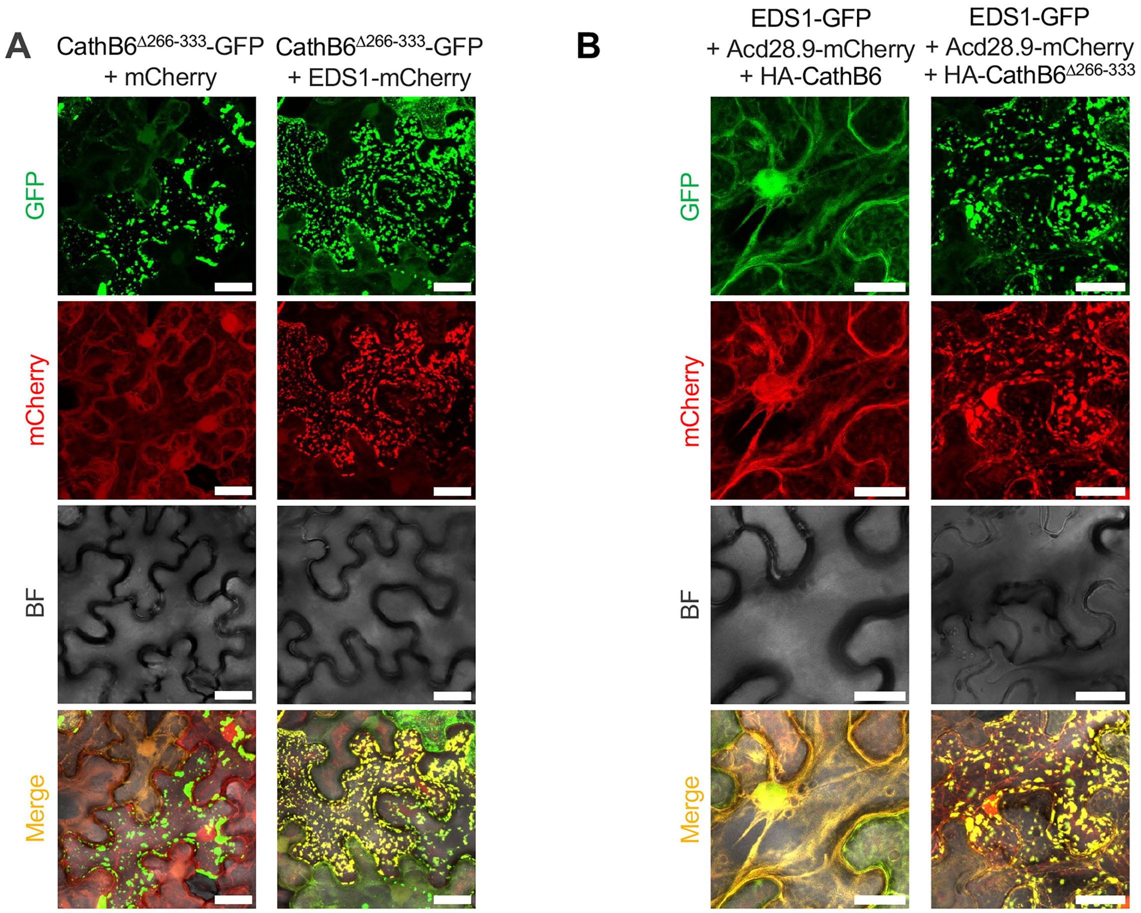

Fig. S25. The C-terminus of CathB6 is required for Acd28.9-mediated relocalization of EDS1 from p-bodies into the cytoplasm and cytoplasmic location of Acd28.9 in the presence of CathB6. (**A**)Localization of CathB6$^{\Delta 266\text{-}333}$-GFP and colocalization of CathB6$^{\Delta 266\text{-}333}$-GFP and EDS1-mCherry in puncta EDS1-GFP locates in the cytoplasm in the presence of full-length HA-CathB6 and Acd28.9-mCherry, whereas both EDS1-mCherry and EDS1-GFP locate in puncta in the presence of HA- CathB6$^{\Delta 266\text{-}333}$ (**B**). Confocal images feature plant cells from infiltrated *N. benthamiana* leaves. BF, bright field. Scale bars, 30 μm.

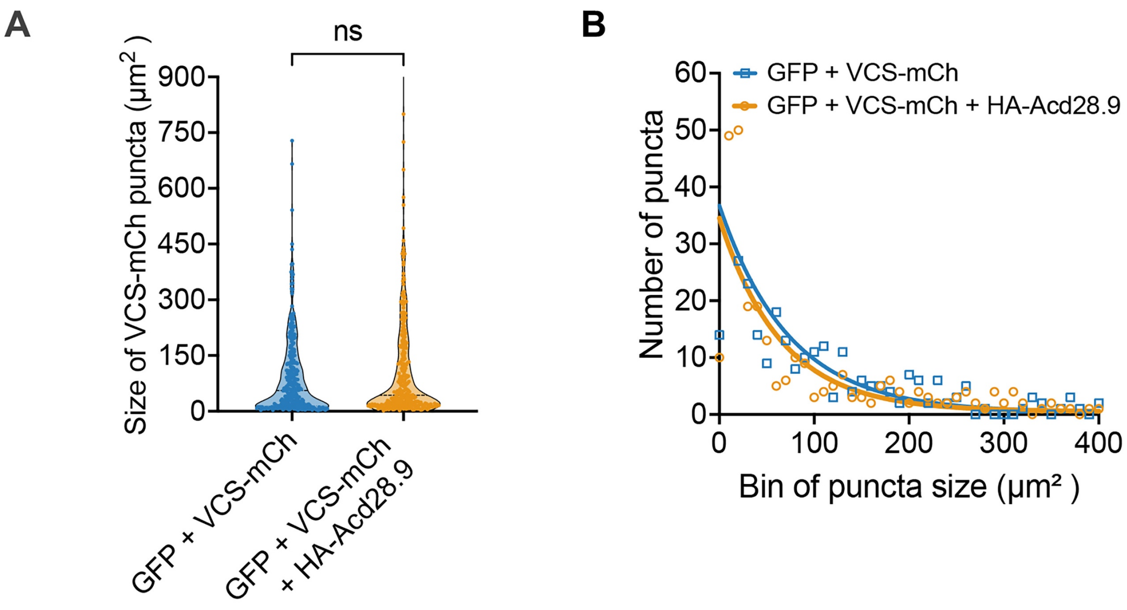

Fig. S26. Acd28.9 does not impact p-body formation in the absence of CathB6. (**A**) Analysis of VCS-mCherry puncta size when co-expressed with GFP or GFP and HA-Acd28.9. (**B**) Analysis of VCS-mCherry puncta distribution when co-expressed with GFP or GFP and HA-Acd28.9. Significance is assessed by two-tailed Student's t test. Puncta bin size is 15 μm².

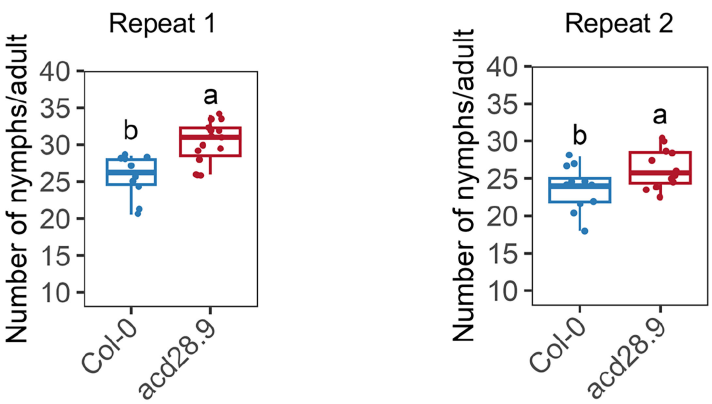

Fig. S27. ***M. persicae*** **fecundity is increased on** ***A. thaliana acd28.9*** **mutant plants.** The graphs display data from two independent fecundity assays in addition to the one shown in Fig 5G. Significance assessed by two-tailed Student's t test.

Table S1. List of CathB genes identified in the v2 genome assembly together with the Gene IDs from annotation versions 2.0 (*64*) and v2.1 (*13*) of the potato-peach aphid *Myzus persicae* clone O. DE, differentially expressed CathB genes in the *M. persicae* colonies reared on 8 divergent plant species (*Arabidopsis thaliana*, *Nicotiana benthamiana*, *Solanum tuberosum*, *Pisum sativum*, *Phaseolus vulgaris*, *Helianthus annuus*, *Chrysanthemum indicum* and *Zea mays*), compared to the ones reared on *Brassica rapa* (*10*).

Name	v2.0 Gene ID	v2.1 Gene ID	Expression on different plants	Note
CathB1	g2983.t1	MYZPE13164_O_EIv2.1_0034760	-	
CathB2	g2984.t2	MYZPE13164_O_EIv2.1_0034770	-	
CathB3	g7792.t1	MYZPE13164_O_EIv2.1_0100520	DE	
CathB4	g2985.t1	MYZPE13164_O_EIv2.1_0034780	-	
CathB6	g8486.t1	MYZPE13164_O_EIv2.1_0111090	DE	
CathB7	g6427.t1	MYZPE13164_O_EIv2.1_0083010	-	
CathB8	g8482.t1	MYZPE13164_O_EIv2.1_0111040	-	
CathB9	g8488.t1	MYZPE13164_O_EIv2.1_0111110	-	
CathB10	g8483.t1	MYZPE13164_O_EIv2.1_0111050	DE	
CathB11	g8484.t1	MYZPE13164_O_EIv2.1_0111060	-	
CathB12	g8485.t1	MYZPE13164_O_EIv2.1_0111080	DE	
CathB13	g8487.t1	MYZPE13164_O_EIv2.1_0111100	-	
CathB14	g8489.t1	MYZPE13164_O_EIv2.1_0111120	DE	
CathB15	g8490.t1	MYZPE13164_O_EIv2.1_0111130	DE	
CathB16	g27805.t1	MYZPE13164_O_EIv2.1_0124680	-	Truncated
CathB17	g27806.t1	MYZPE13164_O_EIv2.1_0124690	DE	
CathB18	g27807.t1	MYZPE13164_O_EIv2.1_0124700	DE	
CathB19	g24530.t1	MYZPE13164_O_EIv2.1_0159270	DE	
CathB20	g24532.t1	MYZPE13164_O_EIv2.1_0159280	DE	
CathB21	g25994.t1	MYZPE13164_O_EIv2.1_0179580	-	
CathB22	g26516.t1	MYZPE13164_O_EIv2.1_0186720	-	
CathB23	g9913.t2	MYZPE13164_O_EIv2.1_0269540	DE	
CathB24	g10179.t1	MYZPE13164_O_EIv2.1_0272820	-	
CathB25	g12475.t1	MYZPE13164_O_EIv2.1_0302340	-	
CathB26	g12476.t1	MYZPE13164_O_EIv2.1_0302350	-	
CathB27	g12481.t1	MYZPE13164_O_EIv2.1_0302390	-	
CathB28	g12482.t1	MYZPE13164_O_EIv2.1_0302400	-	

Table S2. Peptides of CathB proteins detected in *M. persicae* Clone O oral secretion (OS) by MS. Gene ID and annotation ID of *M. persicae* clone O v2.1 database are labelled.

Name	Gene ID	Transcript ID	No. of Peptides	Peptides
CathB3	g7792.t1	MYZPE13164_O_EIv2.1_0100520	5	HGLVTGGDYK
				DQGNCGSCWAVATSSAFADR
				DYYYLTYGSIQK
				SGEGCEPYR
				DVMTYGPIEASFDVYDDFPSYK
CathB6	g8486.t1	MYZPE13164_O_EIv2.1_0111090	8	KGLVTGGDYK
				GLVTGGDYK
				MCYGDQDLDFDEDHR
				DQGNCGSCWAVATSSAFADR
				DYYYLTYGSIQK
				SGEGCEPYR
				DVMTYGPIEASFDVYDDFPSYK
				VRDQGNCGSCWAVATSSAFADR
CathB12	g8485.t1	MYZPE13164_O_EIv2.1_0111080	8	KGLVTGGDYK
				GLVTGGDYK
				MCYGDQDLDFDEDHR
				DQGNCGSCWAVATSSAFADR
				DYYYLTYGSIQK
				SGEGCEPYR
				DVMTYGPIEASFDVYDDFPSYK
				VRDQGNCGSCWAVATSSAFADR
CathB14	g8489.t1	MYZPE13164_O_EIv2.1_0111120	5	DQGNCGSCWAVATSSAFADR
				DYYYLTYGSIQK
				VRDQGNCGSCWAVATSSAFADR
				WVYCDTIGR
				FSTHGLVTGGDYNSGEGCEPYR
CathB17	g27806.t1	MYZPE13164_O_EIv2.1_0124690	5	DQGNCGSCWAVATSSAFADR
				SGEGCEPYR
				MCYGNQDLDFDQDHR
				DYYYLTYGSIQK
				DVMTYGPIEASFDVYDDFPSYK

CathB18	g27807.t1	MYZPE13164_O_EIv2.1_0124700	5	DQGNCGSCWAVATSSAFADR<br>SGEGCEPYR<br>MCYGNQDLDFDQDHR<br>DYYYLTYGSIQK<br>DVMTYGPIEASFDVYDDFPSYK
CathB19	g24530.t1	MYZPE13164_O_EIv2.1_0159270	5	SGEGCEPYR<br>DVMTYGPIEASFDVYDDFPSYK<br>SHGLVTGGDYK<br>DSYYLTYGSIQK<br>DQGNCGSCWALATSSAFADR
CathB20	g24532.t1	MYZPE13164_O_EIv2.1_0159280	4	SGEGCEPYR<br>SHGLVTGGDYK<br>DSYYLTYGSIQK<br>DQGNCGSCWALATSSAFADR

Table S3. Markers of cellular components used in the study.

Constructs	Protein name	Cellular components	References
VCSc-mCherry	Varicose, VCS	Processing bodies	(*16*)
YFP-VCSc	Varicose, VCS	Processing bodies	(*18*)
DCP1-RFP	Decapping protein 1, DCP1	Processing bodies	(*16*)
YFP-DCP1	Decapping protein 1, DCP1	Processing bodies	(*17*)
DCP5-mCherry	Decapping protein 5, DCP5	Processing bodies	(*26*)
RBP47b-mCherry	RNA binding protein 47b, RBP47b	Stress granule	(*19*)
RFP-ATG8	Autophagy-related protein 8, ATG8	Autophagosome	(*21*)
NPR1-mCherry	Nonexpressor of pathogenesis related 1, NPR1	NPR1 condensates	(*22*)

Table S4. Peptides corresponding to GFP (GFP-TurboID-FLAG) or CathB6 (GFP-TurboID-FLAG) detected in the TurboID-based proximity samples by mass spectrometry. Amino acids corresponding to GFP and CathB6 sequences within the GFP-TurboID-FLAG and CathB6-TurboID-FLAG sequences are underlined.

	**Protein sequences**	**No. of peptides**	**Peptide sequences**
GFP-TurboID-FLAG	MVSKGEELFTGVVPILVELDGDVNGHKFSVS<br>GEGEGDATYGKLTLKFICTTGKLPVPWPTLV<br>TTLTYGVQCFSRYPDHMKQHDFFKSAMPEG<br>YVQERTIFFKDDGNYKTRAEVKFEGDTLVNR<br>IELKGIDFKEDGNILGHKLEYNYNSHNVYIMA<br>DKQKNGIKVNFKIRHNIEDGSVQLADHYQQN<br>TPIGDGPVLLPDNHYLSTQSALSKDPNEKRD<br>HMVLLEFVTAAGITLGMDELYKTSMASMTG<br>GQQMGRGSEFMKDNTVPLKLIALLANGEFH<br>SGEQLGETLGMSRAAINKHIQTLRDWGVDV<br>FTVPGKGYSLPEPIPLLNAKQILGQLDGGSV<br>AVLPVVDSTNQYLLDRIGELKSGDACIAEYQ<br>QAGRGSRGRKWFSPFGANLYLSMFWRLKR<br>GPAAIGLGPVIGIVMAEALRKLGADKVRVKW<br>PNDLYLQDRKLAGILVELAGITGDAAQIVIGA<br>GINVAMRRVEESVVNQGWITLQEAGINLDR<br>NTLAATLIRELRAALELFEQEGLAPYLPRWE<br>KLDNFINRPVKLIIGDKEIFGISRGIDKQGALL<br>LEQDGVIKPWMGGEISLRSAEKKLAAALED<br>YKDHDGDYKDHDLDAAAADYKDDDDK	19	DDGNYKTR<br>FICTTGK<br>SAMPEGYVQER<br>EDGNILGHK<br>YPDHMKQHDFFK<br>FSVSGEGEGDATYGK<br>NGIKVNFKIR<br>FEGDTLVNR<br>TIFFKDDGNYKTR<br>TRAEVKFEGDTLVNR<br>TIFFKDDGNYK<br>LEYNYNSHNVYIMADK<br>AEVKFEGDTLVNR<br>LEYNYNSHNVYIMADKQK<br>FEGDTLVNRIELK<br>GIDFKEDGNILGHK<br>FSVSGEGEGDATYGKLTLK<br>AEVKFEGDTLVNRIELK<br>GEELFTGVVPILVELDGDVNGHK
CathB6-TurboID-FLAG	MRGVQIPNKNNMNLYKSEDADYDNTYIPRF<br>FDARRKWRHCSTIGRVRDQGNCGSCWAV<br>ATSSAFADRLCVATNADFNELLSAEEITFCC<br>HTCGFGCNGGYPIKAWKRFSKKGLVTGGD<br>YKSGEGCEPYRVPPCPNDDQGNNTCAGKP<br>MESNHRCTRMCYGDQDLDFDEDHRYTRD<br>YYYLTYGSIQKDVMTYGPIEASFDVYDDFP<br>SYKSGVYYVKSENASYLGGHAVKLIGWGEE	20	KGLVTGGDYK<br>MRGVQIPNK<br>SENASYLGGHAVK<br>GVQIPNK<br>GLVTGGDYK<br>GLVTGGDYKSGEGCEPYR

	YGVPYWLMVNSWNEDWGDNGFFKIQRGT<br>NECGVDNSTTAGVPVTNTSMASMTGGQQ<br>MGRGSEFMKDNTVPLKLIALLANGEFHSG<br>EQLGETLGMSRAAINKHIQTLRDWGVDVF<br>TVPGKGYSLPEPIPLLNAKQILGQLDGGSV<br>AVLPVVDSTNQYLLDRIGELKSGDACIAEY<br>QQAGRGSRGRKWFSPFGANLYLSMFWR<br>LKRGPAAIGLGPVIGIVMAEALRKLGADKV<br>RVKWPNDLYLQDRKLAGILVELAGITGDA<br>AQIVIGAGINVAMRRVEESVVNQGWITLQ<br>EAGINLDRNTLAATLIRELRAALELFEQEG<br>LAPYLPRWEKLDNFINRPVKLIIGDKEIFGI<br>SRGIDKQGALLLEQDGVIKPWMGGEISLR<br>SAEKKLAAALEDYKDHDGDYKDHDLDAA<br>AADYKDDDDK	MCYGDQDLDFDEDHR<br>MCYGDQDLDFDEDHRYTR<br>DYYYLTYGSIQK<br>DVMTYGPIEASFDVYDDFPSYK<br>HCSTIGRVR<br>SEDADYDNTYIPR<br>VRDQGNCGSCWAVATSSAFADR<br>DQGNCGSCWAVATSSAFADR<br>SGEGCEPYR<br>GVQIPNKNNMNLYK<br>FSKKGLVTGGDYK<br>SGVYVKSENASYLGGHAVK<br>HCSTIGR<br>VPPCPNDDQGNNTCAGKPMESNHR

Table S5. Plasmids used in the study.

Plasmid name	Purpose	Ref.
pBI121-CathB3	Generating stable transgenic *A. thaliana* plants of His-CathB3	This study
pBI121-CathB3^{C116D}	Generating stable transgenic *A. thaliana* plants of His-CathB3^{C116D}	This study
pBI121-CathB6	Generating stable transgenic *A. thaliana* plants of His-CathB6	This study
pBI121-CathB6^{C114D}	Generating stable transgenic *A. thaliana* plants of His-CathB6^{C114D}	This study
pBI121-CathB9	Generating stable transgenic *A. thaliana* plants of His-CathB9	This study
pB7FWG2-ATG	Generating stable transgenic *A. thaliana* plants of GFP, Transient expression of GFP in *A. thaliana* protoplast and *N. benthamiana*	This study
pB7RWG2-ATG	Transient expression of RFP in *A. thaliana* protoplast and *N. benthamiana*	This study
pB7WG2-mCherry	Transient expression of mCherry in *N. benthamiana*	This study
pB7FWG2-CathB3	Transient expression of CathB3-GFP in *N. benthamiana*	This study
pB7FWG2-CathB6	Generating stable transgenic *A. thaliana* plants of CathB6-GFP, Transient expression of CathB6-GFP in *A. thaliana* protoplast and *N. benthamiana*	This study
pB7FWG2-CathB6^{C114D}	Transient expression of CathB6^{C114D}-GFP in *N. benthamiana*	This study
pB7FWG2-CathB6$^{\Delta 266\text{-}333}$	Transient expression of CathB6$^{\Delta 266\text{-}333}$-GFP in *N. benthamiana*	This study
pB7RWG2-CathB6	Transient expression of CathB6-RFP in *A. thaliana* protoplast and *N. benthamiana*	This study
pB7FWG2-CathB9	Generating stable transgenic *A. thaliana* plants of CathB9-GFP, Transient expression of CathB9-GFP in *N. benthamiana*	This study
pB7WG2-VCS-mCherry	Transient expression of VCS-mCherry in *N. benthamiana*	This study
pH7YWG2-YFP-VCS[1]	Transient expression of YFP-VCS in *N. benthamiana*	(*18*)
pB7RWG2-DCP1[2]	Transient expression of DCP1-RFP in *N. benthamiana*	(*77*)
pABM043-YFP-DCP1[2]	Transient expression of YFP-DCP1 in *A. thaliana* protoplast and *N. benthamiana*	(*78*)
pB7WG2-DCP5-mCherry	Transient expression in *N. benthamiana*	This study

pK7-CathB6-BFP	Transient expression of CathB6-BFP in *N. benthamiana*	This study
pK7-VCS-BFP	Transient expression of VCS-BFP in *N. benthamiana*	This study
pB7WG2-Acd28.9-mCherry	Transient expression of Acd28.9-mCherry in *N. benthamiana*	This study
pB7WG2-GFP-TurboID	Generating stable transgenic plants of GFP-TurboID-FLAG	This study
pB7WG2-CathB6-TurboID	Generating stable transgenic plants of CathB6-TurboID-FLAG	This study
pB7WG2-EDS1-mCherry	Transient expression of EDS1-mCherry in *A. thaliana* protoplast and *N. benthamiana*	This study
pB7WG2-PAD4-mCherry	Transient expression of PAD4-mCherry in *N. benthamiana*	This study
pB7WG2-SAG101-mCherry	Transient expression of SAG101-mCherry in *N. benthamiana*	This study
pB7WG2-ADR1-L1-mCherry[3]	Transient expression of ADR1-L1-mCherry in *N. benthamiana*	(*79*)
pB7FWG2-EDS1	Transient expression of EDS1-GFP in *N. benthamiana*	This study
pB7WG2-RBP47b-mCherry	Transient expression of RBP47b-mCherry in *N. benthamiana*	This study
pB7WG2-RFP-ATG8	Transient expression of RFP-ATG8 in *N. benthamiana*	(*80*)
pB7WG2-NPR1-mCherry	Transient expression of NPR1-mCherry in *N. benthamiana*	This study
pB7WG2-HA-CathB6	Transient expression of HA-CathB6 in *N. benthamiana*	This study
pB7WG2-HA-CathB6$^{\Delta 266\text{-}333}$	Transient expression of HA-CathB6$^{\Delta 266\text{-}333}$ in *N. benthamiana*	This study
pB7WG2-HA-Acd28.9	Transient expression of HA-Acd28.9 in *N. benthamiana*	This study
pB7WG2-HA-EDS1	Transient expression of HA-EDS1 in *N. benthamiana*	This study
pB7WG2-Flag-PAD4	Transient expression of Flag-PAD4 in *N. benthamiana*	This study
pDESTGBKT7	Y2H Empty vector of BD	This study
pDESTGADT7	Y2H Empty vector of AD	This study
pDESTGBKT7-CathB3	Y2H analysis of BD-CathB3	This study
pDESTGBKT7-CathB6	Y2H analysis of BD-CathB6	This study
pDESTGBKT7-CathB6^{C114D}	Y2H analysis of BD-CathB6^{C114D}	This study
pDESTGBKT7-CathB6^{C114A}	Y2H analysis of BD-CathB6^{C114A}	This study
pDESTGBKT7-CathB9	Y2H analysis of BD-CathB9	This study
pDESTGBKT7-AtCathB3	Y2H analysis of BD-AtCathB3	This study

pDESTGBKT7-AtCathB3^{C113A}	Y2H analysis of BD-AtCathB3^{C113A}	This study
pDESTGBKT7-EDS1	Y2H analysis of BD-EDS1	This study
pDESTGBKT7-Acd28.9	Y2H analysis of BD-Acd28.9	This study
pDESTGADT7-CathB6	Y2H analysis of AD-CathB6	This study
pDESTGADT7-CathB6^{C114A}	Y2H analysis of AD-CathB6^{C114A}	This study
pDESTGADT7-AtCathB3	Y2H analysis of AD-AtCathB3	This study
pDESTGADT7-AtCathB3^{C113A}	Y2H analysis of AD-AtCathB3^{C113A}	This study
pDESTGADT7-EDS1	Y2H analysis of AD-EDS1	This study
pDESTGADT7-EDS1$^{1-384}$	Y2H analysis of AD-EDS1$^{1-384}$	This study
pDESTGADT7-EDS1$^{385-623}$	Y2H analysis of AD-EDS1$^{385-623}$	This study
pDESTGADT7-EDS1$^{385-444}$	Y2H analysis of AD-EDS1$^{385-444}$	This study
pDESTGADT7-EDS1$^{445-504}$	Y2H analysis of AD-EDS1$^{445-504}$	This study
pDESTGADT7-EDS1$^{505-564}$	Y2H analysis of AD-EDS1$^{505-564}$	This study
pDESTGADT7-EDS1$^{565-623}$	Y2H analysis of AD-EDS1$^{565-623}$	This study
pDESTGADT7-EDS1$^{405-554}$	Y2H analysis of AD-EDS1$^{405-554}$	This study
pDESTGADT7-PAD4	Y2H analysis of AD-PAD4	This study
pDESTGADT7-SAG101	Y2H analysis of AD-SAG101	This study
pFastBac-HTB-GFP	Expression of GFP in sf9 cells using baculovirus system	This study
pFastBac-HTB-CathB6-GFP	Expression of CathB6-GFP in sf9 cells using baculovirus system	This study
pFastBac-HTB-HA-EDS1	Expression of HA-EDS1 in sf9 cells using baculovirus system	This study

Kindly donated by [1]Sophien Kamoun, [2]Suayib Üstün, [3]Jonathan Jones and [4]Tolga Bozkurt.

Table S6. *A. thaliana* lines used in the study.

***A. thaliana* line**	**Plasmids for transformation**	**Ref.**
Col-0_His-CathB3	pBI121-CathB3	This study
Col-0_His-CathB3^{C116D}	pBI121-CathB3^{C116D}	This study
Col-0_His-CathB6	pBI121-CathB6	This study
Col-0_His-CathB6^{C114D}	pBI121-CathB6^{C114D}	This study
Col-0_His-CathB9	pBI121-CathB9	This study
Col-0_GFP	pB7FWG2-ATG	This study
Col-0_CathB6-GFP	pB7FWG2-CathB6	This study
Col-0_CathB9-GFP	pB7FWG2-CathB9	This study
Col-0_GFP-TurboID	pB7WG2-GFP-TurboID-FLAG	This study
Col-0_CathB6-TurboID	pB7WG2-CathB6-TurboID-FLAG	This study
eds1-2[1]	–	(*81*)
eds1-2_GFP	pB7FWG2-ATG	This study
eds1-2_CathB6-GFP	pB7FWG2-CathB6	This study
pad4-1[1]	–	(*82*)
*pad4-1*_GFP	pB7FWG2-ATG	This study
*pad4-1*_CathB6-GFP	pB7FWG2-CathB6	This study
acd28.9	–	NASC Arabidopsis stock (SALK_002031C) (*83*)

[1]Kindly donated by Jonathan Jones.

Table S7. qRT-PCR primers used in the study.

Primer name	Primer sequence (5' -> 3')
GFP-qPCR-F	GCAAGCTGACCCTGAAGTTC
GFP-qPCR-R	GCTTCATGTGGTCGGGGTA
B3-qPCR-F2	ATCCGATAAAAGCGTGGGAAC
B3-qPCR-R2	TGCCCTGTTCGTCATAAGGA
B6-qPCR-F2	ACAAATCTGGAGAGGGTTGTGA
B6-qPCR-R2	AGCACATCCTGGTACACCTG
CathB9-qPCR-F	GTTGTCAACGAGCTCAGCTT
CathB9-qPCR-R	GAACATCCATCACCACAGTTGT
EF1alpha_qPCR_F	CAGGCTGATTGTGCTGTTCTTA
EF1alpha_qPCR_R	GTTGTATCCGACCTTCTTCAGG
PAD4_qPCR_F	TGGTCGACGCTGCCATACT
PAD4_qPCR_R	GGTTGAATGGCCGGTTATCA
SAG101_qPCR_F	CATTCCTCTGCTCCGAGAAC
SAG101_qPCR_R	CGTTTTAACGTCGGTTCGAT
PR1_qPCR_F	ATACACTCTGGTGGGCCTTACG
PR1_qPCR_R	TACACCTCACTTTGGCACATCC
ICS1_qPCR_F	TCCGTGACCTTGATCCTTTC
ICS1_qPCR_R	ACAGCGATCTTGCCATTAGG
PBS3_qPCR_F	TTCGCTGGCTTGTATAGGATGA
PBS3_qPCR_R	CTGGAAATGTTGAGGTGTCAGC
EDS5_qPCR_F	ACCTTTCTTCATGGCGTTGTCT
EDS5_qPCR_R	ATTGAAATCCGACGAGAACGA
CBP60g_qPCR_F	AAGAAGAATTGTCCGAGAGGAG
CBP60g_qPCR_R	GGCGAGTTTATGAAGCACAG
SARD1_qPCR_F	CCGATATGCGAAGTTATGAAAGC
SARD1_qPCR_R	AGTGGCTCGCAGCATATTGTT
FMO1_qPCR_F	TGTGTTTGAAGATGGGACGACA
FMO1_qPCR_R	GTTCGAGCTGCTTTGGACGTAT

Movie S1. *M. persicae* CathB6 locates to mobile puncta within cells of *N. benthamiana* leaves. The white arrow shows an example of a puncta fusion. Scale bar, 30 µm.

Raw blots for fig. S7

fig. S7A

1. Nb wild-type
2. GFP + mCherry
3. CathB6-GFP + mCherry

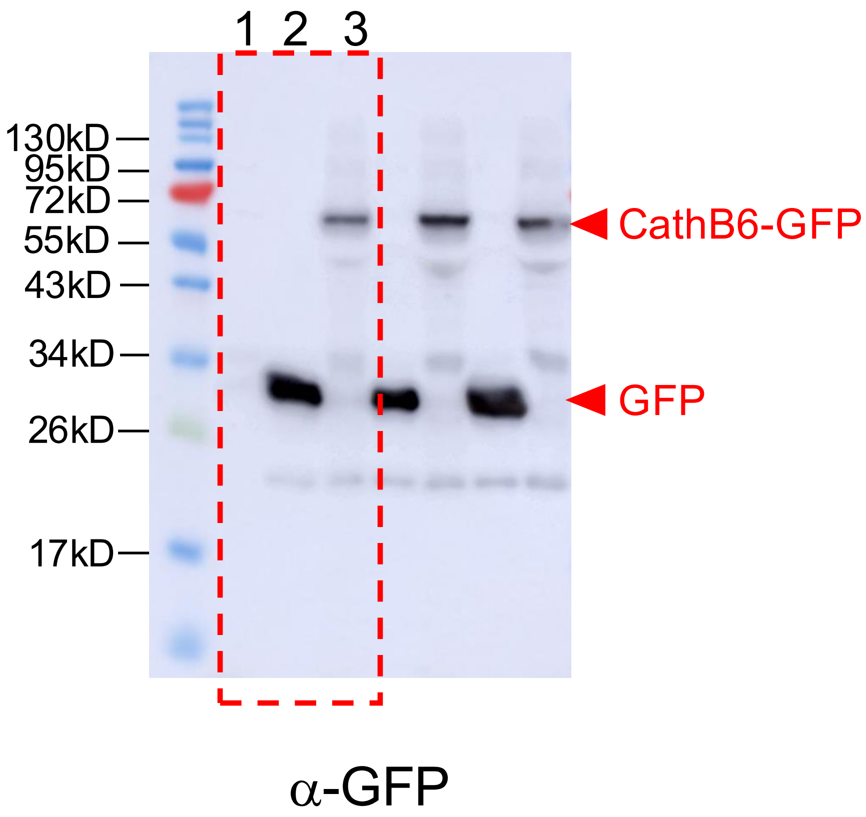

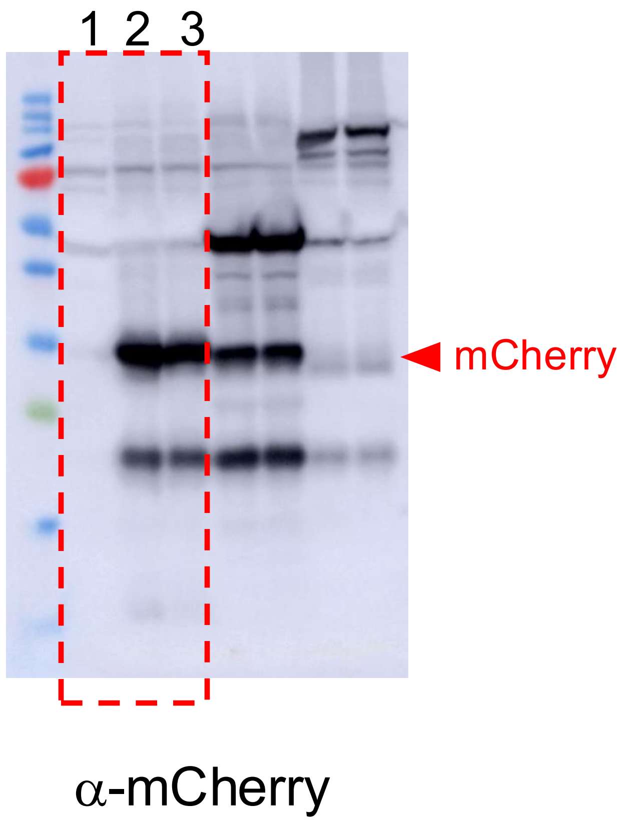

fig. S7B

1. GFP + VCS-mCherry
2. CathB6-GFP + VCS-mCherry
3. GFP + DCP5-mCherry
4. CathB6-GFP + DCP5-mCherry

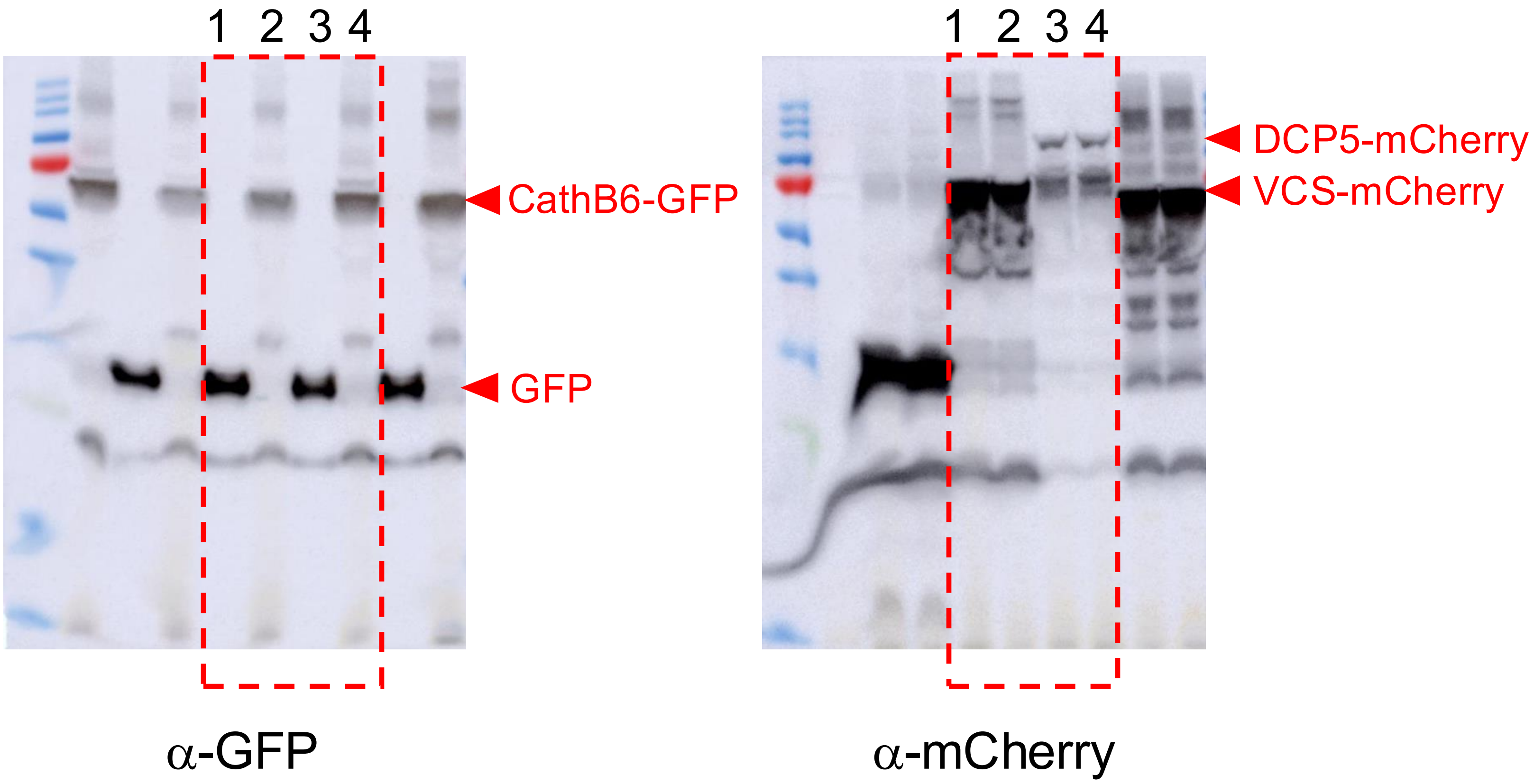

fig. S7C

1. GFP + EDS1-mCherry
2. CathB6-GFP + EDS1-mCherry

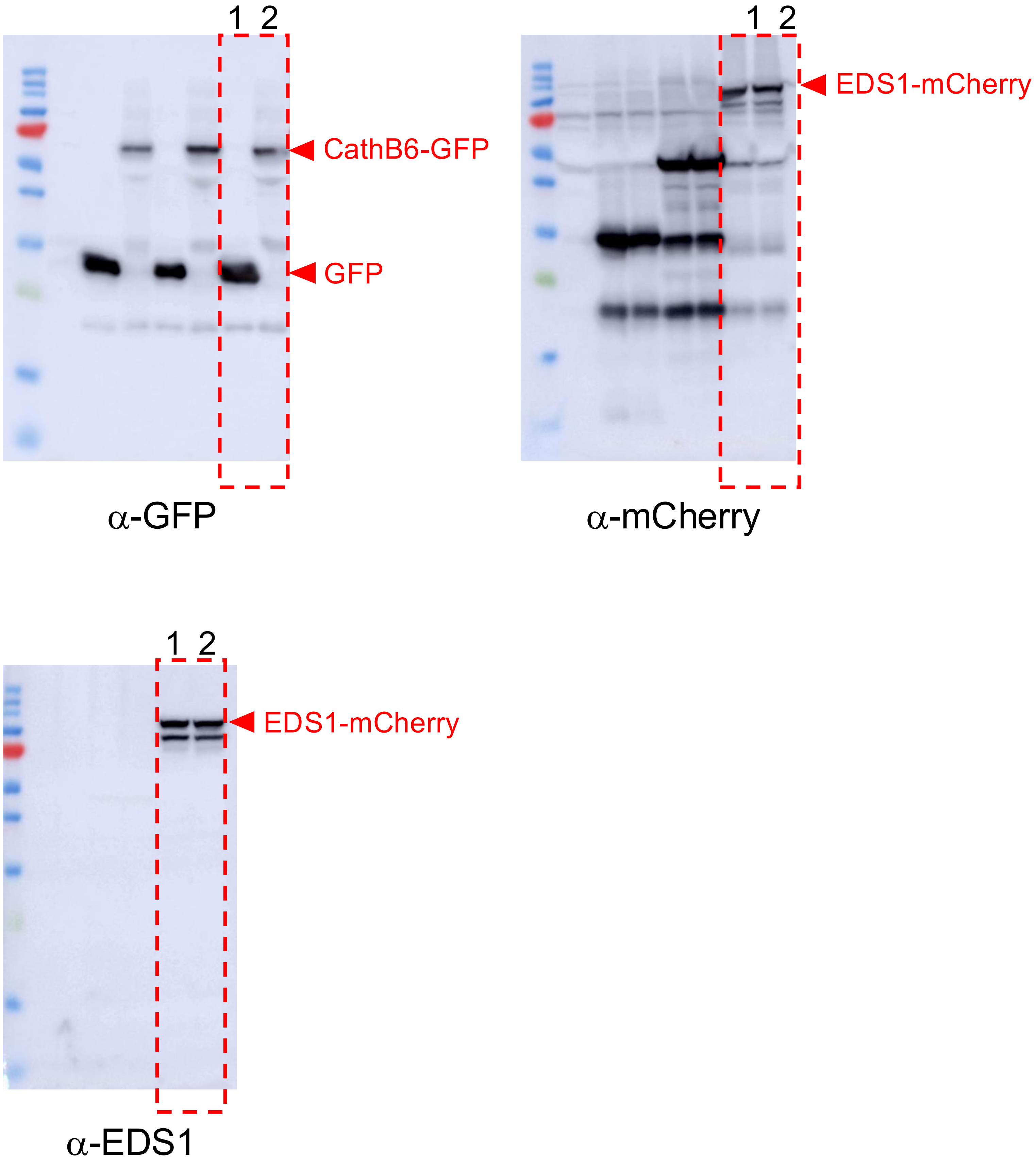

fig. S7D

1. GFP + mCherry
2. GFP + mCherry + HA-CathB6
3. EDS1-GFP + mCherry
4. EDS1-GFP+ mCherry + HA-CathB6

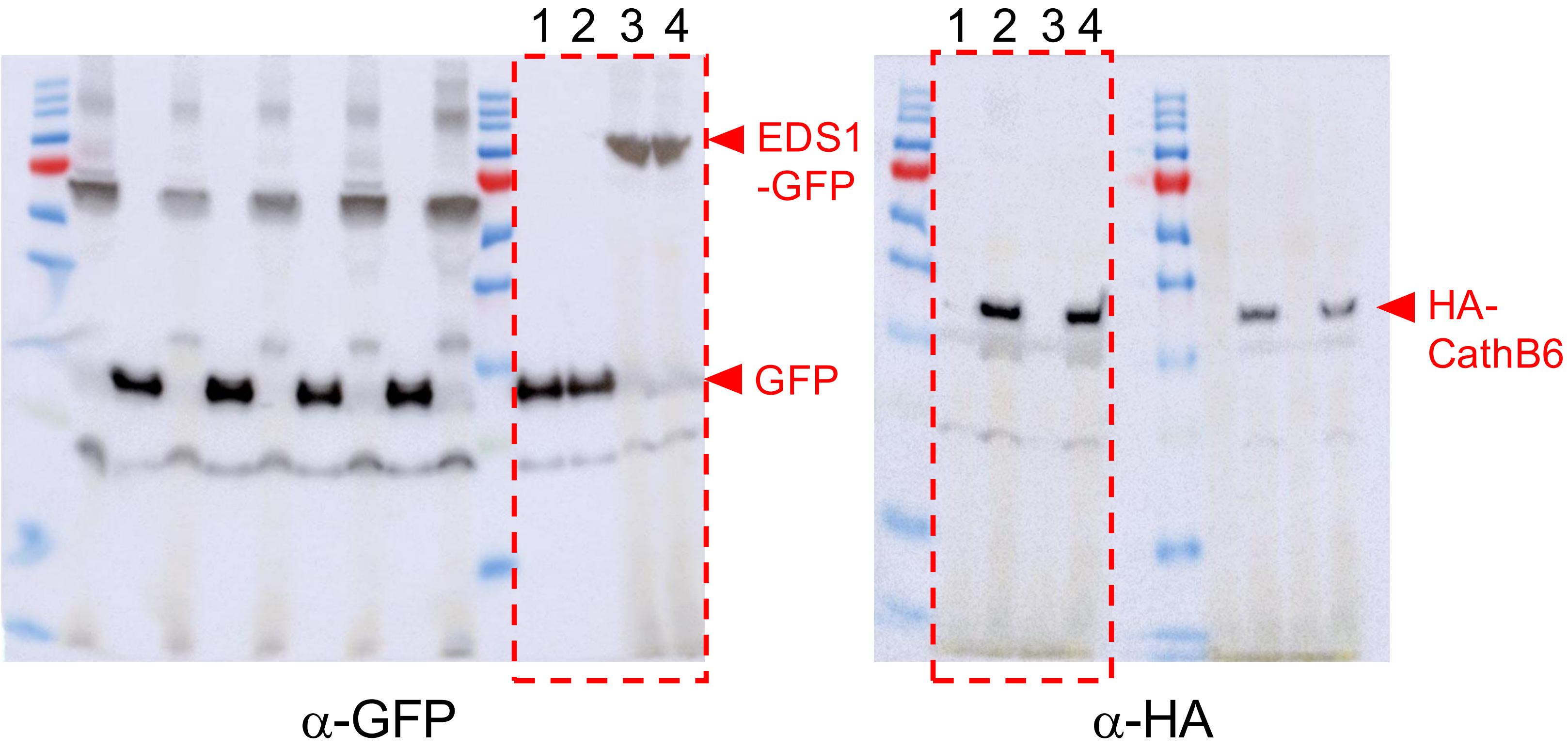

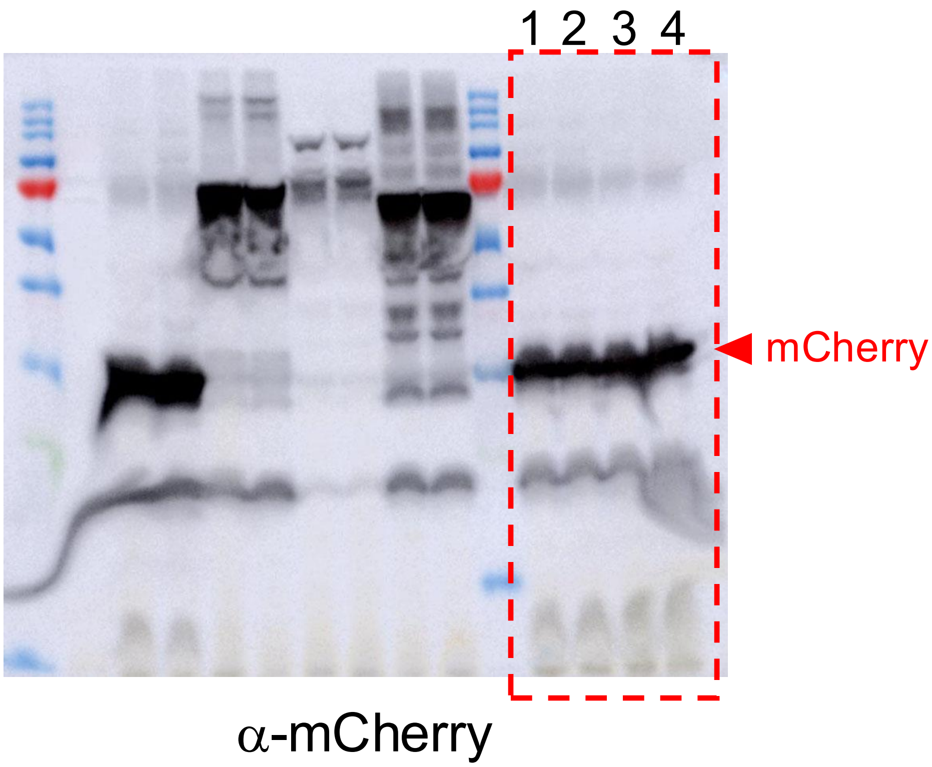

fig. S7E

1. EDS1-GFP + VCS-mCherry
2. EDS1-GFP + VCS-mCherry + HA-CathB6

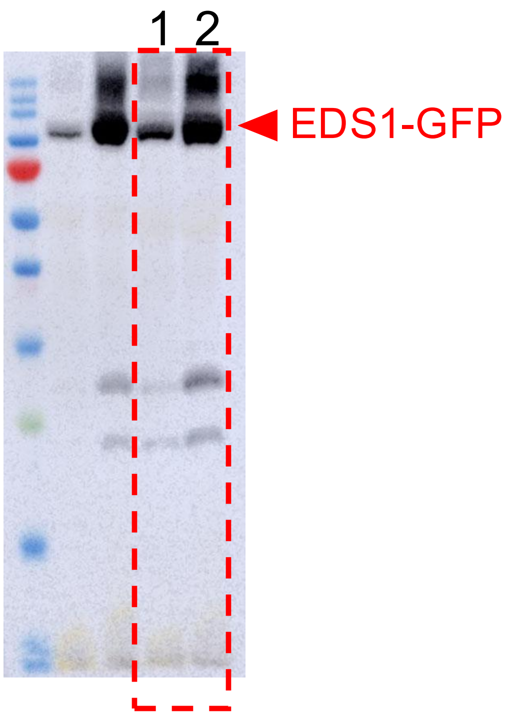

α-GFP

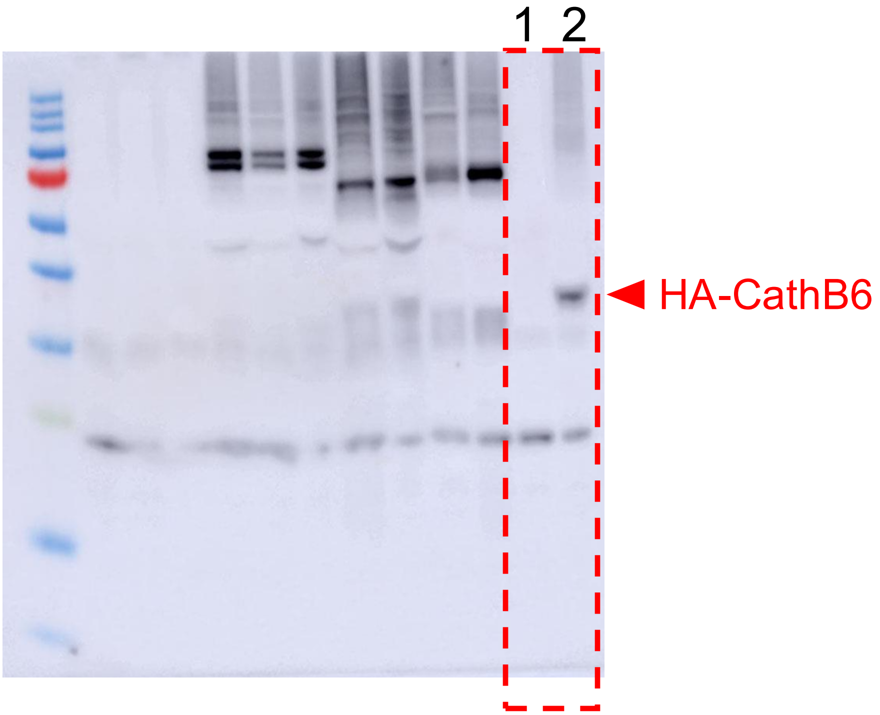

α-HA

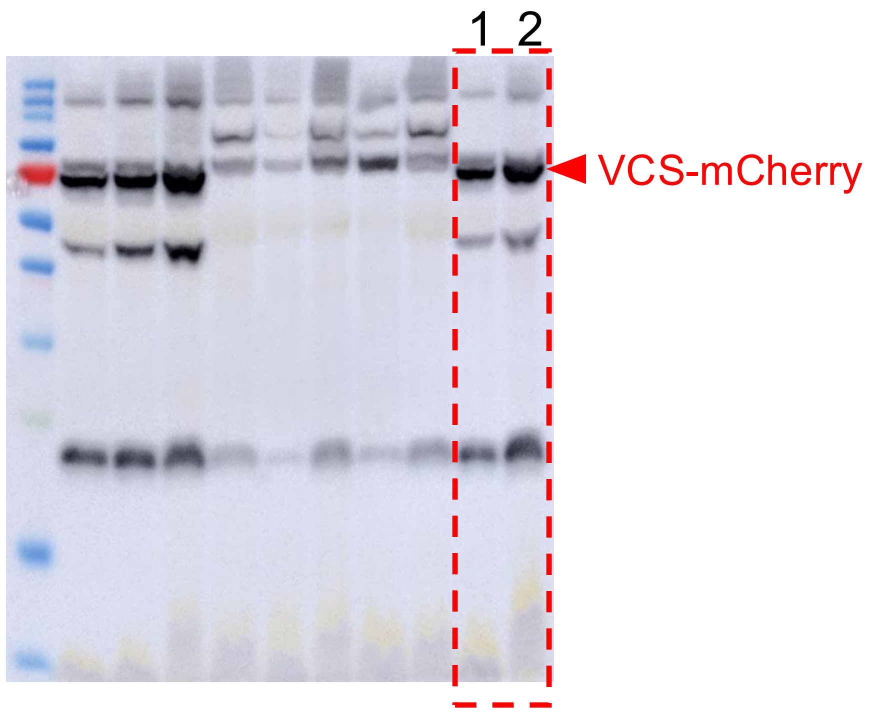

α-mCherry

fig. S7F

1. EDS1-GFP + PAD4-mCherry
2. EDS1-GFP + PAD4-mCherry + HA-CathB6
3. EDS1-GFP + SAG101-mCherry
4. EDS1-GFP + SAG101-mCherry + HA-CathB6

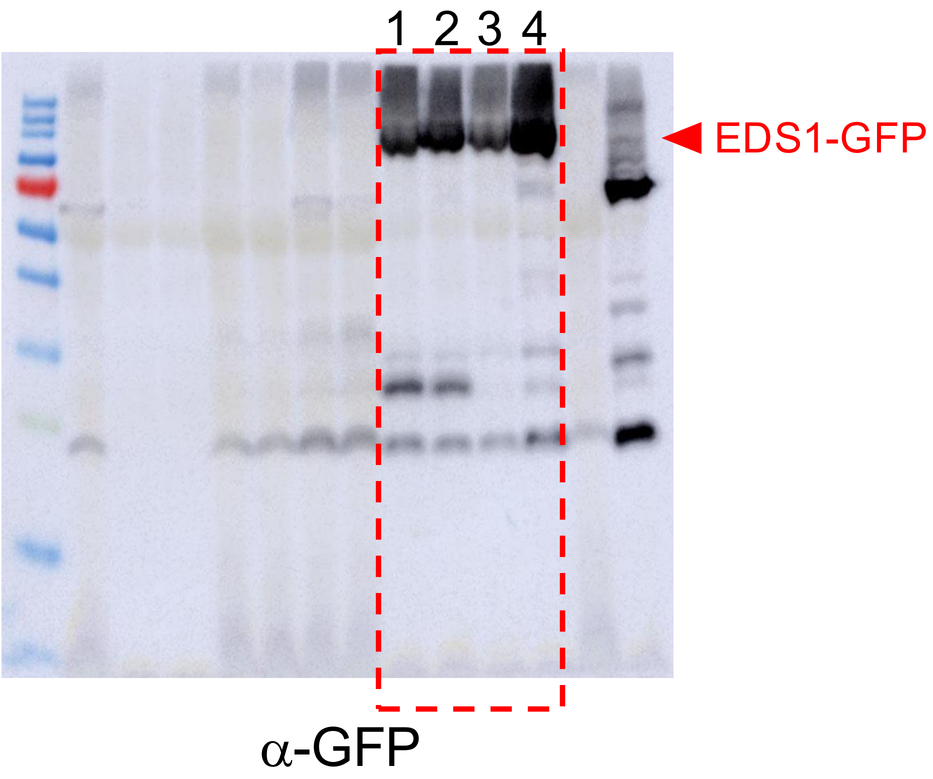

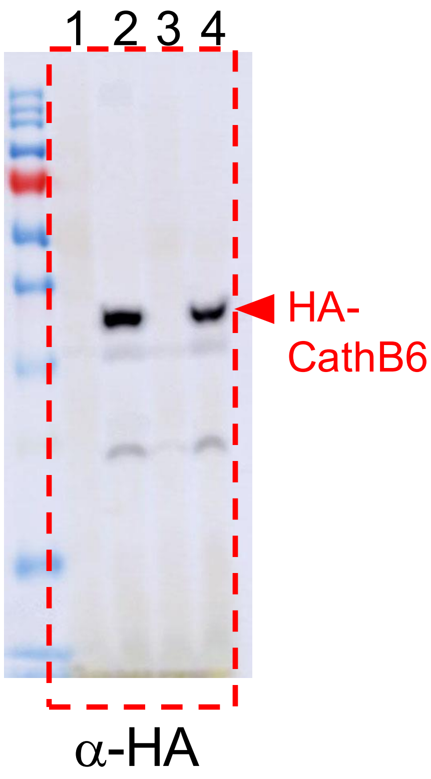

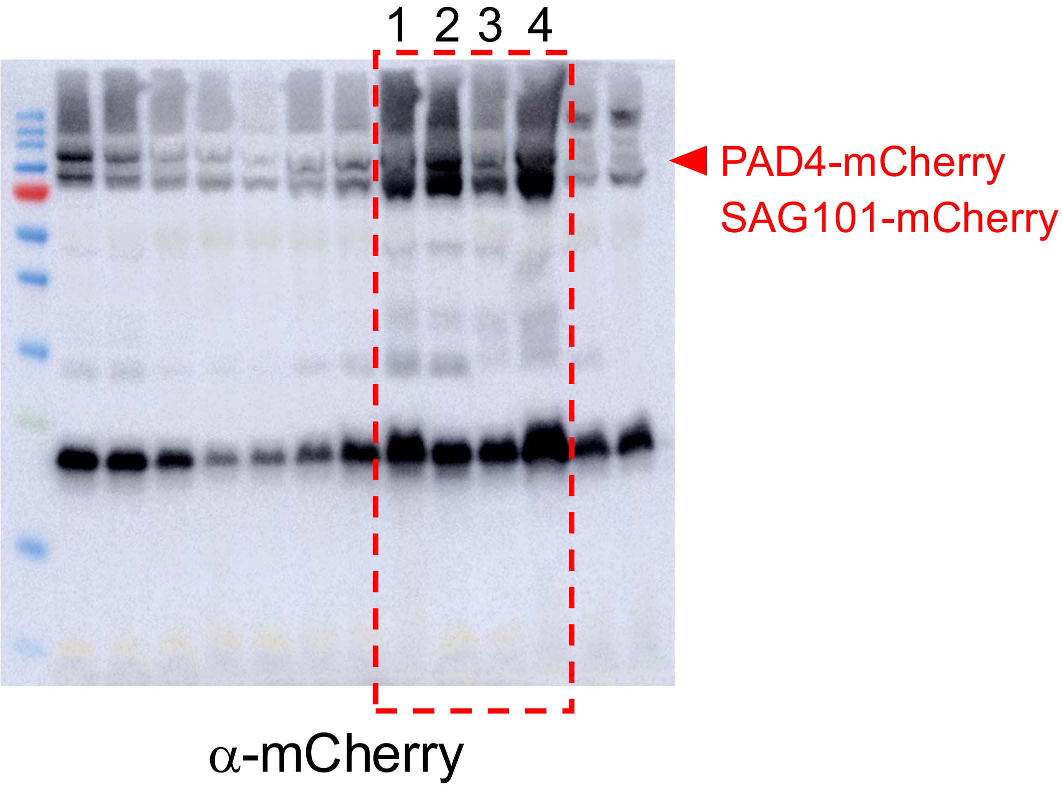

fig. S7G

1. EDS1-GFP + ADR1-L1-mCherry
2. EDS1-GFP + Flag-PAD4 + ADR1-L1-mCherry
3. EDS1-GFP + Flag-PAD4 + ADR1-L1-mCherry + HA-CathB6

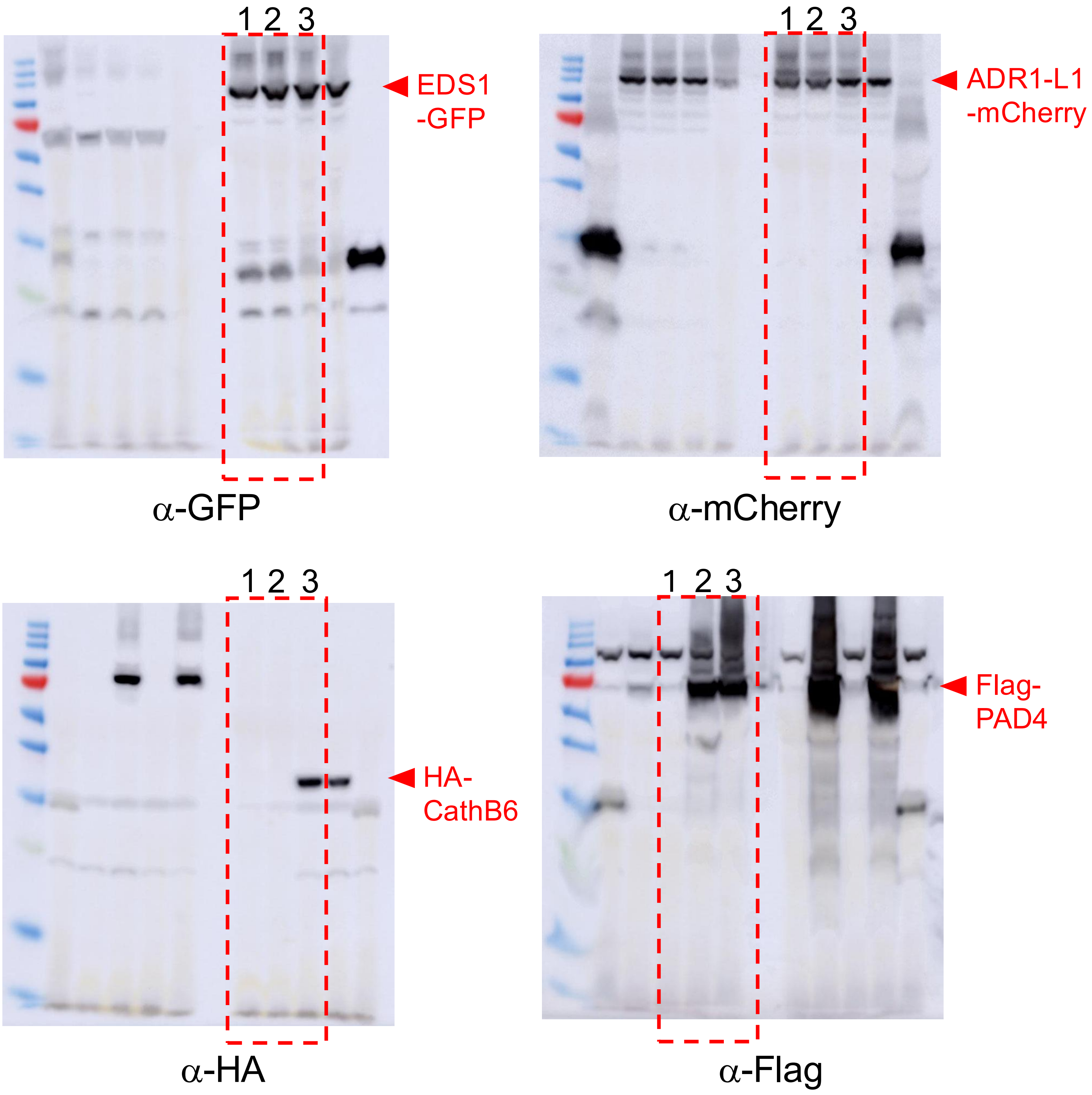

fig. S7H

1. GFP + Acd28.9-mCherry
2. CathB6-GFP + Acd28.9-mCherry

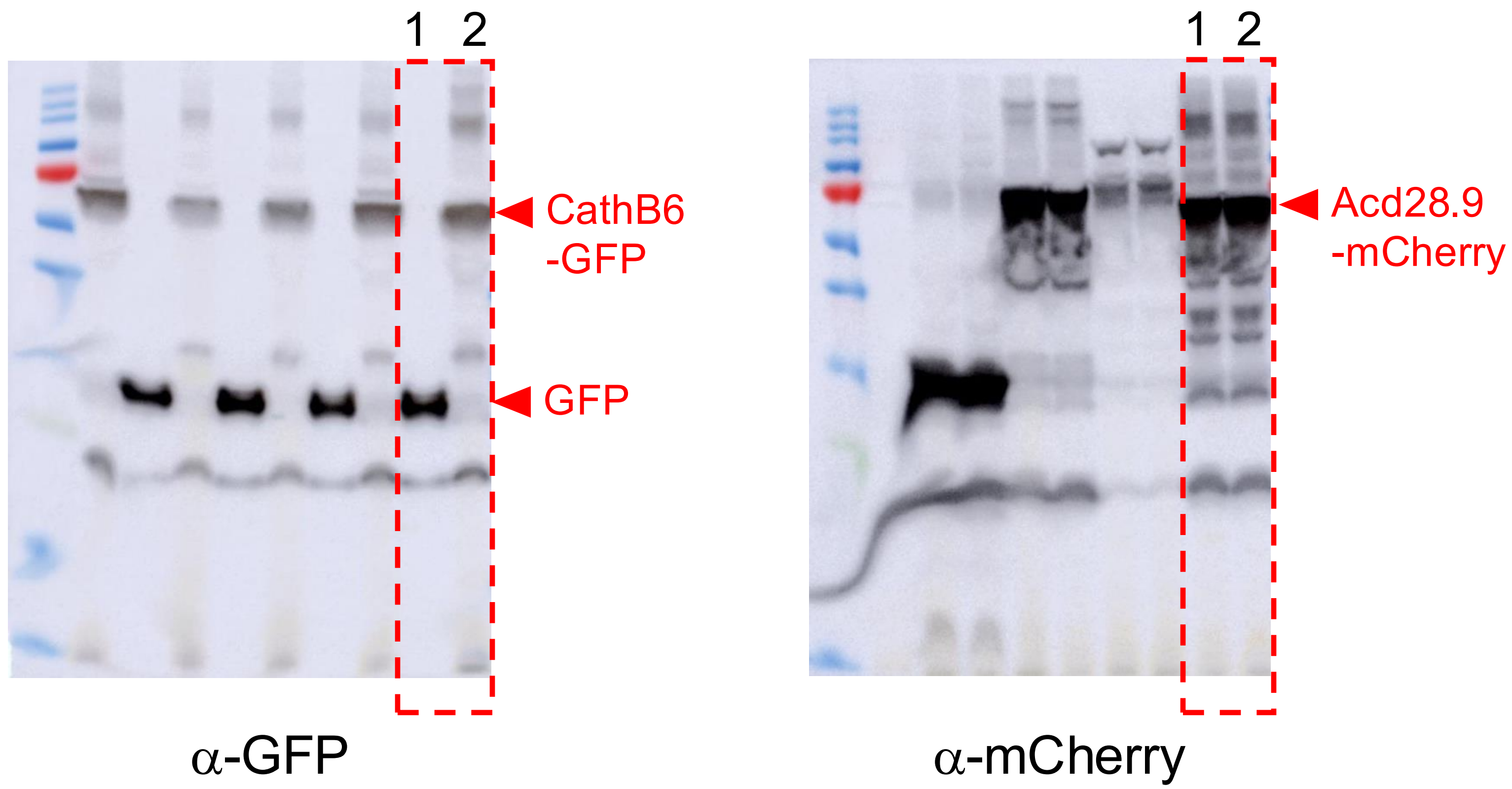

fig. S7I

1. EDS1-GFP + Acd28.9-mCherry
2. EDS1-GFP+ Acd28.9-mCherry + HA-CathB6

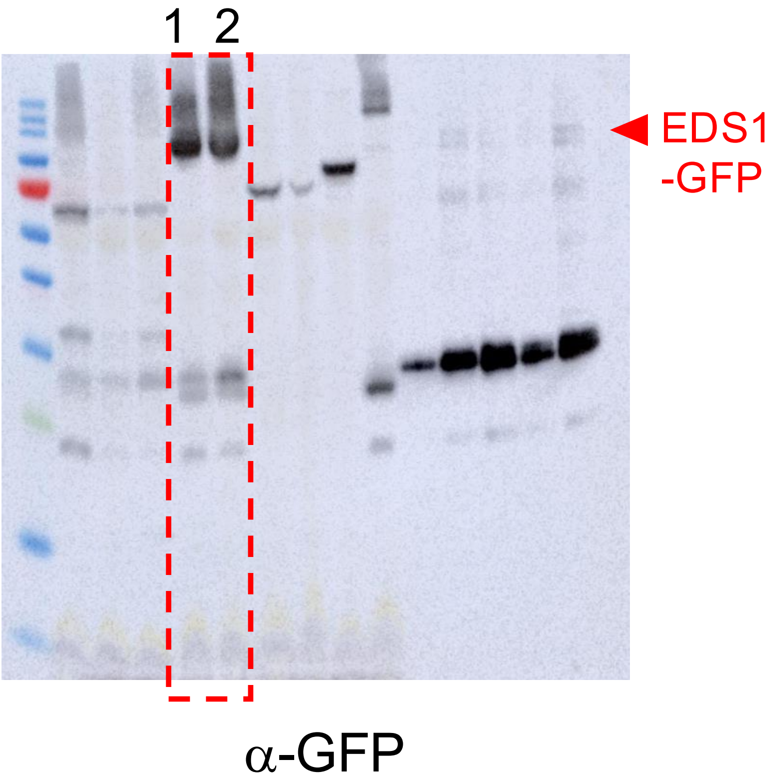

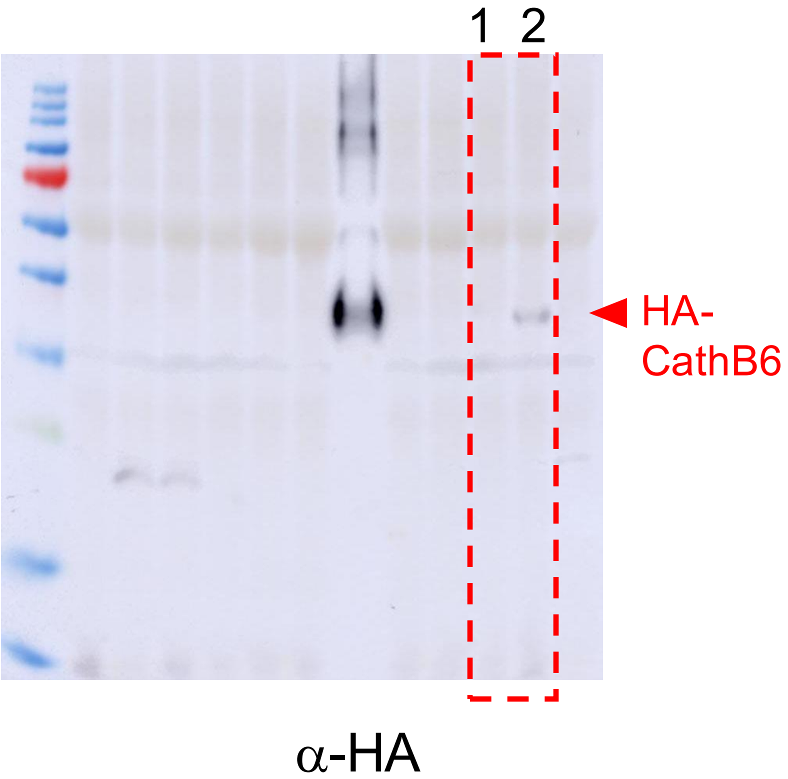

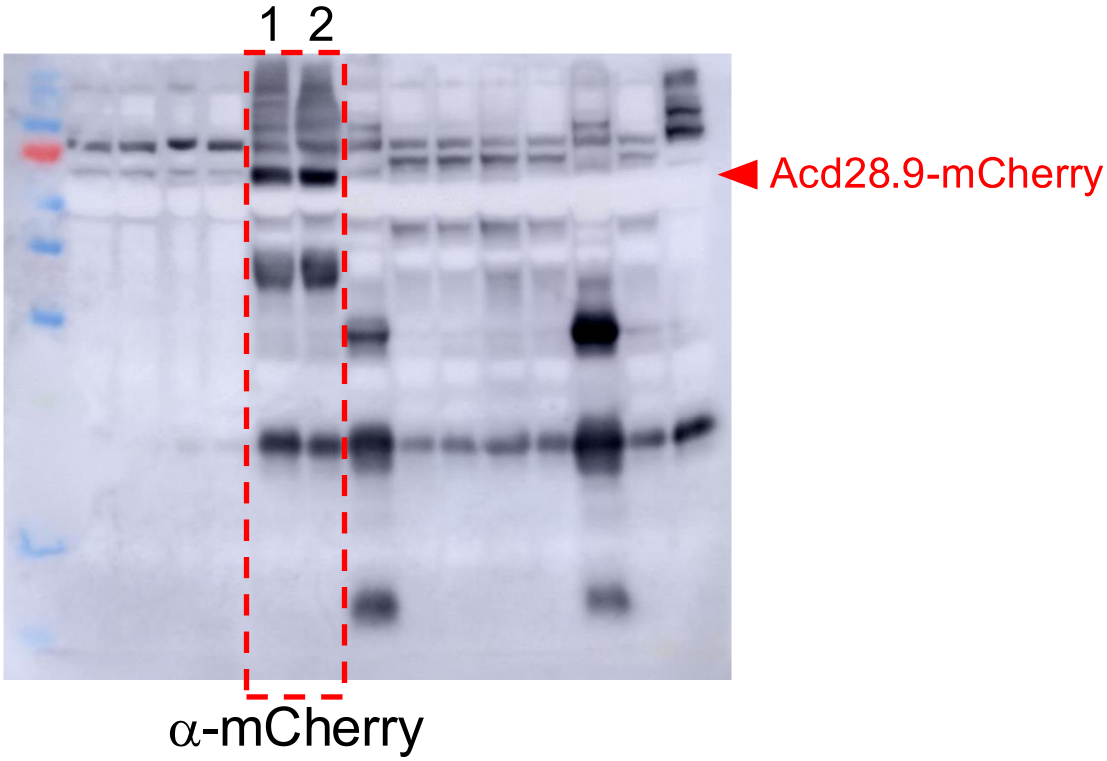

REFERENCES AND NOTES

1. J. Drohojowska, J. Szwedo, D. Żyła, D. Y. Huang, P. Müller, Fossils reshape the Sternorrhyncha evolutionary tree (Insecta, Hemiptera). *Sci. Rep.* **10**, 11390 (2020).

2. W. F. Tjallingii, T. H. Esch, Fine structure of aphid stylet routes in plant tissues in correlation with EPG signals. *Physiol. Entomol.* **18**, 317–328 (1993).

3. S. T. Mugford, E. Barclay, C. Drurey, K. C. Findlay, S. A. Hogenhout, An immuno-suppressive aphid saliva protein is delivered into the cytosol of plant mesophyll cells during feeding. *Mol. Plant Microbe Interact.* **29**, 854–861 (2016).

4. J. I. Bos, D. Prince, M. Pitino, M. E. Maffei, J. Win, S. A. Hogenhout, A functional genomics approach identifies candidate effectors from the aphid species *Myzus persicae* (green peach aphid). *PLoS Genet.* **6**, e1001216 (2010).

5. S. Snoeck, N. Guayazán-Palacios, A. D. Steinbrenner, Molecular tug-of-war: Plant immune recognition of herbivory. *Plant Cell* **34**, 1497–1513 (2022).

6. A. E. Whitfield, B. W. Falk, D. Rotenberg, Insect vector-mediated transmission of plant viruses. *Virology* **479-480**, 278–289 (2015).

7. S. Ray, C. L. Casteel, Effector-mediated plant-virus-vector interactions. *Plant Cell* **34**, 1514–1531 (2022).

8. CABI, *Myzus persicae* (green peach aphid) (CABI Compendium, 2022).

9. K. Ogawa, T. Miura, Aphid polyphenisms: Trans-generational developmental regulation through viviparity. *Front. Physiol.* **5**, 1 (2014).

10. Y. Chen, A. Singh, G. G. Kaithakottil, T. C. Mathers, M. Gravino, S. T. Mugford, C. van Oosterhout, D. Swarbreck, S. A. Hogenhout, An aphid RNA transcript migrates systemically within plants and is a virulence factor. *Proc. Natl. Acad. Sci. U.S.A.* **117**, 12763–12771 (2020).

11. T. C. Mathers, Y. Chen, G. Kaithakottil, F. Legeai, S. T. Mugford, P. Baa-Puyoulet, A. Bretaudeau, B. Clavijo, S. Colella, O. Collin, T. Dalmay, T. Derrien, H. Feng, T. Gabaldón, A. Jordan, I. Julca, G. J. Kettles, K. Kowitwanich, D. Lavenier, P. Lenzi, S. Lopez-Gomollon, D. Loska, D. Mapleson, F. Maumus, S. Moxon, D. R. Price, A. Sugio, M. van Munster, M. Uzest, D. Waite, G. Jander, D. Tagu, A. C. Wilson, C. van Oosterhout, D. Swarbreck, S. A. Hogenhout, Rapid transcriptional plasticity of duplicated gene clusters enables a clonally reproducing aphid to colonise diverse plant species. *Genome Biol.* **18**, 27 (2017).

12. H. Guo, Y. Zhang, J. Tong, P. Ge, Q. Wang, Z. Zhao, K. Zhu-Salzman, S. A. Hogenhout, F. Ge, Y. Sun, An aphid-secreted salivary protease activates plant defense in phloem. *Curr. Biol.* **30**, 4826–4836.e7 (2020).

13. Q. Liu, J. K. Goldberg, S. T. Mugford, G. Saalbach, C. Martins, A. Singh, G. G. Kaithakotti, D. Swarbreck, S. A. Hogenhout, The salivary proteome of the green peach aphid/peach-potato aphid (*Myzus persicae*) (Sulzer, 1776) (Hemiptera, Aphididae), Zenodo (2024); https://doi.org/10.5281/zenodo.13269257.

14. N. Aggarwal, B. F. Sloane, Cathepsin B: Multiple roles in cancer. *Proteomics Clin. Appl.* **8**, 427–437 (2014).

15. S. Verma, R. Dixit, K. C. Pandey, Cysteine proteases: Modes of activation and future prospects as pharmacological targets. *Front. Pharmacol.* **7**, 107 (2016).

16. J. Xu, J. Y. Yang, Q. W. Niu, N. H. Chua, Arabidopsis DCP2, DCP1, and VARICOSE form a decapping complex required for postembryonic development. *Plant Cell* **18**, 3386–3398 (2006).

17. A. Steffens, B. Jaegle, A. Tresch, M. Hülskamp, M. Jakoby, Processing-body movement in Arabidopsis depends on an interaction between myosins and DECAPPING PROTEIN1. *Plant Physiol.* **164**, 1879–1892 (2014).

18. B. Petre, D. G. Saunders, J. Sklenar, C. Lorrain, K. V. Krasileva, J. Win, S. Duplessis, S. Kamoun, Heterologous expression screens in *Nicotiana benthamiana* identify a candidate

effector of the wheat yellow rust pathogen that associates with processing bodies. *PLOS ONE* **11**, e0149035 (2016).

19. M. Kosmacz, M. Luzarowski, O. Kerber, E. Leniak, E. Gutiérrez-Beltrán, J. C. Moreno, M. Gorka, J. Szlachetko, D. Veyel, A. Graf, A. Skirycz, Interaction of 2′,3′-cAMP with Rbp47b plays a role in stress granule formation. *Plant Physiol.* **177**, 411–421 (2018).

20. T. Ibrahim, V. Khandare, F. G. Mirkin, Y. Tumtas, D. Bubeck, T. O. Bozkurt, AlphaFold2-multimer guided high-accuracy prediction of typical and atypical ATG8-binding motifs. *PLoS Biol.* **21**, e3001962 (2023).

21. Y. Pu, D. C. Bassham, Detection of autophagy in plants by fluorescence microscopy. *Methods Mol. Biol.* **2581**, 135–147 (2023).

22. R. Zavaliev, R. Mohan, T. Chen, X. Dong, Formation of NPR1 condensates promotes cell survival during the plant immune response. *Cell* **182**, 1093–1108.e18 (2020).

23. M. Brengues, D. Teixeira, R. Parker, Movement of eukaryotic mRNAs between polysomes and cytoplasmic processing bodies. *Science* **310**, 486–489 (2005).

24. A. Kearly, A. D. L. Nelson, A. Skirycz, M. Chodasiewicz, Composition and function of stress granules and P-bodies in plants. *Semin. Cell Dev. Biol.* **156**, 167–175 (2024).

25. G. J. Jang, J. Y. Yang, H. L. Hsieh, S. H. Wu, Processing bodies control the selective translation for optimal development of Arabidopsis young seedlings. *Proc. Natl. Acad. Sci. U.S.A.* **116**, 6451–6456 (2019).

26. J. Xu, N. H. Chua, Arabidopsis decapping 5 is required for mRNA decapping, P-body formation, and translational repression during postembryonic development. *Plant Cell* **21**, 3270–3279 (2009).

27. Q. Liu, J. Huang, A. C. M. Neefjes, C. Martins, G. Saalbach, S. A. Hogenhout, The proximity interactome of the peach-potato aphid (*Myzus persicae*) cathepsin B in *Arabidopsis thaliana*, Zenodo (2024); https://doi.org/10.5281/zenodo.14181687.

28. D. Lapin, D. D. Bhandari, J. E. Parker, Origins and immunity networking functions of EDS1 family proteins. *Annu. Rev. Phytopathol.* **58**, 253–276 (2020).

29. J. A. Dongus, J. E. Parker, EDS1 signalling: At the nexus of intracellular and surface receptor immunity. *Curr. Opin. Plant Biol.* **62**, 102039 (2021).

30. A. Nair, I. Goyal, E. Voß, P. Mrozek, S. Prajapati, C. Thurow, L. Tietze, K. Tittmann, C. Gatz, N-hydroxypipecolic acid-induced transcription requires the salicylic acid signaling pathway at basal SA levels. *Plant Physiol.* **187**, 2803–2819 (2021).

31. S. Wagner, J. Stuttmann, S. Rietz, R. Guerois, E. Brunstein, J. Bautor, K. Niefind, J. E. Parker, Structural basis for signaling by exclusive EDS1 heteromeric complexes with SAG101 or PAD4 in plant innate immunity. *Cell Host Microbe* **14**, 619–630 (2013).

32. A. V. García, S. Blanvillain-Baufumé, R. P. Huibers, M. Wiermer, G. Li, E. Gobbato, S. Rietz, J. E. Parker, Balanced nuclear and cytoplasmic activities of EDS1 are required for a complete plant innate immune response. *PLoS Pathog.* **6**, e1000970 (2010).

33. S. C. Saile, P. Jacob, B. Castel, L. M. Jubic, I. Salas-Gonzáles, M. Bäcker, J. D. G. Jones, J. L. Dangl, F. El Kasmi, Two unequally redundant "helper" immune receptor families mediate Arabidopsis thaliana intracellular "sensor" immune receptor functions. *PLoS Biol.* **18**, e3000783 (2020).

34. Z. Wu, L. Tian, X. Liu, Y. Zhang, X. Li, TIR signal promotes interactions between lipase-like proteins and ADR1-L1 receptor and ADR1-L1 oligomerization. *Plant Physiol.* **187**, 681–686 (2021).

35. H. Cui, E. Gobbato, B. Kracher, J. Qiu, J. Bautor, J. E. Parker, A core function of EDS1 with PAD4 is to protect the salicylic acid defense sector in Arabidopsis immunity. *New Phytol.* **213**, 1802–1817 (2017).

36. M. Giolai, "Spatially resolved transcriptomics reveals plant host responses to the aphid pest *Myzus persicae*," thesis, University of East Anglia (2019).

37. M. Gravino, S. T. Mugford, D. Pontiggia, J. Joyce, C. Drurey, D. C. Prince, F. Cervone, G. D. Lorenzo, S. A. Hogenhout, Aphid effector Mp10 balances immune suppression and defence activation through EDS1-dependent modulation of plant DAMP responses. bioRxiv 640334 [Preprint] (2025). https://doi.org/10.1101/2025.02.26.640334.

38. J. A. Dongus, D. D. Bhandari, M. Patel, L. Archer, L. Dijkgraaf, L. Deslandes, J. Shah, J. E. Parker, The Arabidopsis PAD4 lipase-like domain is sufficient for resistance to green peach aphid. *Mol. Plant Microbe Interact.* **33**, 328–335 (2019).

39. J. A. Dongus, D. D. Bhandari, E. Penner, D. Lapin, S. C. Stolze, A. Harzen, M. Patel, L. Archer, L. Dijkgraaf, J. Shah, H. Nakagami, J. E. Parker, Cavity surface residues of PAD4 and SAG101 contribute to EDS1 dimer signaling specificity in plant immunity. *Plant J.* **110**, 1415–1432 (2022).

40. W. J. Thomas, C. A. Thireault, J. A. Kimbrel, J. H. Chang, Recombineering and stable integration of the *Pseudomonas syringae* pv. *syringae* 61 hrp/hrc cluster into the genome of the soil bacterium *Pseudomonas fluorescens* Pf0-1. *Plant J.* **60**, 919–928 (2009).

41. N. Mukhi, H. Brown, D. Gorenkin, P. Ding, A. R. Bentham, C. E. M. Stevenson, J. D. G. Jones, M. J. Banfield, Perception of structurally distinct effectors by the integrated WRKY domain of a plant immune receptor. *Proc. Natl. Acad. Sci. U.S.A.* **118**, e2113996118 (2021).

42. D. Lapin, V. Kovacova, X. Sun, J. A. Dongus, D. Bhandari, P. von Born, J. Bautor, N. Guarneri, J. Rzemieniewski, J. Stuttmann, A. Beyer, J. E. Parker, A coevolved EDS1-SAG101-NRG1 module mediates cell death signaling by TIR-domain immune receptors. *Plant Cell* **31**, 2430–2455 (2019).

43. K. J. Kloth, J. Busscher-Lange, G. L. Wiegers, W. Kruijer, G. Buijs, R. C. Meyer, B. R. Albrectsen, H. J. Bouwmeester, M. Dicke, M. A. Jongsma, SIEVE ELEMENT-LINING CHAPERONE1 restricts aphid feeding on arabidopsis during heat stress. *Plant Cell* **29**, 2450–2464 (2017).

44. K. J. Kloth, P. Shah, C. Broekgaarden, C. Ström, B. R. Albrectsen, M. Dicke, SLI1 confers broad-spectrum resistance to phloem-feeding insects. *Plant Cell Environ.* **44**, 2765–2776 (2021).

45. Y. Ge, Y. M. Cai, L. Bonneau, V. Rotari, A. Danon, E. A. McKenzie, H. McLellan, L. Mach, P. Gallois, Inhibition of cathepsin B by caspase-3 inhibitors blocks programmed cell death in Arabidopsis. *Cell Death Differ.* **23**, 1493–1501 (2016).

46. B. Elsässer, F. B. Zauner, J. Messner, W. T. Soh, E. Dall, H. Brandstetter, Distinct roles of catalytic cysteine and histidine in the protease and ligase mechanisms of human legumain as revealed by DFT-Based QM/MM simulations. *ACS Catal.* **7**, 5585–5593 (2017).

47. M. S. McQueney, B. Y. Amegadzie, K. D'Alessio, C. R. Hanning, M. M. McLaughlin, D. McNulty, S. A. Carr, C. Ijames, J. Kurdyla, C. S. Jones, Autocatalytic activation of human cathepsin K. *J. Biol. Chem.* **272**, 13955–13960 (1997).

48. R. A. van der Hoorn, M. A. Leeuwenburgh, M. Bogyo, M. H. Joosten, S. C. Peck, Activity profiling of papain-like cysteine proteases in plants. *Plant Physiol.* **135**, 1170–1178 (2004).

49. P. Werelusz, S. Galiniak, M. Mołoń, Molecular functions of moonlighting proteins in cell metabolic processes. *Biochim. Biophys. Acta Mol. Cell Res.* **1871**, 119598 (2024).

50. C. J. Jeffery, Enzymes, pseudoenzymes, and moonlighting proteins: Diversity of function in protein superfamilies. *FEBS J.* **287**, 4141–4149 (2020).

51. Y. Luo, Z. Na, S. A. Slavoff, P-bodies: Composition, properties, and functions. *Biochemistry* **57**, 2424–2431 (2018).

52. P. Schwenk, A. Hiltbrunner, Phytochrome a mediates the disassembly of processing bodies in far-red light. *Front. Plant Sci.* **13**, 828529 (2022).

53. W. R. Brothers, F. Ali, S. Kajjo, M. R. Fabian, The EDC4-XRN1 interaction controls P-body dynamics to link mRNA decapping with decay. *EMBO J.* **42**, e113933 (2023).

54. J. Louis, Q. Leung, V. Pegadaraju, J. Reese, J. Shah, PAD4-dependent antibiosis contributes to the ssi2-conferred hyper-resistance to the green peach aphid. *Mol. Plant Microbe Interact.* **23**, 618–627 (2010).

55. W. R. H. Huang, M. Joosten, Immune signaling: Receptor-like proteins make the difference. *Trends Plant Sci.* **30**, 54–68 (2024).

56. J. Zönnchen, J. Gantner, D. Lapin, K. Barthel, L. Eschen-Lippold, J. L. Erickson, S. L. Villanueva, S. Zantop, C. Kretschmer, M. Joosten, J. E. Parker, R. Guerois, J. Stuttmann, EDS1 complexes are not required for PRR responses and execute TNL-ETI from the nucleus in *Nicotiana benthamiana*. *New Phytol.* **236**, 2249–2264 (2022).

57. H. Adachi, M. P. Contreras, A. Harant, C. H. Wu, L. Derevnina, T. Sakai, C. Duggan, E. Moratto, T. O. Bozkurt, A. Maqbool, J. Win, S. Kamoun, An N-terminal motif in NLR immune receptors is functionally conserved across distantly related plant species. *eLife* **8**, e49956 (2019).

58. E. L. Baggs, J. G. Monroe, A. S. Thanki, R. O'Grady, C. Schudoma, W. Haerty, K. V. Krasileva, Convergent loss of an EDS1/PAD4 signaling pathway in several plant lineages reveals coevolved components of plant immunity and drought response. *Plant Cell* **32**, 2158–2177 (2020).

59. C. Rispe, M. Kutsukake, V. Doublet, S. Hudaverdian, F. Legeai, J. C. Simon, D. Tagu, T. Fukatsu, Large gene family expansion and variable selective pressures for cathepsin B in aphids. *Mol. Biol. Evol.* **25**, 5–17 (2008).

60. W. W. de Jong, G. J. Caspers, J. A. Leunissen, Genealogy of the α-crystallin—Small heat-shock protein superfamily. *Int. J. Biol. Macromol.* **22**, 151–162 (1998).

61. E. R. Waters, E. Vierling, Plant small heat shock proteins–evolutionary and functional diversity. *New Phytol.* **227**, 24–37 (2020).

62. S. A. Whitham, R. J. Anderberg, S. T. Chisholm, J. C. Carrington, Arabidopsis RTM2 gene is necessary for specific restriction of tobacco etch virus and encodes an unusual small heat shock-like protein. *Plant Cell* **12**, 569–582 (2000).

63. B. Fenton, J. T. Margaritopoulos, G. L. Malloch, S. P. Foster, Micro-evolutionary change in relation to insecticide resistance in the peach–potato aphid, *Myzus persicae*. *Ecol. Entomol.* **35**, 131–146 (2010).

64. T. C. Mathers, R. H. M. Wouters, S. T. Mugford, D. Swarbreck, C. van Oosterhout, S. A. Hogenhout, Chromosome-scale genome assemblies of aphids reveal extensively rearranged autosomes and long-term conservation of the X chromosome. *Mol. Biol. Evol.* **38**, 856–875 (2021).

65. A. Dereeper, V. Guignon, G. Blanc, S. Audic, S. Buffet, F. Chevenet, J. F. Dufayard, S. Guindon, V. Lefort, M. Lescot, J. M. Claverie, O. Gascuel, Phylogeny.fr: Robust phylogenetic analysis for the non-specialist. *Nucleic Acids Res.* **36**, W465–W469 (2008).

66. S. Challa, N. R. R. Neelapu, "Phylogenetic trees: Applications, construction, and assessment," in *Essentials of Bioinformatics, Volume III: In Silico Life Sciences: Agriculture*, K. R. Hakeem, N. A. Shaik, B. Banaganapalli, R. Elango, Eds. (Springer International Publishing, 2019), pp. 167–192.

67. I. Letunic, P. Bork, Interactive tree of life (iTOL) v5: An online tool for phylogenetic tree display and annotation. *Nucleic Acids Res.* **49**, W293–W296 (2021).

68. C. Chen, Y. Wu, J. Li, X. Wang, Z. Zeng, J. Xu, Y. Liu, J. Feng, H. Chen, Y. He, R. Xia, TBtools-II: A "one for all, all for one" bioinformatics platform for biological big-data mining. *Mol. Plant* **16**, 1733–1742 (2023).

69. D. G. Gibson, L. Young, R. Y. Chuang, J. C. Venter, C. A. Hutchison III, H. O. Smith, Enzymatic assembly of DNA molecules up to several hundred kilobases. *Nat. Methods* **6**, 343–345 (2009).

70. X. Liang, L. Peng, C. H. Baek, F. Katzen, Single step BP/LR combined Gateway reactions. *Biotechniques* **55**, 265–268 (2013).

71. N. Bechtold, *In planta Agrobacterium* mediated gene transfer by infiltration of adult *Arabidopsis thaliana* plants. *CR Acad. Sci. Paris Life Sci.* **316**, 1194–1199 (1993).

72. T. D. Schmittgen, K. J. Livak, Analyzing real-time PCR data by the comparative C(T) method. *Nat. Protoc.* **3**, 1101–1108 (2008).

73. S. D. Yoo, Y. H. Cho, J. Sheen, Arabidopsis mesophyll protoplasts: A versatile cell system for transient gene expression analysis. *Nat. Protoc.* **2**, 1565–1572 (2007).

74. C. B. McCarthy, V. Romanowski, A simplified method for the extraction of baculoviral DNA for PCR analysis: A practical application. *J. Virol. Methods* **148**, 286–290 (2008).

75. S. Weidtkamp-Peters, Y. Stahl, The use of FRET/FLIM to study proteins interacting with plant receptor kinases. *Methods Mol. Biol.* **1621**, 163–175 (2017).

76. Q. Liu, A. C. M. Neefjes, R. Kobylinska, S. A. Hogenhout, Confocal microscopy data associated with "Aphid effectors suppress plant immunity via recruiting defence proteins to processing bodies," Zenodo (2025); https://doi.org/10.5281/zenodo.15105505.

77. J. P. May, P. Z. Johnson, M. Ilyas, F. Gao, A. E. Simon, The multifunctional long-distance movement protein of *Pea Enation Mosaic Virus 2* protects viral and host transcripts from nonsense-mediated decay. *MBio* **11**, e00204-20 (2020).

78. R. Merret, J. Descombin, Y. T. Juan, J. J. Favory, M. C. Carpentier, C. Chaparro, Y. Y. Charng, J. M. Deragon, C. Bousquet-Antonelli, XRN4 and LARP1 are required for a heat-triggered mRNA decay pathway involved in plant acclimation and survival during thermal stress. *Cell Rep.* **5**, 1279–1293 (2013).

79. M. Y. Wang, J. B. Chen, R. Wu, H. L. Guo, Y. Chen, Z. J. Li, L. Y. Wei, C. Liu, S. F. He, M. D. Du, Y. L. Guo, Y. L. Peng, J. D. G. Jones, D. Weigel, J. H. Huang, W. S. Zhu, The plant immune receptor SNC1 monitors helper NLRs targeted by a bacterial effector. *Cell Host Microbe* **31**, 1792–1803.e7 (2023).

80. J. X. Leong, M. Raffeiner, D. Spinti, G. Langin, M. Franz-Wachtel, A. R. Guzman, J. G. Kim, P. Pandey, A. E. Minina, B. Macek, A. Hafrén, T. O. Bozkurt, M. B. Mudgett, F. Börnke, D. Hofius, S. Üstün, A bacterial effector counteracts host autophagy by promoting degradation of an autophagy component. *EMBO J.* **41**, e110352 (2022).

81. M. Bartsch, E. Gobbato, P. Bednarek, S. Debey, J. L. Schultze, J. Bautor, J. E. Parker, Salicylic acid-independent ENHANCED DISEASE SUSCEPTIBILITY1 signaling in Arabidopsis immunity and cell death is regulated by the monooxygenase FMO1 and the Nudix hydrolase NUDT7. *Plant Cell* **18**, 1038–1051 (2006).

82. D. Jirage, T. L. Tootle, T. L. Reuber, L. N. Frost, B. J. Feys, J. E. Parker, F. M. Ausubel, J. Glazebrook, Arabidopsis thaliana PAD4 encodes a lipase-like gene that is important for salicylic acid signaling. *Proc. Natl. Acad. Sci. U.S.A.* **96**, 13583–13588 (1999).

83. J. M. Alonso, A. N. Stepanova, T. J. Leisse, C. J. Kim, H. Chen, P. Shinn, D. K. Stevenson, J. Zimmerman, P. Barajas, R. Cheuk, C. Gadrinab, C. Heller, A. Jeske, E. Koesema, C. C. Meyers, H. Parker, L. Prednis, Y. Ansari, N. Choy, H. Deen, M. Geralt, N. Hazari, E. Hom, M. Karnes, C. Mulholland, R. Ndubaku, I. Schmidt, P. Guzman, L. Aguilar-Henonin, M. Schmid, D. Weigel, D. E. Carter, T. Marchand, E. Risseeuw, D. Brogden, A. Zeko, W. L. Crosby, C. C. Berry, J. R. Ecker, Genome-wide insertional mutagenesis of *Arabidopsis thaliana*. *Science* **301**, 653–657 (2003).
